# PAnno: A pharmacogenomics annotation tool for clinical genomic testing

**DOI:** 10.3389/fphar.2023.1008330

**Published:** 2023-01-26

**Authors:** Yaqing Liu, Zipeng Lin, Qingwang Chen, Qiaochu Chen, Leqing Sang, Yunjin Wang, Leming Shi, Li Guo, Ying Yu

**Affiliations:** ^1^ State Key Laboratory of Genetic Engineering, Human Phenome Institute, School of Life Sciences and Shanghai Cancer Center, Fudan University, Shanghai, China; ^2^ Department of Breast Surgery, Precision Cancer Medicine Center, Key Laboratory of Breast Cancer in Shanghai, Fudan University Shanghai Cancer Center, Shanghai, China; ^3^ Department of Oncology, Shanghai Medical College, Fudan University, Shanghai, China; ^4^ State Key Laboratory of Multiphase Complex Systems, Institute of Process Engineering, Chinese Academy of Sciences, Beijing, China; ^5^ School of Chemical Engineering, University of Chinese Academy of Sciences, Beijing, China

**Keywords:** pharmacogenomics, drug responce, genomics, diplotype, haplotype, star allele, drug-genotype, precision medicine

## Abstract

**Introduction:** Next-generation sequencing (NGS) technologies have been widely used in clinical genomic testing for drug response phenotypes. However, the inherent limitations of short reads make accurate inference of diplotypes still challenging, which may reduce the effectiveness of genotype-guided drug therapy.

**Methods:** An automated Pharmacogenomics Annotation tool (PAnno) was implemented, which reports prescribing recommendations and phenotypes by parsing the germline variant call format (VCF) file from NGS and the population to which the individual belongs.

**Results:** A ranking model dedicated to inferring diplotypes, developed based on the allele (haplotype) definition and population allele frequency, was introduced in PAnno. The predictive performance was validated in comparison with four similar tools using the consensus diplotype data of the Genetic Testing Reference Materials Coordination Program (GeT-RM) as ground truth. An annotation method was proposed to summarize prescribing recommendations and classify drugs into avoid use, use with caution, and routine use, following the recommendations of the Clinical Pharmacogenetics Implementation Consortium (CPIC), etc. It further predicts phenotypes of specific drugs in terms of toxicity, dosage, efficacy, and metabolism by integrating the high-confidence clinical annotations in the Pharmacogenomics Knowledgebase (PharmGKB). PAnno is available at https://github.com/PreMedKB/PAnno.

**Discussion:** PAnno provides an end-to-end clinical pharmacogenomics decision support solution by resolving, annotating, and reporting germline variants.

## 1 Introduction

Next generation sequencing (NGS) based clinical genomic testing has become a powerful strategy in precision therapeutics (Ji et al., 2018; Malone et al., 2020; Tafazoli et al., 2021a). In the area of pharmacogenomics (PGx), it is reflected explicitly in the identification and annotation of germline genetic variants functioning in the absorption, distribution, metabolism, and elimination (ADME) of drugs (Goldstein et al., 2003; Klein et al., 2019). These variants largely contribute to the inter-individual differences in pharmacokinetics or drug response phenotypes (Rodrigues et al., 2019). Based on the genotype-phenotype association, the efficacy and toxicity of drugs can be revealed with a view to tailoring effective and safe drugs, as well as providing sensible advice on the dosage (Ashley, 2016; Roden et al., 2019).

A pharmacogenetic *allele* (*allele, star allele) or *haplotype* is composed of one or more genetic variants on the same chromosome, and a *diplotype* is formed by a pair of haplotypes of the same gene on homologous chromosomes (Robarge et al., 2007; Lingjun et al., 2014). In addition, *genotype* in this paper refers to variants detected by sequencing platform that will be identified as alleles (haplotypes) and then assigned as diplotypes. The diplotypes bridge the transition from genotype to phenotype and are the basis for precise drug administration. For instance, patients with a homozygous UGT1A1*28 (*28/*28 diplotype) are poor metabolizers, which leads to irreversible toxic effects when taking Belinostat, and require starting with a lower dose (Peer et al., 2016).

Accurate inference of diplotypes is the basis for precise personal genomic interpretation. However, the inability to distinguish parental origin and short-read characteristics of NGS increase the difficulty of the analysis (Browning and Browning, 2011; Snyder et al., 2015; Jin et al., 2018; van der Lee et al., 2020). This is due to the fact that diplotypes often require a joint judgment based on the multiple variants on the maternal and paternal chromosomes. Given the complexity of diplotype phasing, computational tools have been developed, e.g., Astrolabe (Twist et al., 2016), Cyrius (Chen et al., 2021), Aldy (Numanagić et al., 2018), Stargazer (Lee et al., 2019), PharmCAT (Sangkuhl et al., 2020), StellarPGx (Twesigomwe et al., 2021), and lmPGX (Klanderman et al., 2022). Nevertheless, there is potential to further improve the accuracy of diplotypes inference for NGS-derived variant call format (VCF) files, which are currently widely used in clinical genomic testing.

Annotation of diplotypes for drug responses phenotypes is another crucial aspect of the PGx clinical application. PharmCAT and lmPGX focus on more than a dozen genes associated with drug metabolizing enzymes and transporter proteins, and translate diplotypes into metabolizer phenotypes on a gene-by-gene basis to correlate dosing guidelines. However, sometimes more than one diplotypes are concerned with a drug, and determining drug dosage based on a single diplotype may be one-sided (Tasa et al., 2019; Shugg et al., 2020). As more and more genotype-phenotype associations have been identified, the number of PGx-related genes and alleles has expanded. For example, the number of genes in the Clinical Pharmacogenetics Implementation Consortium (CPIC) with high-confidence evidence (level A and B) has grown to 33 (CPIC, 2022). In this context, there is a growing need to develop computational tools to more comprehensively report the alleles that may affect drug response.

To address the above issues, we built the Pharmacogenomics Annotation tool (PAnno), an end-to-end automated tool for clinical genomic testing oriented to providing prescribing recommendations and predicting drug response phenotypes. PAnno takes a standard germline VCF file as input to identify PGx-relevant diplotypes. To achieve a more accurate inference, we developed a ranking model considering the differences in allele frequency between populations. In addition, we proposed an annotation method for potential phenotypes that takes into account the one-to-many relationship between drugs and diplotypes by integrating the clinical annotations of the Pharmacogenomics Knowledgebase (PharmGKB). Finally, PAnno provides a summay of the above results in an HTML report.

## 2 Materials and methods

### 2.1 The architecture of PAnno

**FIGURE 1:**
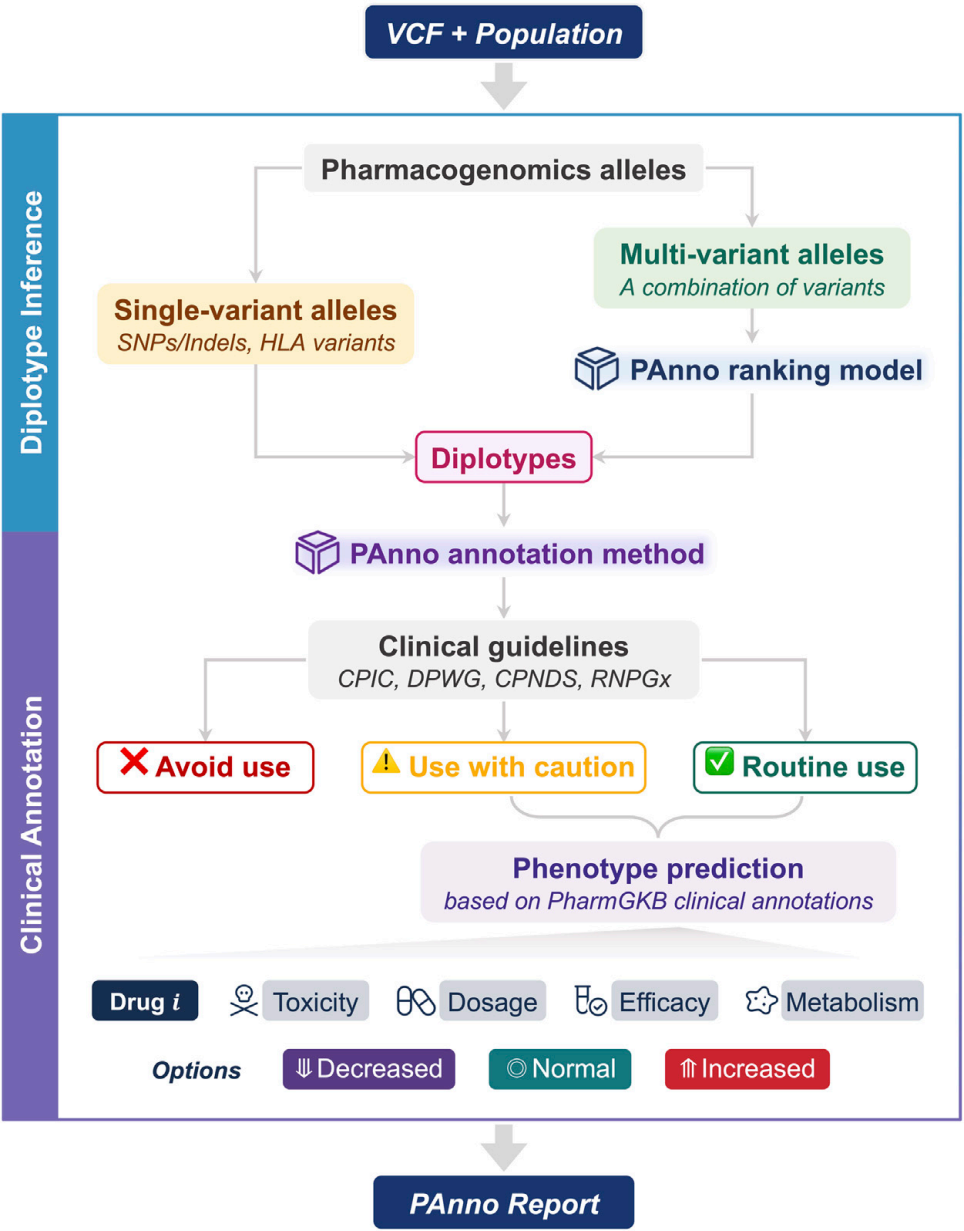
The architecture of PAnno. PAnno parses the user-submitted VCF with its affiliated population and outputs a pharmacogenomics (PGx) report. PAnno classifies PGx alleles in the VCF into single-variant alleles and multi-variant alleles. Diplotypes for the former can be derived directly, while the latter will call the PAnno ranking model for inference. Based on the inferred diplotypes, the PAnno annotation method first determines the availability of drugs according to clinical guidelines. Afterwards, it integrates the effects of multiple diplotypes on the same drug to predict drug response phenotypes in terms of toxicity, dosage, efficacy, and metabolism.

PAnno consists of two components, diplotype inference, and clinical annotation. By parsing the germline VCF file from NGS and the population to which the individual belongs, PAnno will output the prescribing recommendation, detail of inferred diplotypes, and drug response phenotypes in the form of an HTML report (Figure 1).

#### 2.1.1 Diplotype inference

This component aims to identify PGx alleles on each chromosome and infer the diplotypes from the user-submitted VCF file. PAnno first extracts all allele-related variants from the VCF file based on the pre-collected GRCh38 genomic coordinates of PGx alleles. Next, PAnno classifies the alleles into two categories: single-variant and multi-variant. Single-variant alleles constitute diplotypes that do not involve the judgment of multiple variants and the corresponding genes generally have not yet been standardized by a nomenclature committee, such as rs9923231 for VKORC1. Therefore, PAnno obtains their diplotypes based on the genotype information of the VCF file (e.g., the GT field), and reports according to the locations measured. In addition, PAnno determines the presence and copy number of HLA alleles if the relevant results are provided in the VCF. Multi-variant alleles need to take into account more than one variant on the same chromosome. The most likely diplotype of the gene of interest is inferred by the PAnno ranking model.

#### 2.1.2 Clinical annotation

This component aims to translate inferred diplotypes into phenotypes to provide prescribing recommendations and predict drug responses. The PAnno annotation method first annotates diplotypes with dosing guidelines to determine drug availability. For available drugs, clinical annotations of PharmGKB are integrated to predict the possible changes in toxicity, dosage, efficacy, and metabolism of a drug when a patient is routinely taking it.

### 2.2 Underlying data foundation

#### 2.2.1 Allele definition and population allele frequency

PAnno ranking model relied on the allele definition and population frequency of diplotypes. The PharmGKB (Whirl-Carrillo et al., 2012; Whirl-Carrillo et al., 2021), the Pharmacogene Variation (PharmVar) Consortium (Gaedigk et al., 2021), and the CPIC (Relling and Klein, 2011; Caudle et al., 2014; Relling et al., 2020) are the primary data resource for PAnno to inference diplotypes.

Moreover, PAnno followed the standardized grouping system applied by PharmGKB to divide the population into nine biogeographic groups, namely African American/Afro-Caribbean (AAC), American (AME), Central/South Asian (SAS), East Asian (EAS), European (EUR), Latino (LAT), Near Eastern (NEA), Oceanian (OCE), and Sub-Saharan African (SSA) (Huddart et al., 2019).

We first obtained the allele definitions and their population frequencies from the PharmGKB website, which provided a further integration of the frequencies compiled by PharmGKB and CPIC based on the published literature reports (Nofziger et al., 2020; Botton et al., 2021; Desta et al., 2021; Sangkuhl et al., 2021; PharmGKB, 2022e; Ramsey et al., 2022; Rodriguez-Antona et al., 2022). For genes for which PharmGKB did not provide population allele frequencies, we estimated the allele frequencies using variant frequencies provided by the 1000 Genomes Project (Siva, 2008). Specifically, we calculated the population frequencies separately for all variants defining each haplotype, and then used the lowest value to represent the frequency for the haplotype. It is worth noting that a null value for population frequency is more likely to result from insufficient data or studies. In contrast, a frequency of zero indicates that the population is essentially unlikely to have the haplotype. In this case, we assigned a minimal value (*epsilon = 1e-5*) to the null value.

On this basis, we calculated diplotype-related data based on the following two principles. First, the combination of any two haplotypes of a gene gives all possible diplotypes. Second, the combination of all variants involved in two haplotypes equals the definition of the corresponding diplotype. Finally, we annotated the population frequencies to each diplotype of each gene according to the Hardy-Weinberg equilibrium (Hardy, 1908; Weinberg, 1908).

#### 2.2.2 Prescribing recommendations and clinical annotations

Reliable PGx knowledge is the foundation for effective annotation and reporting. We collected the clinical guidelines integrated by PharmGKB and manually reviewed the prescribing recommendations. For given diplotypes, we annotated whether the drug in question needs to be avoided, used with caution, or used routinely according to the associated guidelines. The original prescribing information was primarily published by CPIC (Clancy et al., 2014; Martin et al., 2014; Muir et al., 2014; Relling et al., 2014; Birdwell et al., 2015; Hicks et al., 2015; Gammal et al., 2016; Saito et al., 2016; Bell et al., 2017; Hicks et al., 2017; Johnson et al., 2017; Amstutz et al., 2018; Goetz et al., 2018; Phillips et al., 2018; Brown et al., 2019; Desta et al., 2019; Gonsalves et al., 2019; Relling et al., 2019; Theken et al., 2020; Crews et al., 2021; Karnes et al., 2021; Lima et al., 2021; Cooper-DeHoff et al., 2022; Lee et al., 2022; McDermott et al., 2022), the Dutch Pharmacogenetics Working Group (DPWG) (Lunenburg et al., 2020; Brouwer et al., 2021; Matic et al., 2021; DPWG, 2022a; DPWG, 2022b), the Canadian Pharmacogenomics Network for Drug Safety (CPNDS) (Amstutz et al., 2014; Shaw et al., 2015; Aminkeng et al., 2016; Lee et al., 2016; Drögemöller et al., 2019), and the French National Network for Pharmacogenetics (RNPGx) (Lamoureux and Duflot, 2017; Picard et al., 2017; Quaranta et al., 2017; Quaranta and Thomas, 2017; Woillard et al., 2017).

In addition, we collected the clinical annotations from PharmGKB which summarize the published evidence for the association between a genetic variant and a drug. Each clinical annotation is assigned a level of evidence including 1A, 1B, 2A, 2B, 3, and 4, from high to low confidence (PharmGKB, 2022d). The variant-drug associations described by 1A, 1B, 2A, and 2B have been demonstrated in multiple trials and even incorporated into dosing guidelines. To improve interpretation reliability and clinical usability, PAnno only included the annotations from these four levels.

A PharmGKB clinical annotation generally corresponds to a drug-genotype pair, which describes in paragraph form what phenotypic response a person would have if they had a certain genotype. We determined the drug response reflected in the annotation paragraph by regular expressions with manual curation. Inspired by the CIPC standardization project, each annotation was classified into three phenotype terms, namely decreased function, normal function, and increased function (Caudle et al., 2017). For a given clinical description, such as the annotation of rs121434568 GG in https://www.pharmgkb.org/clinicalAnnotation/981420042. Our annotated result was that patients taking gefitinib with rs121434568 GG were marked with “increased” in terms of efficacy. To better integrate multiple annotations in practice, these phenotypes were numerically coded as 0.5 (decreased), 1 (normal), and 2 (increased), respectively.

### 2.3 PAnno ranking model for diplotype inference

**FIGURE 2:**
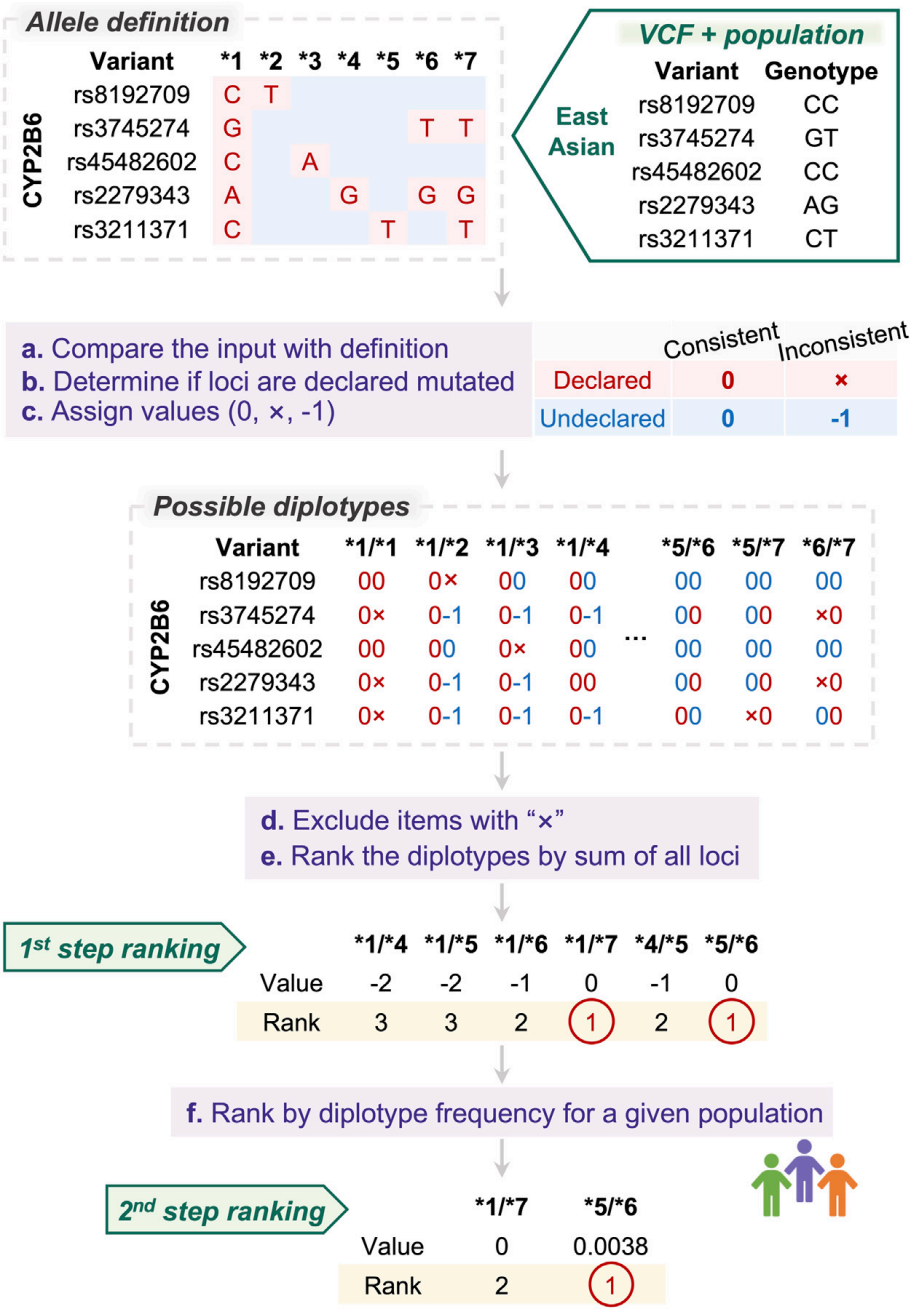
PAnno ranking model for diplotype inference. PAnno ranking model integrates a two-step ranking to determine the priority of diplotypes. In the first step, the consistency of the input alleles with the definition of candidate diplotypes is calculated. Diplotypes with the highest concordance are selected and ranked in the second step based on frequency in a given population.

PAnno ranking model takes as input genotypes of the positions at which alleles are defined. Candidate diplotypes for each gene will be prioritized by a two-step ranking as described below (Figure 2).

The definitions of alleles that form diplotypes are manually curated by experts. In general, an allele will have variants that are included in the definition of specific positions, which we refer to as “declared.” Loci not included in a haplotype definition are “undeclared.” We presume that the sequences of these undeclared loci are consistent with the wild type.

PAnno first compares the input genotypes with the definition of alleles, and assigns values based on their concordance and whether these loci are declared to be mutated. At declared loci, consistent is marked as “0” and inconsistent as “×”. At undeclared loci, consistent is marked as “0” and inconsistent as “−1.” According to this principle, comparison values will be assigned on all variants of all possible diplotypes.

Next, PAnno excludes diplotypes containing “×”, and calculates the sum of the values of all variants for each diplotype. The higher the total score, i.e., the closer to zero, the more consistent the patient mutation is with the diplotype definition.

Finally, PAnno performs two consecutive steps of ranking. The first ranking is based on the total consistency score. The highest ranked candidates are further ranked according to their frequency in the given population. The diplotypes with the highest rank are the final result of the PAnno ranking model.

### 2.4 PAnno annotation method for predicting drug response phenotypes

The PAnno annotation method interprets the inferred diplotypes with the associated drugs based on the clinical guidelines of CPIC, DPWG, CPNDS, and RNPGx. Drugs will be classified into three categories: 1) Avoid use. Avoidance of a drug is clearly stated in the prescribing recommendations for the given diplotype. 2) Use with caution. Prescribing changes are recommended for the given diplotype, e.g., dose adjustment and alternative medication. In addition, prescribing recommendations that differ in specific populations or require consideration of multiple diplotypes are included in this category. 3) Routine use. There is no recommended prescribing change for the given diplotype.

For a single drug, there may be multiple associated genotypes, i.e., multiple PharmGKB clinical annotations. To integrate the impact of multiple genotypes, PAnno calculates the mean of the annotation scores for all associated diplotypes for each clinically available drug. The integrated drug response phenotypes are indicated as decreased (<1), normal (=1), and increased (>1). Notably, drugs judged to be “avoid use” in the guideline-based annotation will not be included in the further interpretation.

Drug response phenotypes predicted by PAnno are further extrapolations beyond metabolizer phenotypes. It is necessary to interpret the PAnno phenotype concerning the corresponding four categories, i.e., toxicity, dosage, efficacy, and metabolism. For example, weak metabolizers tend to obtain higher toxicity after taking the drug at a regular dose, which the PAnno phenotype would be predicted as “decreased metabolism” and “increased toxicity.”

### 2.5 Test data for GeT-RM samples

The CDC Genetic Testing Reference Materials Coordination Program (GeT-RM) has previously conducted several pharmacogenetic studies to create publicly available genomic DNA reference materials for the field (Pratt et al., 2010; Pratt et al., 2016; Gaedigk et al., 2019; Gaedigk et al., 2022; Pratt et al., 2022). GeT-RM worked with members of the pharmacogenetic testing community to characterize the DNA materials using various techniques such as microarray and sequencing. Consensus diplotypes for a total of 29 PGx genes were developed and used as a reference dataset to assess the quality of PGx analysis. With the development of technology, GeT-RM has updated the results of these samples in earlier studies. Therefore, our evaluation will be based on the latest version of the consensus diplotypes obtained from https://www.cdc.gov/labquality/GeT-RM/inherited-genetic-diseases-pharmacogenetics/pharmacogenetics.html.

Among these GeT-RM studies, 137 samples had the most comprehensive PGx genetic assay results. The VCF files for 88 of these samples are freely available from the 1000 Genomes Project at http://ftp.1000genomes.ebi.ac.uk/vol1/ftp/data_collections/1000G_2504_high_coverage/working/20190425_NYGC_GATK/raw_calls_updated/. The germline variant data were re-sequenced and analyzed by the New York Genome Center at 30x coverage (Byrska-Bishop et al., 2021). These samples are from four biogeographic groups, including African American/Afro-Caribbean, East Asian, European, and Latino. The VCF files along with the corresponding population will be used as input to PAnno.

## 3 Results

### 3.1 Overview of PAnno

PAnno resolves and annotates PGx alleles based on the patient germline VCF file and the population information to provide prescribing recommendations and predict drug response phenotypes (Figure 1). To facilitate user access, PAnno has been developed as a Python package and a Conda package. The source code, the documentation, and the example reports are available at https://github.com/PreMedKB/PAnno.

In the current version, PAnno supports the parsing of diplotypes for 52 genes and the clinical annotation of 100 drugs (Supplementary Table S1). There are 13 genes involving multi-variant defined alleles with the PAnno ranking model applied, including CYP2B6, CYP2C19, CYP2C8, CYP2C9, CYP2D6, CYP3A4, CYP3A5, CYP4F2, DPYD, NUDT15, SLCO1B1, TPMT, and UGT1A1. It should be noted that alleles defined on copy number variants (CNV) and structural variants (SV) are not included in PAnno, because CNV and SV variations are not included in typical VCF files. Unincorporated alleles include CYP2B6*29, CYP2B6*30, CYP2C19*36, CYP2C19*37, CYP2D6*5, CYP2D6*13, CYP2D6*61, CYP2D6*63, CYP2D6*68, SLCO1B1*48, and SLCO1B1*49. Additionally, PAnno also includes the assignment of 17 HLA alleles of HLA-A, HLA-B, HLA-C, HLA-DPB1, and HLA-DRB1. The other alleles refer to the single-variant alleles, such as ABCG2, CACNA1S, IFNL3, VKORC1, RYR1, CFTR, etc., which are basically SNVs/Indels.

**FIGURE 3:**
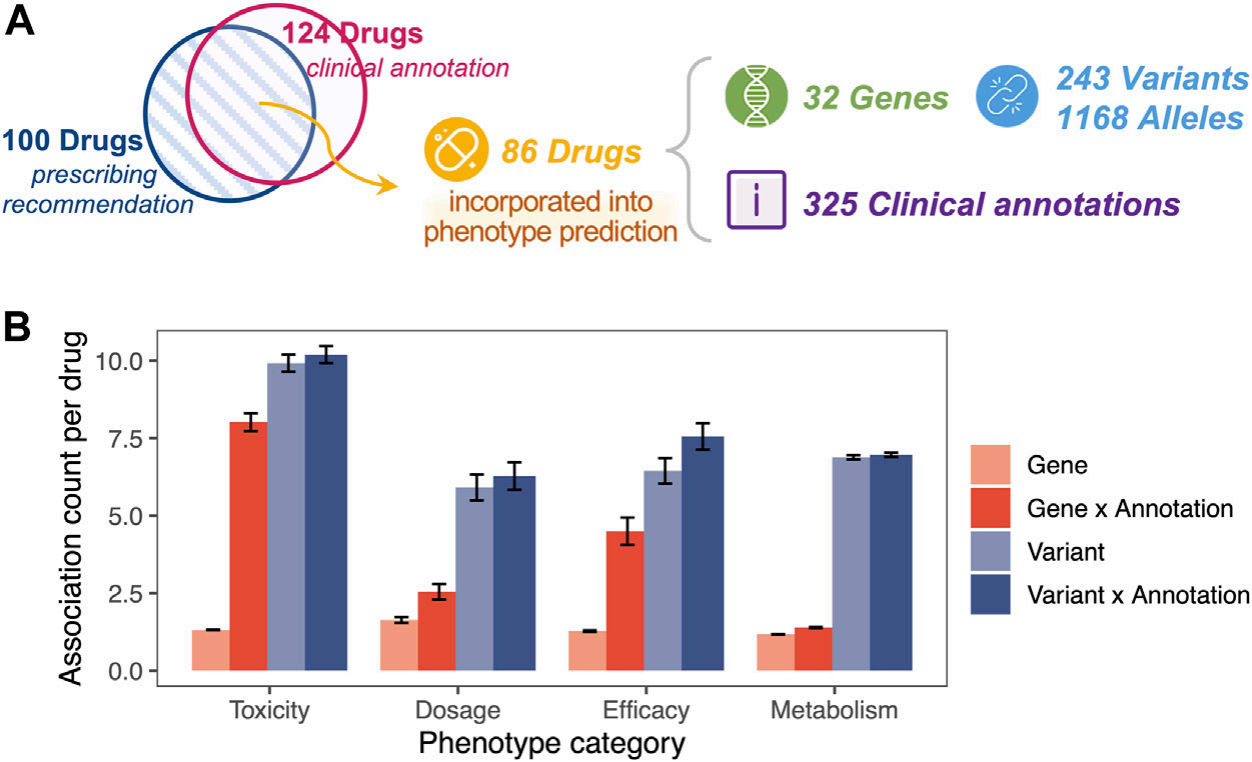
One-to-many clinical association for a given drug. **(A)** The PharmGKB clinical annotations used by PAnno covered 86 drugs, 32 genes, 243 variants, and 1168 alleles. The blue circle represents drugs covered in the prescribing recommendations of CPIC, DPWG, CPNDS, and RNPGx guidelines. The pink circle represented drugs involved in clinical annotations with evidence levels of 1A, 1B, 2A, and 2B in PharmGKB. **(B)** Average number of associations for a given drug in relation to genes (light red), combinations of genes and annotations (dark red), variants (light blue), and combinations of variants and annotations (dark blue). Error bars represent ±standard error of mean. The x-axis indicates the different phenotype categories, including toxicity, dosage, efficacy, and metabolism.

For clinical annotation, PAnno includes a total of 53 CPIC, 60 DPWG, six CPNDS, and eight RNPGx dosing guidelines, covering 28 genes and 100 drugs. PAnno further collected 325 PharmGKB clinical annotations, of which 282 were at level 1A, 14 at level 1B, 20 at level 2A, and nine at level 2B. These clinical annotations intersected with dosing guidelines for a total of 86 drugs, associated with 32 genes, 243 variants, and 1168 alleles (Figure 3A).

### 3.2 One-to-many clinical associations for a given drug

One drug was observed with multiple genetic associations in the PharmGKB clinical annotation used by PAnno, even when distinguishing phenotypic categories (Figure 3B). In terms of toxicity, dosage, efficacy, and metabolism, the average number of genes associated with one drug was approximately 1.31, 1.64, 1.28, 1.18, and the average number of variants associated was 9.90, 5.91, 6.28, 6.88, respectively. For a drug-gene pair or a drug-variant pair, there may be more than one clinical annotation. For example, PharmGKB provides two clinical annotations (PharmGKB ID 1449269910 and 1447673005) to describe the toxicity of warfarin in patients with different genotypes of VKORC1 rs9923231 (PharmGKB, 2022b; PharmGKB, 2022c). The results showed that a significant increase in the average number of gene-annotation combinations occurred for a specific drug, with 8.02, 2.55, 4.5, and 1.39 for toxicity, dosage, efficacy, and metabolism, respectively. Meanwhile, the increase in the number of variant-annotation combinations was not significant, at 10.2, 6.27, 7.06, and 6.96, respectively. The phenomenon of one-to-many associations with a given drug reinforces the importance of the drug-centric nature of the PAnno annotation method.

### 3.3 Prioritization of indistinguishable diplotypes based on population frequency

#### 3.3.1 Indistinguishable diplotypes for NGS-derived variants

NGS-derived clinical genomics testing data cannot determine exactly which chromosome the variant is located. When two haplotypes are combined, as occurs in a VCF, haplotypes can be inferred based on the allele definitions. When focusing only on the alleles, there may be cases where different diplotypes have the same mutation pattern, and we labeled them as “indistinguishable diplotypes.” We counted the number of haplotypes, diplotypes, and indistinguishable diplotypes for NGS for the 13 genes analyzed by PAnno (Table 1). There were eight genes (CYP2B6, CYP2C9, CYP2C19, CYP2D6, NUDT15, SLCO1B1, TMPT, and UGT1A1) containing indistinguishable diplotypes, which is one of the important reasons for PAnno to set up a further ranking step. The details are included in Supplementary Table S2.

**TABLE 1:** Number of indistinguishable diplotypes for NGS-derived variants.

Gene	Number of haplotypes	Number of diplotypes	Number of indistinguishable diplotypes
CYP2B6	35	630	20
CYP2C8	18	171	0
CYP2C9	75	2,850	2
CYP2C19	34	595	10
CYP2D6	136	9,316	56
CYP3A4	34	595	0
CYP3A5	6	21	0
CYP4F2	3	6	0
DPYD	83	3,486	0
NUDT15	20	210	2
SLCO1B1	42	903	47
TPMT	46	1,081	2
UGT1A1	10	55	6

#### 3.3.2 Differences in diplotype frequencies across populations

**FIGURE 4:**
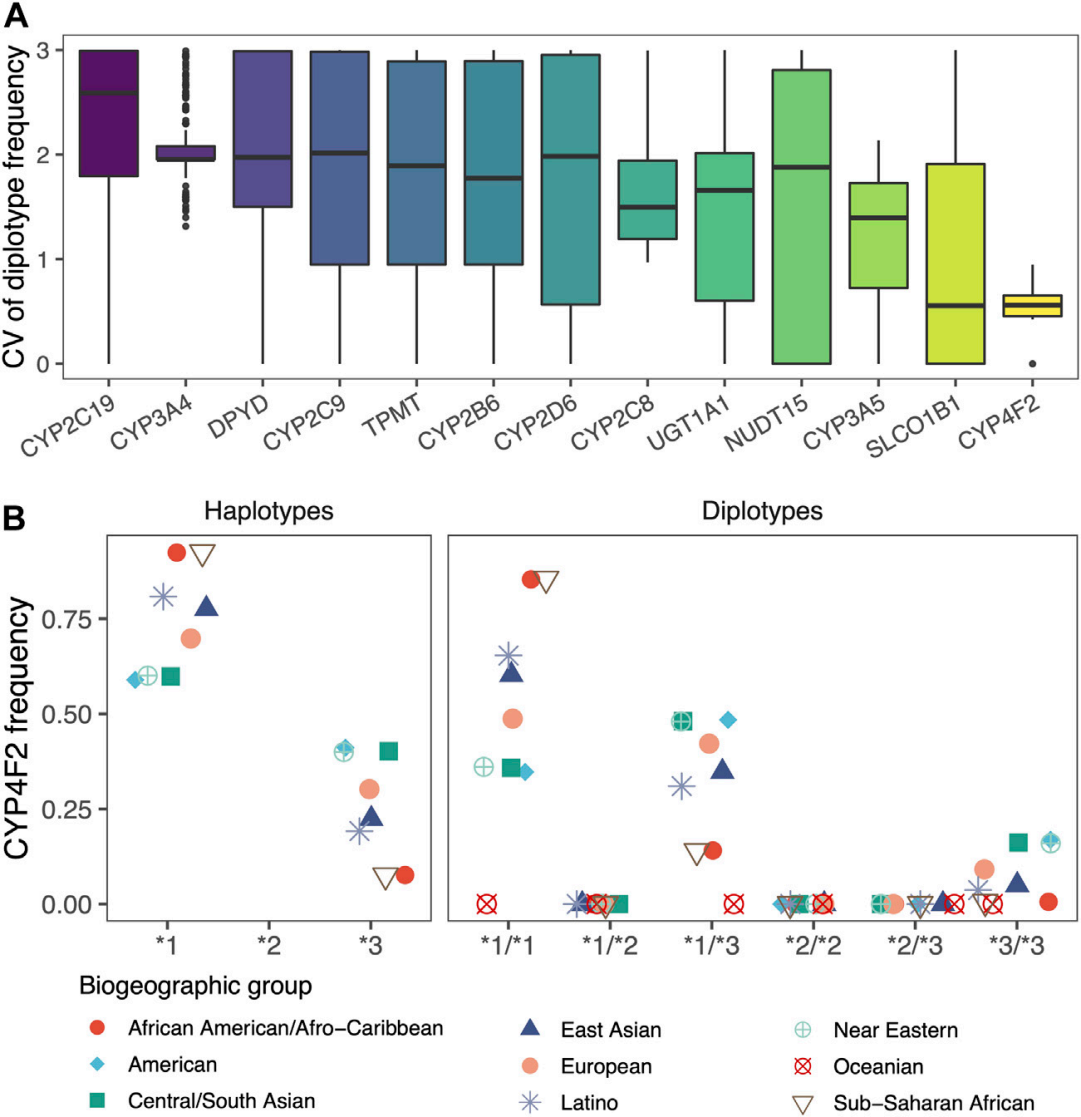
Differences in diplotype frequencies across populations. **(A)** Box plots showed the differences in the frequencies of diplotypes in 13 genes across the nine biogeographic groups, which are characterized by coefficients of variation (CV). **(B)** Scatter plot showed the haplotype frequencies of CYP4F2 and the diplotype frequencies calculated according to Hardy-Weinberg equilibrium. Jittering was applied to avoid overlapping.

The frequencies of the vast majority of diplotypes in the nine populations had significant differences. For each gene, the average coefficient of variation (CV) of the frequencies in different populations ranged from 0.53 to 2.24. Among them, CYP2C19, CYP3A4, and DPYD had mean CVs greater than 2, indicating that diplotypes of these genes are extremely unlikely to occur in different populations. In addition, we found that CYP2C8 and CYP3A4, which were inferred from the lowest allele frequency based on the 1000 Genomes Project, performed similarly to other genes (Figure 4A).

Even for CYP4F2 with the smallest variability, its haplotypes and diplotypes reflected significant population differences. The population frequency of diplotype obtained by random combinations would be jointly influenced by the two haplotypes (Figure 4B). PAnno takes advantage of the above features to prioritize candidate diplotypes that are indistinguishable based on NGS variants.

### 3.4 Performance of PAnno in diplotype inference

#### 3.4.1 Concordance between PAnno results and GeT-RM consensus

To evaluate the performance of PAnno in diplotype inference, we applied the ranking model to germline variant data from 88 test samples and calculated the concordance of diplotypes for 14 genes shared in PAnno and GeT-RM (Table 2). Notably, there are differences between the haplotypes included in GeT-RM and PAnno, i.e., CYP2C19*38 is not in GeT-RM, while is considered a wild-type haplotype in PAnno. A detailed comparison of the differences in the allele definition can be found in Supplementary Table S3. Therefore, if the output haplotypes were not included by both GeT-RM and PAnno or did not have a clear conversion relationship, the relevant samples were all excluded.

**TABLE 2:** Performance of PAnno and similar tools in diplotype inference.

Gene	% PAnno	% PharmCAT	% lmPGX	% Adly	% StellarPGx
CYP2B6	89.53 (77/86)				66.22
CYP2C8	100.00 (88/88)			100.00	100.00
CYP2C9	98.86 (87/88)	100.00	95.70	99.27	100.00
CYP2C19	100.00 (88/88)	100.00	95.70	93.43	97.30
CYP2D6	94.74 (54/57)		94.30	99.08	78.10
CYP3A4	100.00 (40/40)			95.62	100.00
CYP3A5	100.00 (88/88)	100.00	100.00	100.00	100.00
CYP4F2	100.00 (64/64)	80.00	80.30	92.70	95.52
DPYD	100.00 (42/42)	94.92	98.30	57.66	
NUDT15	100.00 (3/3)				
SLCO1B1	70.77 (46/65)	8.47	62.90		
TPMT	100.00 (86/86)	98.31	98.60	98.54	
UGT1A1	56.25 (36/64)	53.33			

Overall, the diplotypes predicted by PAnno were highly concordant with the ground truth of GeT-RM. The concordance rates of diplotypes for CYP2C19, CYP2C8, CYP3A4, CYP3A5, CYP4F2, DPYD, and TPMT were 100%, while for CYP2B6, CYP2C9, CYP2D6, SLCO1B1, and UGT1A1 were 89.53%, 98.86%, 94.74%, 70.77%, and 56.25%, respectively. A detailed description of the consistency concordance is in Supplementary Table S4. The reasons for incomplete concordance can be summarized as two main aspects, i.e., the iteration of the analysis pipeline leading to the VCF files containing variants not found in GeT-RM and the linkage disequilibrium (LD) among haplotypes.

For CYP2B6, one haplotype was inconsistent in seven samples due to the detection of variants defining *4, *5, or *22 in the VCF files, while GeT-RM considered these samples as *1. Two other samples matched both *1/*7 and *5/*6, and due to the high frequency of *5/*6 in the population to which the samples belonged, PAnno did not output *1/*7 as advocated by GeT-RM.

For CYP2C9, an inconsistent sample HG01190 was judged as *2/*61 in GeT-RM, while PAnno inferred it as *1/*61. Similar to the reason for the inconsistency in the seven samples of CYP2B6, the results in the VCF showed a C>T heterozygous transition at rs1799853, which was not satisfying the requirement of *2/*61, i.e., C>T homozygous transition at rs1799853.

For CYP2D6, the inconsistency in the three samples was also due to inconsistent detection of germline variants. GeT-RM considered HG00589 as *1/*21 while PAnno inferred *1/*2 owing to its VCF not covering one of the mutated loci that defined *21 (G>GG at rs72549352). The VCF file of NA18519 contained the variant associated with *106 (C>T at rs28371733) while GeT-RM did not, resulting in a PAnno-inferred diplotype as *29/*106 and GeT-RM as *1/*29. For the diplotype of NA19174, GeT-RM considered *4/*40 while PAnno inferred *4/*58. The main difference between these two diplotypes is that the variant sequence of *40 on rs72549358 is AAAGGGGCG(3) while *58 is AAAGGGGCG(2). The 1000 Genome Project VCF for this sample showed a variant on rs72549358 as AAAGGGGCG(2), which led to the inconsistency.

For SLCO1B1, PAnno inferred the diplotypes of the three inconsistent samples as *1/*14 because the heterozygous variants defining *14 (A>G at rs2306283 and C>A at rs11045819) were identified. However, GeT-RM considered that no variant occurred and thus judged them as *1/*1. Going back to the original data of GeT-RM, we found that there was a platform where *14 was also inferred, while GeT-RM did not use the results of this platform. The rest of discordance was also since PAnno inferred that the 16 samples carried *37 (A>G at rs2306283) while GeT-RM did not. Fundamentally, it still lies in the difference in variant detection between the GeT-RM project and the VCF files of the 1000 Genomes Project used by PAnno.

For UGT1A1, *60 with *28, *80 with *28, and *80 with *37 have a very high probability of LD (Innocenti et al., 2002; Chen et al., 2014; Gammal et al., 2016). Thus, PAnno extrapolated 12 samples that were labeled *1/*28 in GeT-RM as *60/*80+*28, six samples labeled *28/*28 as *80+*28/*80+*28, eight samples labeled *28/*60 as *28/*80, one sample labeled *28/(*37) as *80+*28/*80+*37, and one sample labeled *27/*28 as (*27; *60)/*80+*28. This phenomenon was also found in PharmCAT and was shown to be largely unaffected by the differences in diplotypes with GeT-RM caused by LD on the determination of drug response.

#### 3.4.2 Comparison with other similar tools

We compared the performance of PAnno with four other diplotype phasing tools, including PharmCAT, lmPGX, Adly, and StellarPGx (Table 2). The accuracy of the above tools was derived from their respective publications, implying potential differences in how accuracy is judged. The empty cells in the table do not exactly mean that the software does not support the inference of the corresponding gene, but only that the comparison results with GeT-RM for the tool are not available. For example, PharmCAT supports genes such as CYP2B6 and NUDT15 in subsequent updates. In comparison, PAnno performed excellently in terms of both the number of PGx genes and the accuracy assessed based on GeT-RM consensus. This demonstrates the comparable performance of the PAnno ranking model compared to other excellent algorithms.

Although PAnno’s diplotype inference method was developed based on the allele definition, it not only performed exact matching as in PharmCAT and lmPGX but also incorporated population allele frequencies. This allows multiple filtering and ranking of candidate diplotypes. When an exact match to the diplotype is impossible, PAnno will output results that match the allele definition and have a higher probability of occurring in the population. Some variants associated with the haplotype definition were in complex formats or not covered by the current sequencing data. Therefore, they were not resolved successfully in the exact match reflected by the null “symbol.exact”, but PAnno still obtained highly consistent results with the GeT-RM consensus. This implies that PAnno may be somewhat tolerant of potential artifacts in variant calling, misfitting of the input format of VCF, etc. Supplementary Table S5 showed the analysis results for 13 genes from 88 test samples at different steps, including diplotypes that matched the definition exactly, diplotypes with the highest concordance with the definition (the first step ranking), diplotypes with the highest population frequency on this basis (the second step ranking).

### 3.5 PAnno report

**FIGURE 5:**
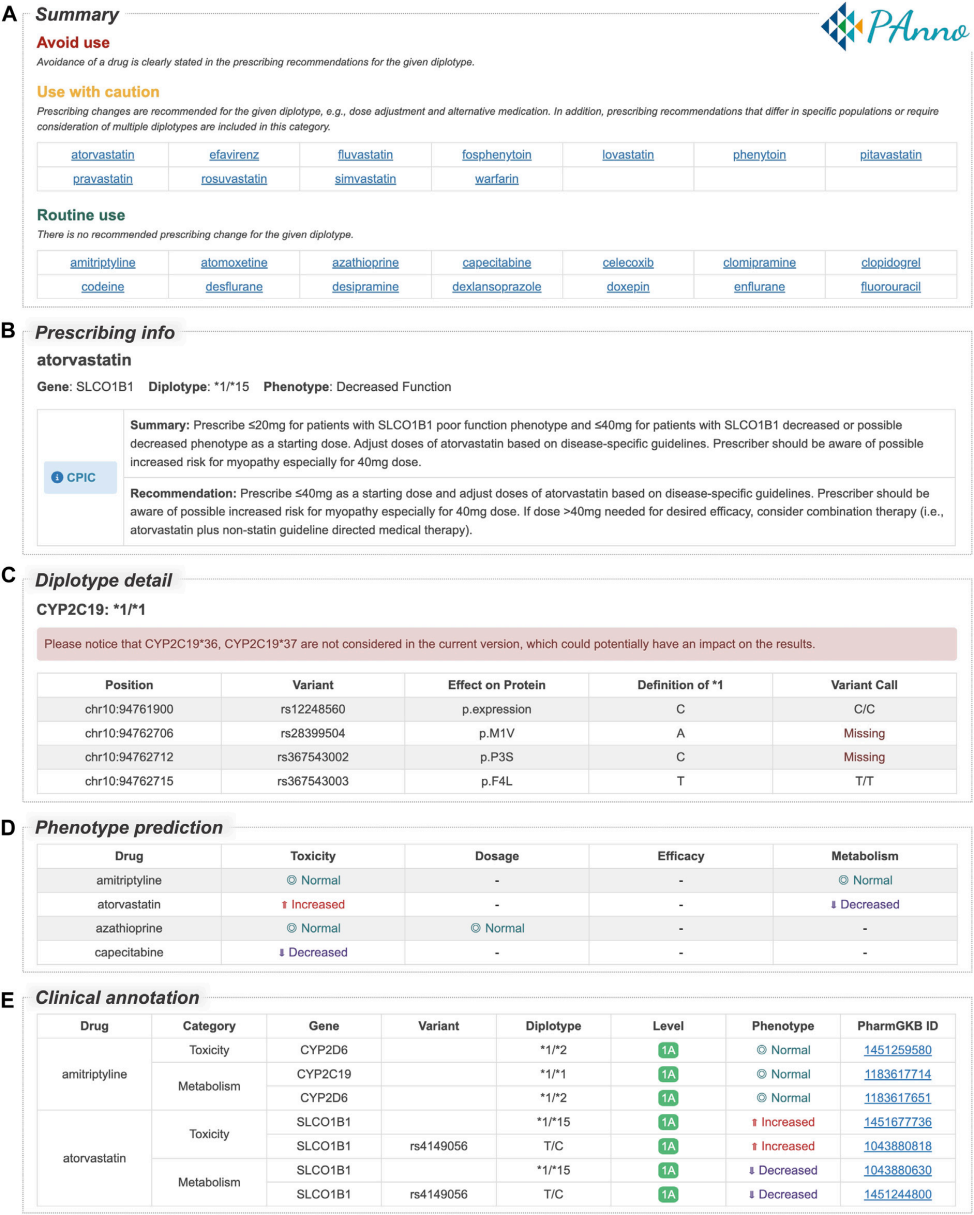
PAnno report sections for the 1000 Genomes Project VCF file of NA10859. PAnno report consists of five sections. **(A)** Summary. Drugs are classed into three categories based on whether clinical guidelines recommend a prescribing change for the given diplotypes, i.e., avoid use, use with caution, and routine use. **(B)** Prescribing info. For each drug, this section lists the associated gene, diplotype, phenotype, and recommendations in CPIC, DPWG, CPNDS, and RNPGx. **(C)** Diplotype detail. The table contains the detection of loci associated with the definition of diplotypes. **(D)** Phenotype prediction. The effects of multiple diplotypes for each clinically available drug in terms of toxicity, dosage, efficacy, and metabolism are described. **(E)** Clinical annotation. The original annotations for the predicted phenotypes in the previous section are presented in the table. Full HTML report of NA10859 is available in Supplementary Material S6.

PAnno summarizes the analysis results in an HTML report for better understanding by the user. A snapshot of the PAnno report of the 1000 Genomes Project VCF file for NA10859 is used here as an example (Figure 5). The report contains five core sections: 1) Summary (Figure 5A). Drugs are reported in three categories, namely, avoid use, use with caution, and routine use. This is based on whether clinical guidelines recommend a prescribing change for the given diplotypes. Although the current version of PAnno covers 100 drugs, the report shows only those drugs that the patient’s genotype is associated with. Hyperlinks provide direct access to the detailed recommendations in the next section. 2) Prescribing info (Figure 5B). This section is drug-centric and presents all drug-specific genes detected from the patient’s genetic profile, along with their corresponding PAnno-inferred diplotypes, CPIC phenotypes, and recommendations in CPIC, DPWG, CPNDS, and RNPGx. 3) Diplotype detail (Figure 5C). The inferred diplotypes for the 13 PGx genes are presented in this section. The detailed information includes the genomic coordinates, the defined and called variants at each position, and potential protein changes. In addition, the genotypes of the single-variant alleles are summarized in a table. 4) Phenotype prediction (Figure 5D). This section summarizes the predicted drug response phenotypes of the PAnno annotation method in terms of toxicity, dosage, efficacy, and metabolism, after integrating the multiple diplotypes associated with a clinically available drug. The predicted phenotypes are indicated as decreased, normal, and increased. 5) Clinical annotation (Figure 5E). The original PharmGKB clinical annotations on which the predicted phenotypes in the previous section are based are listed in a table. In addition, basic information such as sample name, reporting time, biogeographic group, and supplementary notes, will be presented at the beginning and end. PAnno reports of four samples from different populations are available in Supplementary Material S6. Reports for the 88 tested samples are accessible at https://github.com/PreMedKB/PAnno-analysis.

## 4 Discussion

PAnno is an interpretation tool for resolving individual germline genotypes to infer diplotypes and annotate pharmacogenomic knowledge. The frequency of PGx alleles varies across populations, which leads to differences in drug metabolism, transport, etc. (Gaedigk et al., 2017; Zhou et al., 2017; Ahsan et al., 2020; Jithesh et al., 2022). PAnno applied this feature to the PAnno ranking model built for the inference of multi-variant alleles, which facilitated the prioritization of indistinguishable diplotypes from NGS germline variant calling. The validity of PAnno for inferring diplotypes was demonstrated in comparison with the GeT-RM dataset and four similar tools. After resolving PGx-associated diplotypes, a PAnno annotation method was designed to associate clinical dosing guidelines based on the patient’s diplotypes and furthermore to integrate the effects of multiple diplotypes associated with a specific drug.

PAnno performs comparably to similar tools in terms of diplotype inference, but the accuracy is still insufficient for some genes involved in complex genomic regions, such as UGT1A1. This is mainly due to the limitation of NGS itself in identifying repetitive sequences, etc., which affects the final haplotype resolution (Mantere et al., 2019; Garg, 2021). However, the detection of CNVs and SVs is critical for the determination of some phenotypes, such as CYP2D6 ultrarapid metabolizers. Since PAnno directly analyzes VCF files, it cannot address the identification of the exact number of CNVs as well as the SVs of large segments. In the current version, some PGx alleles in CYP2B6, CYP2C19, CYP2D6, and SLCO1B1 cannot be analyzed by PAnno. Reviewing the 88 samples in GeT-RM for the above four genes, the diplotypes of CYP2D6 were involved in alleles not contained by PAnno. Specifically, 14 samples involved SVs (*5, *13, *68), six samples involved tandem arrangements, and eight samples involved CNVs. In total, they account for 35.23% of the overall tested samples, which is a non-negligible proportion (Supplementary Table S4). In this respect, PAnno is deficient compared to Aldy and StellarPGx because they do not rely only on VCF files for the inference of diplotypes. Currently, we have disclosed the non-coverable alleles of CYP2B6, CYP2C19, CYP2D6, and SLCO1B1 in the “diplotype detail” section of the PAnno report, and the compatibility of CNVs and SVs would be considered in the future.

The performance of PAnno is closely related to the quality of the VCF files. Quality here does not only refer to the rigorous filtering of VCFs, but refers broadly to the operational performance throughout the process of data generation and analysis. In this paper, PAnno’s predicted diplotypes using the VCF files of the 1000 Genomes Project have a small number of inconsistencies with the diplotype consensus of GeT-RM. After manual checking, we found many variants that clearly did not match the diplotypes proposed by GeT-RM even in the VCF files. In this case, we are unable to assert the reason for the discrepancies. Therefore, if the germline variants are detected inaccurately, it may raise the risk of inaccurate diplotype inference and even inappropriate clinical recommendations.

Finally, during the clinical annotation step, PAnno does not take into account the effect of disease type. PAnno annotation method only seeks relevant annotations around a specific drug on a specific phenotype category (e.g., toxicity), assigns scores, and integrates them. At this point, some annotations may only be relevant for a specific disease, which may affect the predicted drug response phenotype after integration. In addition, there is a significant amount of manual curation work that limits more precise development. For example, in the annotation of PharmGKB ID 1446846513, neoplasm is not caused by the drug but is the indication for taking anthracyclines (PharmGKB, 2022a). For such clinical annotations specifying indications, complementary disease information contributes to a more accurate pharmacogenomic interpretation. However, this requires laborious manual curation to distinguish indications from adverse effects after drug administration.

Overall, we developed PAnno to provide researchers and clinicians with informative recommendations to aid drug treatment decisions by linking genotypes and drug response phenotypes. The tool has shown great promise in the accuracy and broadness of the analysis of PGx alleles. Furthermore, PGx genes have progressively gone beyond the long-studied range of drug-metabolizing enzymes and transporters. Clinical evidence for nuclear receptors, transcription regulators, and genes potentially related to ADME, etc., is increasing (Arbitrio et al., 2021; Tafazoli et al., 2021b; Lanillos et al., 2022). As the accumulation of associations between different genotypes and drug responses, the integrative strategy for genotype resolution and clinical annotation advocated by PAnno would become more valuable. We believe that the comprehensive interpretation properties of PAnno make it possible to complement the PGx clinical genomic testing.

## Data Availability

The original contributions presented in the study are included in the article/Supplementary Material, further inquiries can be directed to the corresponding authors.

## Supplementary material

SUPPLEMENTARY TABLE S1 List of PGx genes included in PAnno.

SUPPLEMENTARY TABLE S2 Indistinguishable diplotypes when the parental origin uncertain.

SUPPLEMENTARY TABLE S3 Differences in haplotype definition of PAnno and GeT-RM.

SUPPLEMENTARY TABLE S4 Consistency of PAnno inferred diplotype and GeT-RM consensus.

SUPPLEMENTARY TABLE S5 Diplotypes at different stages of the PAnno ranking model.

SUPPLEMENTARY MATERIAL S6 PAnno reports of four samples from different populations.
