# Supplementary material for: PAnno: A pharmacogenomics annotation tool for clinical genomic testing
Source: Front Pharmacol. 2023 Jan 26;14:1008330. doi: 10.3389/fphar.2023.1008330 (PMC9909284; doi:10.3389/fphar.2023.1008330)
Supplement: Supplementary file 3 [file DataSheet1.ZIP › Supp.6 PAnno reports of four samples from different populations/NA19147.PAnno.html]

PAnno Report


# v0.3.1

- **Summary**
- **Prescribing Info**
- **Diplotype Detail**
- Multi-variant allele
- Single-variant allele
- **Phenotype Prediction**
- **Clinical Annotation**
- **About**

# 

An automated clinical pharmacogenomics annotation tool to report drug responses and prescribing recommendations by parsing the germline variants.

> Sample ID: NA19147  
> Biogeographic Group: African American/Afro-Caribbean (AAC)  
> Report Time: Wed Dec 28 22:36:14 2022

**Disclaimer:** The PAnno report iterates as the release version changes. In the current release, you should only use it to evaluate whether PAnno will compile and run properly on your system. All information in the report is interpreted directly from the uploaded VCF file. Users recognize that they use it at their own risk.

## **Summary**

Drugs are classified to indicate whether the clinical guidelines recommend a prescribing change based on the given diplotypes. Original prescribing information was collected by PharmGKB, primarily from the Clinical Pharmacogenetics Implementation Consortium (CPIC), the Dutch Pharmacogenetics Working Group (DPWG), the Canadian Pharmacogenomics Network for Drug Safety (CPNDS), the French National Network of Pharmacogenetics (RNPGx).

**Avoid use**  

Avoidance of a drug is clearly stated in the prescribing recommendations for the given diplotype.

|  |  |  |  |  |  |  |
| --- | --- | --- | --- | --- | --- | --- |
| amitriptyline | antidepressants | clomipramine | doxepin | imipramine | trimipramine | venlafaxine |

**Use with caution**  

Prescribing changes are recommended for the given diplotype, e.g., dose adjustment and alternative medication. In addition, prescribing recommendations that differ in specific populations or require consideration of multiple diplotypes are included in this category.

|  |  |  |  |  |  |  |
| --- | --- | --- | --- | --- | --- | --- |
| atazanavir | atomoxetine | atorvastatin | clopidogrel | codeine | desipramine | efavirenz |
| eliglustat | flecainide | fluvastatin | fosphenytoin | irinotecan | lansoprazole | lovastatin |
| metoprolol | nortriptyline | omeprazole | pantoprazole | phenytoin | pimozide | pitavastatin |
| pravastatin | propafenone | rasburicase | rosuvastatin | simvastatin | tacrolimus | tamoxifen |
| tramadol | voriconazole | warfarin | zuclopenthixol |  |  |  |

**Routine use**  

There is no recommended prescribing change for the given diplotype.

|  |  |  |  |  |  |  |
| --- | --- | --- | --- | --- | --- | --- |
| aripiprazole | azathioprine | brexpiprazole | capecitabine | celecoxib | desflurane | dexlansoprazole |
| enflurane | fluorouracil | flurbiprofen | fluvoxamine | haloperidol | halothane | hydrocodone |
| ibuprofen | isoflurane | lornoxicam | meloxicam | mercaptopurine | methoxyflurane | ondansetron |
| paroxetine | peginterferon alfa-2a | peginterferon alfa-2b | piroxicam | ribavirin | risperidone | sevoflurane |
| succinylcholine | tenoxicam | thioguanine | tropisetron |  |  |  |

## **Prescribing Info**

### **amitriptyline**

**Gene**: CYP2D6    **Diplotype**: \*17/\*29    **Phenotype**: Intermediate Metabolizer

|  |  |
| --- | --- |
| **DPWG** | **Summary:** The Dutch Pharmacogenetics Working Group Guideline for amitriptyline recommends decreasing the dose for CYP2D6 intermediate and CYP2D6 poor metabolizers and increasing the dose or using an alternative drug for CYP2D6 ultra-rapid metabolizers. |
|  | **Recommendation:** Use 75% of the standard dose and monitor the efficacy and side effects or the plasma concentrations of amitriptyline and nortriptyline to adjust the maintenance dose |

|  |  |
| --- | --- |
| **CPIC** | **Summary:** The CPIC Dosing Guideline update for amitriptyline recommends an alternative drug for CYP2D6 ultrarapid or poor metabolizers and CYP2C19 ultrarapid, rapid or poor metabolizers. If amitriptyline is warranted, consider a 50% dose reduction in CYP2D6 or CYP2C19 poor metabolizers. For CYP2D6 intermediate metabolizers, a 25% dose reduction should be considered. |
|  | **Recommendation:** Consider 25% reduction of recommended starting dose. Utilize therapeutic drug monitoring to guide dose adjustments. |

**Gene**: CYP2C19    **Diplotype**: \*1/\*17    **Phenotype**: Rapid Metabolizer

|  |  |
| --- | --- |
| **CPIC** | **Summary:** The CPIC Dosing Guideline update for amitriptyline recommends an alternative drug for CYP2D6 ultrarapid or poor metabolizers and CYP2C19 ultrarapid, rapid or poor metabolizers. If amitriptyline is warranted, consider a 50% dose reduction in CYP2D6 or CYP2C19 poor metabolizers. For CYP2D6 intermediate metabolizers, a 25% dose reduction should be considered. |
|  | **Recommendation:** Avoid tertiary amine use due to potential for sub-optimal response. Consider alternative drug not metabolized by CYP2C19. TCAs without major CYP2C19 metabolism include the secondary amines nortriptyline and desipramine. If a tertiary amine is warranted, utilize therapeutic drug monitoring to guide dose adjustments. |

### **antidepressants**

**Gene**: CYP2D6    **Diplotype**: \*17/\*29    **Phenotype**: Intermediate Metabolizer

|  |  |
| --- | --- |
| **RNPGx** | **Summary:** The French National Network of Pharmacogenetics (Réseau national de pharmacogénétique (RNPGx)) recommends CYP2D6 and CYP2C19 genotyping before initiating an antidepressant treatment, especially in patients with a high risk of toxicity. |
|  | **Recommendation:** Due to the risk of adverse effects that would require discontinuation of treatment, antidepressants should be avoided for patients exhibiting complete deficiency. Dose reduction to the order of 25-50% of standard dose can be proposed for patients with partial deficiency. Due to the current lack of biomarkers predictive of response or non-response to antidepressants, these tests can potentially be used to optimize treatment while limiting adverse effects. |

### **aripiprazole**

**Gene**: CYP2D6    **Diplotype**: \*17/\*29    **Phenotype**: Intermediate Metabolizer

|  |  |
| --- | --- |
| **DPWG** | **Summary:** The Royal Dutch Pharmacists Association - Pharmacogenetics Working Group (DPWG) recommends reducing maximum dose of aripiprazole for patients carrying poor metabolizer alleles of CYP2D6. |
|  | **Recommendation:** NO action is needed for this gene-drug interaction.The genetic variation increases the plasma concentration of the sum of aripiprazole and the active metabolite dehydroaripiprazole to a limited degree. There is insufficient evidence that this increases the risk of side effects. |

### **atazanavir**

**Gene**: UGT1A1    **Diplotype**: \*80+\*28/\*80+\*28    **Phenotype**: Poor Metabolizer

|  |  |
| --- | --- |
| **CPIC** | **Summary:** The CPIC dosing guideline recommends considering advising individuals who carry two decreased function UGT1A1 alleles about a substantial likelihood of developing jaundice, which may cause non-adherence. The dosing guideline recommends that alternative agents be considered if the risk of non-adherence due to jaundice is high. The risk of discontinuation is low and very low for individuals carrying one, or no decreased function UGT1A1 alleles, respectively. |
|  | **Recommendation:** Consider an alternative agent particularly where jaundice would be of concern to the patient. |

### **atomoxetine**

**Gene**: CYP2D6    **Diplotype**: \*17/\*29    **Phenotype**: Intermediate Metabolizer

|  |  |
| --- | --- |
| **DPWG** | **Summary:** The Dutch Pharmacogenetics Working Group Guideline for atomoxetine states for CYP2D6 ultrarapid metabolizers, to be alert to reduced efficacy of atomoxetine or select an alternative drug as a precaution. Be alert to ADEs in CYP2D6 poor metabolizers. |
|  | **Recommendation:** 1. In the event of side effects occurring and/or a response later than 9 weeks: reduce the dose and check whether the effect is conserved. The plasma concentration of atomoxetine is a factor of 2-3 times higher for IM than for EM at the same dose. |

### **atorvastatin**

**Gene**: SLCO1B1    **Diplotype**: \*20/\*30    **Phenotype**: Indeterminate

|  |  |
| --- | --- |
| **CPIC** | **Summary:** Prescribe ≤20mg for patients with SLCO1B1 poor function phenotype and ≤40mg for patients with SLCO1B1 decreased or possible decreased phenotype as a starting dose. Adjust doses of atorvastatin based on disease-specific guidelines. Prescriber should be aware of possible increased risk for myopathy especially for 40mg dose. |
|  | **Recommendation:** No recommendation. |

### **azathioprine**

**Gene**: TPMT    **Diplotype**: \*1/\*1    **Phenotype**: Normal Metabolizer

|  |  |
| --- | --- |
| **CPIC** | **Summary:** Consider an alternate agent or extreme dose reduction of azathioprine for patients who are TPMT or NUDT15 poor metabolizers. Start at 30-80% of target dose for patients who are TPMT or NUDT15 intermediate metabolizers. |
|  | **Recommendation:** Start with normal starting dose (e.g., 2-3 mg/kg/day) and adjust doses of azathioprine based on disease-specific guidelines. Allow 2 weeks to reach steady state after each dose adjustment. |

|  |  |
| --- | --- |
| **RNPGx** | **Summary:** Testing for TPMT genotype and phenotype is recommended for patients who are receiving azathioprine as an immunosuppressant. A dose reduction should be considered for patients with intermediate TPMT activity, while a greater dose reduction or selection of an alternate drug should be considered for patients with low TPMT activity. |
|  | **Recommendation:** Dose adjustment: Standard dose. Initial dose: Azathioprine 2.0-2.5 mg/kg/day. |

**Gene**: NUDT15    **Diplotype**: \*1/\*1    **Phenotype**: Normal Metabolizer

|  |  |
| --- | --- |
| **CPIC** | **Summary:** Consider an alternate agent or extreme dose reduction of azathioprine for patients who are TPMT or NUDT15 poor metabolizers. Start at 30-80% of target dose for patients who are TPMT or NUDT15 intermediate metabolizers. |
|  | **Recommendation:** Start with normal starting dose (e.g., 2-3 mg/kg/day) and adjust doses of azathioprine based on disease-specific guidelines. Allow 2 weeks to reach steady state after each dose adjustment. |

### **brexpiprazole**

**Gene**: CYP2D6    **Diplotype**: \*17/\*29    **Phenotype**: Intermediate Metabolizer

|  |  |
| --- | --- |
| **DPWG** | **Summary:** The Royal Dutch Pharmacists Association - Pharmacogenetics Working Group (DPWG) recommends to use half of the standard dose of brexpiprazole for patients carrying poor metabolizer alleles of CYP2D6. |
|  | **Recommendation:** NO action is required for this gene-drug interaction. There are indications supporting an increase in the exposure to brexpiprazole, but no indications supporting an increase in side effects in patients with this gene variation. |

### **capecitabine**

**Gene**: DPYD    **Diplotype**: c.85T>C (\*9A)/c.1349C>T    **Phenotype**: Normal Metabolizer

|  |  |
| --- | --- |
| **CPIC** | **Summary:** The CPIC Dosing Guideline for 5-fluorouracil and capecitabine recommends an alternative drug for patients who are DPYD poor metabolizers with an activity score of 0. In those who are poor metabolizers with an activity score of 0.5, an alternative drug is also recommended, but if this is not considered a suitable therapeutic option, 5-fluorouracil or capecitabine should be administered at a strongly reduced dose with early therapeutic drug monitoring. Patients who are intermediate metabolizers with an activity score of 1 or 1.5 should receive a dose reduction of 50%. Patients with the c.[2846A>T];[2846A>T] genotype may require a >50% dose reduction. |
|  | **Recommendation:** Based on genotype, there is no indication to change dose or therapy. Use label-recommended dosage and administration |

### **celecoxib**

**Gene**: CYP2C9    **Diplotype**: \*1/\*1    **Phenotype**: Normal Metabolizer

|  |  |
| --- | --- |
| **CPIC** | **Summary:** The CPIC Dosing Guideline for celecoxib, flurbiprofen, ibuprofen and lornoxicam recommends initiating therapy with 25-50% of the lowest recommended starting dose for CYP2C9 poor metabolizers and initiating therapy with lowest recommended starting dose for CYP2C9 intermediate metabolizers with activity score of 1. See full guideline for further details and supporting evidence. |
|  | **Recommendation:** Initiate therapy with recommended starting dose. In accordance with the prescribing information, use the lowest effective dosage for shortest duration consistent with individual patient treatment goals. |

### **clomipramine**

**Gene**: CYP2D6    **Diplotype**: \*17/\*29    **Phenotype**: Intermediate Metabolizer

|  |  |
| --- | --- |
| **CPIC** | **Summary:** Tricyclic antidepressants have comparable pharmacokinetic properties, it may be reasonable to apply the CPIC Dosing Guideline for amitriptyline and CYP2C19, CYP2D6 to other tricyclics including clomipramine. The CPIC Dosing Guideline update for amitriptyline recommends an alternative drug for CYP2D6 ultrarapid or poor metabolizers and CYP2C19 ultrarapid, rapid or poor metabolizers. If amitriptyline is warranted, consider a 50% dose reduction in CYP2D6 or CYP2C19 poor metabolizers. For CYP2D6 intermediate metabolizers, a 25% dose reduction should be considered. |
|  | **Recommendation:** Consider 25% reduction of recommended starting dose. Utilize therapeutic drug monitoring to guide dose adjustments. |

|  |  |
| --- | --- |
| **DPWG** | **Summary:** The Dutch Pharmacogenetics Working Group Guideline for clomipramine recommends dose changes and to monitor the effect and side effects and the plasma concentrations to set the maintenance dose for CYP2D6 poor (PM), and intermediate (IM) metabolizer or to avoid clomipramine in PM and ultrarapid (UM) metabolizer. |
|  | **Recommendation:** Use 70% of the standard dose and monitor the effect and side effects or the plasma concentrations of clomipramine and desmethylclomipramine. For depression, the therapeutic range is 200-400 ng/mL for the sum of the plasma concentrations of clomipramine and desmethylclomipramine. For anxiety disorders, the therapeutic plasma concentration of clomipramine is approximately 100 ng/mL, in combination with a plasma concentration of desmethylclomipramine lower than 200 ng/mLFor obsessive compulsive disorder, the therapeutic plasma concentration of clomipramine is higher than 200 ng/mL, in combination with a plasma concentration of desmethylclomipramine that is as low as possible. A sum of the plasma concentrations of clomipramine and desmethylclomipramine higher than 600 ng/mL is considered toxic. |

**Gene**: CYP2C19    **Diplotype**: \*1/\*17    **Phenotype**: Rapid Metabolizer

|  |  |
| --- | --- |
| **CPIC** | **Summary:** Tricyclic antidepressants have comparable pharmacokinetic properties, it may be reasonable to apply the CPIC Dosing Guideline for amitriptyline and CYP2C19, CYP2D6 to other tricyclics including clomipramine. The CPIC Dosing Guideline update for amitriptyline recommends an alternative drug for CYP2D6 ultrarapid or poor metabolizers and CYP2C19 ultrarapid, rapid or poor metabolizers. If amitriptyline is warranted, consider a 50% dose reduction in CYP2D6 or CYP2C19 poor metabolizers. For CYP2D6 intermediate metabolizers, a 25% dose reduction should be considered. |
|  | **Recommendation:** Avoid tertiary amine use due to potential for sub-optimal response. Consider alternative drug not metabolized by CYP2C19. TCAs without major CYP2C19 metabolism include the secondary amines nortriptyline and desipramine. If a tertiary amine is warranted, utilize therapeutic drug monitoring to guide dose adjustments. |

### **clopidogrel**

**Gene**: CYP2C19    **Diplotype**: \*1/\*17    **Phenotype**: Rapid Metabolizer

|  |  |
| --- | --- |
| **CPIC** | **Summary:** The CPIC Dosing Guideline for clopidogrel recommends an alternative antiplatelet therapy for CYP2C19 poor or intermediate metabolizers (cardiovascular indications: prasugrel or ticagrelor if no contraindication; neurovascular indications: alternative P2Y12 inhibitor if clinically indicated and no contraindication.) |
|  | **Recommendation:** If considering clopidogrel, use at standard dose (75 mg/day) |

|  |  |
| --- | --- |
| **RNPGx** | **Summary:** Testing for the main CYP2C19 deficiency alleles before instituting clopidogrel treatment is recommended (a test is essential for coronary angioplasty with stenting and based on the current state of knowledge this test is potentially useful in the other indications). For patients carrying at least one deficiency allele, the RNPGx recommends using an alternative treatment that is not a CYP2C19 substrate (eg. prasugrel, ticagrelor). |
|  | **Recommendation:** Although the presence of the CYP2C19\*17 allele, whether in a heterozygous or homozygous state, has been associated with increased hemorrhagic risk in patients treated with clopidogrel, it appears to favor the drug's efficacy, reducing the incidence of recurrent thromboembolic events. Consequently, considering current knowledge, there is no guideline concerning the appropriate dosage to prescribe for a patient with the \*17 allele. |

### **codeine**

**Gene**: CYP2D6    **Diplotype**: \*17/\*29    **Phenotype**: Intermediate Metabolizer

|  |  |
| --- | --- |
| **DPWG** | **Summary:** The Pharmacogenetics Working Group Guideline for codeine includes individual recommendations for cough or pain for CYP2D6 poor, intermediate, and ultrarapid metabolizer. In addition, for ultrarapid metabolizer, higher or lower doses and additional risk factors are taken into consideration. |
|  | **Recommendation:** For COUGH: 1. No action required. For PAIN: It is not possible to offer adequately substantiated advice for dose adjustment based on the limited available literature for this phenotype. 1. Be alert to a reduced effectiveness. 2. In the case of inadequate effectiveness: 1. Try a dose increase., 2. If this does not work: choose an alternative. Do not select tramadol, as this is also metabolised by CYP2D6. Morphine is not metabolised by CYP2D6. Oxycodone is metabolised by CYP2D6 to a limited extent, but this does not result in differences in analgesia in patients. 3. If no alternative is selected: advise the patient to report inadequate analgesia. |

|  |  |
| --- | --- |
| **CPNDS** | **Summary:** The Canadian Pharmacogenomics Network for Drug Safety (CPNDS) clinical recommendation group has published guidelines for the use of CYP2D6 genotype when prescribing codeine. They recommend that poor metabolizers of CYP2D6 should not receive codeine for pain relief, and ultrametabolizers of CYP2D6 should avoid codeine for pain relief. |
|  | **Recommendation:** In individuals with IM (intermediate metabolizer) or EM (extensive metabolizer) CYP2D6 genotypes, codeine can be used as per standard of care. Existing evidence suggests that caution is still warranted in CYP2D6 EMs receiving codeine if they are receiving maximal therapeutic doses of codeine and have additional risk factors for toxicity. |

|  |  |
| --- | --- |
| **CPIC** | **Summary:** Alternate non-tramadol analgesics are recommended for CYP2D6 ultrarapid and poor metabolizers. A label recommended age- or weight-specific dose of codeine is warranted for CYP2D6 normal and intermediate metabolizers. |
|  | **Recommendation:** Use codeine label recommended age- or weight-specific dosing. If no response and opioid use is warranted, consider a non-tramadol opioid |

### **desflurane**

**Gene**: CACNA1S    **Diplotype**: Reference/Reference    **Phenotype**: Uncertain Susceptibility

|  |  |
| --- | --- |
| **CPIC** | **Summary:** The CPIC Dosing Guideline recommends that halogenated volatile anesthetics such as desflurane, enflurane, halothane, isoflurane, methoxyflurane, sevoflurane and the depolarizing muscle relaxants succinylcholine are relatively contraindicated in persons with malignant hyperthermia susceptibility (MHS). See full guideline for disclaimers, further details and supporting evidence. |
|  | **Recommendation:** These results do not eliminate the chance that this patient is susceptible to Malignant Hyperthermia. The genetic cause of about half of all MH survivors, with MH susceptibility confirmed by contracture test, remains unknown [Article:28902675]. |

**Gene**: RYR1    **Diplotype**: Reference/Reference    **Phenotype**: Uncertain Susceptibility

|  |  |
| --- | --- |
| **CPIC** | **Summary:** The CPIC Dosing Guideline recommends that halogenated volatile anesthetics such as desflurane, enflurane, halothane, isoflurane, methoxyflurane, sevoflurane and the depolarizing muscle relaxants succinylcholine are relatively contraindicated in persons with malignant hyperthermia susceptibility (MHS). See full guideline for disclaimers, further details and supporting evidence. |
|  | **Recommendation:** These results do not eliminate the chance that this patient is susceptible to Malignant Hyperthermia. The genetic cause of about half of all MH survivors, with MH susceptibility confirmed by contracture test, remains unknown [Article:28902675]. |

### **desipramine**

**Gene**: CYP2D6    **Diplotype**: \*17/\*29    **Phenotype**: Intermediate Metabolizer

|  |  |
| --- | --- |
| **CPIC** | **Summary:** Tricyclic antidepressants have comparable pharmacokinetic properties, it may be reasonable to apply the CPIC Dosing Guideline for amitriptyline/nortriptyline and CYP2C19, CYP2D6 to other tricyclics including desipramine. The CPIC Dosing Guideline update for nortriptyline recommends a 25% dose reduction for CYP2D6 intermediate metabolizers. For CYP2D6 ultrarapid or poor metabolizers, an alternative drug should be considered. If nortriptyline is warranted, consider a 50% dose reduction in CYP2D6 poor metabolizers. |
|  | **Recommendation:** Consider 25% reduction of recommended starting dose. Utilize therapeutic drug monitoring to guide dose adjustments. |

### **dexlansoprazole**

**Gene**: CYP2C19    **Diplotype**: \*1/\*17    **Phenotype**: Rapid Metabolizer

|  |  |
| --- | --- |
| **CPIC** | **Summary:** The CPIC Dosing Guideline recommendations for dexlansoprazole are based on the similarity in its metabolism and lansoprazole and extrapolated from the first-generation PPIs. The guideline recommends to increase the starting daily dose and to monitor efficacy in CYP2C19 ultrarapid metabolizer. For CYP2C19 rapid and normal metabolizers in the treatment of H. pylori infection and erosive esophagitis increasing the dose might be considered after initiation with the standard starting daily dose. The recommendations for intermediate and poor metabolizers for chronic therapy (>12 weeks) and efficacy achieved is to consider 50% reduction in daily dose. See full guideline for further details and supporting evidence. |
|  | **Recommendation:** Initiate standard starting daily dose. Consider increasing dose by 50-100% for the treatment of H. pylori infection and erosive esophagitis. Daily dose may be given in divided doses. Monitor for efficacy. |

### **doxepin**

**Gene**: CYP2D6    **Diplotype**: \*17/\*29    **Phenotype**: Intermediate Metabolizer

|  |  |
| --- | --- |
| **CPIC** | **Summary:** Tricyclic antidepressants have comparable pharmacokinetic properties, it may be reasonable to apply the CPIC Dosing Guideline for amitriptyline and CYP2C19, CYP2D6 to other tricyclics including doxepin. The CPIC Dosing Guideline update for amitriptyline recommends an alternative drug for CYP2D6 ultrarapid or poor metabolizers and CYP2C19 ultrarapid, rapid or poor metabolizers. If amitriptyline is warranted, consider a 50% dose reduction in CYP2D6 or CYP2C19 poor metabolizers. For CYP2D6 intermediate metabolizers, a 25% dose reduction should be considered. |
|  | **Recommendation:** Consider 25% reduction of recommended starting dose. Utilize therapeutic drug monitoring to guide dose adjustments. |

|  |  |
| --- | --- |
| **DPWG** | **Summary:** The Dutch Pharmacogenetics Working Group Guideline for doxepin recommends dose changes and to monitor the effect and side effects or the plasma concentrations to set the maintenance dose for CYP2D6 poor (PM), intermediate (IM), and ultrarapid (UM) metabolizer or to avoid doxepin in UM. |
|  | **Recommendation:** Use 80% of the standard dose and monitor the effect and side effects or the plasma concentrations of doxepin and nordoxepin in order to set the maintenance dose. The therapeutic range is 100-250 ng/mL for the sum of doxepin and nordoxepin plasma concentrations. Values higher than 400 ng/mL are considered toxic. |

**Gene**: CYP2C19    **Diplotype**: \*1/\*17    **Phenotype**: Rapid Metabolizer

|  |  |
| --- | --- |
| **CPIC** | **Summary:** Tricyclic antidepressants have comparable pharmacokinetic properties, it may be reasonable to apply the CPIC Dosing Guideline for amitriptyline and CYP2C19, CYP2D6 to other tricyclics including doxepin. The CPIC Dosing Guideline update for amitriptyline recommends an alternative drug for CYP2D6 ultrarapid or poor metabolizers and CYP2C19 ultrarapid, rapid or poor metabolizers. If amitriptyline is warranted, consider a 50% dose reduction in CYP2D6 or CYP2C19 poor metabolizers. For CYP2D6 intermediate metabolizers, a 25% dose reduction should be considered. |
|  | **Recommendation:** Avoid tertiary amine use due to potential for sub-optimal response. Consider alternative drug not metabolized by CYP2C19. TCAs without major CYP2C19 metabolism include the secondary amines nortriptyline and desipramine. If a tertiary amine is warranted, utilize therapeutic drug monitoring to guide dose adjustments. |

### **efavirenz**

**Gene**: CYP2B6    **Diplotype**: \*1/\*18    **Phenotype**: Intermediate Metabolizer

|  |  |
| --- | --- |
| **CPIC** | **Summary:** Consider initiating efavirenz with a decreased dose of either 400 or 200 mg/day for patients who are CYP2B6 poor metabolizers. Consider initiating efavirenz with a decreased dose of 400 mg/day for patients who are CYP2B6 intermediate metabolizers. |
|  | **Recommendation:** Consider initiating efavirenz with decreased dose of 400 mg/day. |

|  |  |
| --- | --- |
| **DPWG** | **Summary:** Adjust the initial efavirenz dose for patients with the CYP2B6 PM phenotype along with the consideration of age, weight and BMI and titrate the dose to plasma concentration if needed. For patients with the \*5/\*6, \*5/\*18 or other CYP2B6 IM genotypes, determine the efavirenz plasma concentration if side effects occur and reduce the dose if needed. |
|  | **Recommendation:** 1. Determine the efavirenz plasma concentration if side effects occur and reduce the dose if needed. In 14 IM adults, a dose reduction to 400 mg/day (2/3rd of the standard dose) was sufficient to achieve therapeutic plasma concentrations and to reduce or resolve side effects. The therapeutic range established for efavirenz is 1000-4000 ng/ml. |

### **eliglustat**

**Gene**: CYP2D6    **Diplotype**: \*17/\*29    **Phenotype**: Intermediate Metabolizer

|  |  |
| --- | --- |
| **DPWG** | **Summary:** The Dutch Pharmacogenetics Working Group Guideline for eliglustat recommends to use an alternative in CYP2D6 ultrarapid metabolizer. For CYP2D6 poor metabolizer in combination with CYP3A inhibitors and strong inducers, the guideline recommends to choose an alternative if possible. For intermediate metabolizers recommendations are provided for co-medication with CYP2D6 and/or CYP3A inhibitors and CYP3A inducers. |
|  | **Recommendation:** - Co-medication with BOTH a MODERATE to STRONG CYP2D6 INHIBITOR AND a MODERATE to STRONG CYP3A INHIBITOR: Eliglustat is contra-indicated. 1. Choose an alternative if possible. Strong CYP2D6 inhibitor: for example paroxetine, fluoxetine, quinidine, bupropione. Moderate CYP2D6 inhibitor: for example duloxetine, terbinafine, moclobemide, mirabegron, cinacalcet, dronedarone. Strong CYP3A inhibitor: for example ketoconazole, clarithromycin, itraconazole, cobicistat, indinavir, lopinavir, ritonavir, saquinavir, telaprevir, tipranavir, posaconazole, voriconazole, telithromycin, conivaptan, boceprevir. Moderate CYP3A inhibitor: for example erythromycin, ciprofloxacin, fluconazole, diltiazem, verapamil, aprepitant, atazanavir, darunavir, fosamprenavir, imatinib, cimetidine. - Co-medication with a STRONG CYP2D6 INHIBITOR (e.g. paroxetine, fluoxetine, quinidine, bupropione): 1. Use a dose of 84mg eliglustat 1x daily. - Co-medication with a MODERATE CYP2D6 INHIBITOR (for example duloxetine, terbinafine, moclobemide, mirabegron, cinacalcet, dronedarone): 1. Consider a dose of 84mg eliglustat 1x daily. 2. Be alert to side effects. - Co-medication with a STRONG CYP3A INHIBITOR (for example ketoconazole, clarithromycin, itraconazole, cobicistat, indinavir, lopinavir, ritonavir, saquinavir, telaprevir, tipranavir, posaconazole, voriconazole, telithromycin, conivaptan, boceprevir): 1. Choose an alternative if possible. 2. If an alternative is not an option: consider a dose of 84 mg eliglustat 1x daily and be alert to side effects. - Co-medication with a MODERATE CYP3A INHIBITOR (for example erythromycin, ciprofloxacin, fluconazole, diltiazem, verapamil, aprepitant, atazanavir, darunavir, fosamprenavir, imatinib, cimetidine): 1. Choose an alternative. 2. If an alternative is not an option: consider a dose of 84mg eliglustat 1x daily and be alert to side effects. - Co-medication with a STRONG CYP3A INDUCER (for example rifampicin, carbamazepine, phenobarbital, phenytoin, rifabutine, hypericum): Eliglustat is not recommended. The plasma concentration may decrease so sharply that a therapeutic effect cannot be achieved. 1. Choose an alternative if possible. - NO co-medication with a moderate or strong CYP2D6 or CYP3A inhibitor or strong CYP3A inducer: 1. Use the standard dose of 84mg 2x daily. |

### **enflurane**

**Gene**: RYR1    **Diplotype**: Reference/Reference    **Phenotype**: Uncertain Susceptibility

|  |  |
| --- | --- |
| **CPIC** | **Summary:** The CPIC Dosing Guideline recommends that halogenated volatile anesthetics such as desflurane, enflurane, halothane, isoflurane, methoxyflurane, sevoflurane and the depolarizing muscle relaxants succinylcholine are relatively contraindicated in persons with malignant hyperthermia susceptibility (MHS). See full guideline for disclaimers, further details and supporting evidence. |
|  | **Recommendation:** These results do not eliminate the chance that this patient is susceptible to Malignant Hyperthermia. The genetic cause of about half of all MH survivors, with MH susceptibility confirmed by contracture test, remains unknown [Article:28902675]. |

**Gene**: CACNA1S    **Diplotype**: Reference/Reference    **Phenotype**: Uncertain Susceptibility

|  |  |
| --- | --- |
| **CPIC** | **Summary:** The CPIC Dosing Guideline recommends that halogenated volatile anesthetics such as desflurane, enflurane, halothane, isoflurane, methoxyflurane, sevoflurane and the depolarizing muscle relaxants succinylcholine are relatively contraindicated in persons with malignant hyperthermia susceptibility (MHS). See full guideline for disclaimers, further details and supporting evidence. |
|  | **Recommendation:** These results do not eliminate the chance that this patient is susceptible to Malignant Hyperthermia. The genetic cause of about half of all MH survivors, with MH susceptibility confirmed by contracture test, remains unknown [Article:28902675]. |

### **flecainide**

**Gene**: CYP2D6    **Diplotype**: \*17/\*29    **Phenotype**: Intermediate Metabolizer

|  |  |
| --- | --- |
| **DPWG** | **Summary:** Reduce flecainide dose by 50% for CYP2D6 poor metabolizer (PM) and record an ECG and monitor the plasma concentration. Reduce flecainide dose to 75% of the standard dose for CYP2D6 intermediate metabolizer (IM) patients with indications other than diagnosis of Brugada syndrome and record an ECG and monitor the plasma concentration. |
|  | **Recommendation:** 1. Indications other than diagnosis of Brugada syndrome: reduce the dose to 75% of the standard dose and record an ECG and monitor the plasma concentration.2.Provocation test for diagnosis of Brugada syndrome:No action required.At a dose of 2.0 mg/kg body weight to a maximum of 150 mg, the response is better for patients with alleles that result in reduced activity.All 5 patients with these alleles and 20% of the patients with two fully active alleles exhibited a response within 30 minutes. |

### **fluorouracil**

**Gene**: DPYD    **Diplotype**: c.85T>C (\*9A)/c.1349C>T    **Phenotype**: Normal Metabolizer

|  |  |
| --- | --- |
| **CPIC** | **Summary:** The CPIC Dosing Guideline for 5-fluorouracil and capecitabine recommends an alternative drug for patients who are DPYD poor metabolizers with an activity score of 0. In those who are poor metabolizers with an activity score of 0.5, an alternative drug is also recommended, but if this is not considered a suitable therapeutic option, 5-fluorouracil or capecitabine should be administered at a strongly reduced dose with early therapeutic drug monitoring. Patients who are intermediate metabolizers with an activity score of 1 or 1.5 should receive a dose reduction of 50%. Patients with the c.[2846A>T];[2846A>T] genotype may require a >50% dose reduction. |
|  | **Recommendation:** Based on genotype, there is no indication to change dose or therapy. Use label-recommended dosage and administration |

### **flurbiprofen**

**Gene**: CYP2C9    **Diplotype**: \*1/\*1    **Phenotype**: Normal Metabolizer

|  |  |
| --- | --- |
| **CPIC** | **Summary:** The CPIC Dosing Guideline for celecoxib, flurbiprofen, ibuprofen and lornoxicam recommends initiating therapy with 25-50% of the lowest recommended starting dose for CYP2C9 poor metabolizers and initiating therapy with lowest recommended starting dose for CYP2C9 intermediate metabolizers with activity score of 1. See full guideline for further details and supporting evidence. |
|  | **Recommendation:** Initiate therapy with recommended starting dose. In accordance with the prescribing information, use the lowest effective dosage for shortest duration consistent with individual patient treatment goals. |

### **fluvastatin**

**Gene**: SLCO1B1    **Diplotype**: \*20/\*30    **Phenotype**: Indeterminate

|  |  |
| --- | --- |
| **CPIC** | **Summary:** CYP2C9 IMs should avoid fluvastatin doses greater than 40mg while CYP2C9 PMs should avoid doses greater than 20mg. If higher doses are required for desired efficacy, an alternative statin should be considered. Patients with SLCO1B1 poor function should also avoid fluvastatin doses greater than 40mg and and adjust doses of fluvastatin based on disease-specific guidelines. Patients with both SLCO1B1 poor function and CYP2C9 IM/PM should be prescribed an alternative statin depending on the desired potency. |
|  | **Recommendation:** No recommendation. |

### **fluvoxamine**

**Gene**: CYP2D6    **Diplotype**: \*17/\*29    **Phenotype**: Intermediate Metabolizer

|  |  |
| --- | --- |
| **CPIC** | **Summary:** The CPIC Dosing Guideline for the selective serotonin reuptake inhibitor fluvoxamine recommends to consider a 25-50% reduction of recommended starting dose and titrate to response or use an alternative drug not metabolized by CYP2D6 for CYP2D6 poor metabolizers. |
|  | **Recommendation:** Initiate therapy with recommended starting dose. |

### **fosphenytoin**

**Gene**: CYP2C9    **Diplotype**: \*1/\*1    **Phenotype**: Normal Metabolizer

|  |  |
| --- | --- |
| **CPIC** | **Summary:** Phenytoin/fosphenytoin is contraindicated in individuals with the HLA-B\*15:02 variant allele ("HLA-B\*15:02-positive") due to significantly increased risk of phenytoin-induced cutaneous adverse reactions of Stevens-Johnson syndrome (SJS) and toxic epidermal necrolysis (TEN). Additionally, patients with the CYP2C9 poor metabolizer phenotype or with a CYP2C9 activity score of 1 may require reduced doses of phenytoin/fosphenytoin. |
|  | **Recommendation:** No adjustments needed from typical dosing strategies. Subsequent doses should be adjusted according to therapeutic drug monitoring, response, and side effects. An HLA-B\*15:02 negative test does not eliminate the risk of phenytoin-induced SJS/TEN, and patients should be carefully monitored according to standard practice. |

### **haloperidol**

**Gene**: CYP2D6    **Diplotype**: \*17/\*29    **Phenotype**: Intermediate Metabolizer

|  |  |
| --- | --- |
| **DPWG** | **Summary:** The recommendation for CYP2D6 poor metabolizers is to use 60% of the standard dose of haloperidol and for CYP2D6 ultrarapid metabolizers to use 1.5 times the standard dose or to choose an alternative to haloperidol. |
|  | **Recommendation:** NO action is required for this gene-drug interaction. |

### **halothane**

**Gene**: CACNA1S    **Diplotype**: Reference/Reference    **Phenotype**: Uncertain Susceptibility

|  |  |
| --- | --- |
| **CPIC** | **Summary:** The CPIC Dosing Guideline recommends that halogenated volatile anesthetics such as desflurane, enflurane, halothane, isoflurane, methoxyflurane, sevoflurane and the depolarizing muscle relaxants succinylcholine are relatively contraindicated in persons with malignant hyperthermia susceptibility (MHS). See full guideline for disclaimers, further details and supporting evidence. |
|  | **Recommendation:** These results do not eliminate the chance that this patient is susceptible to Malignant Hyperthermia. The genetic cause of about half of all MH survivors, with MH susceptibility confirmed by contracture test, remains unknown [Article:28902675]. |

**Gene**: RYR1    **Diplotype**: Reference/Reference    **Phenotype**: Uncertain Susceptibility

|  |  |
| --- | --- |
| **CPIC** | **Summary:** The CPIC Dosing Guideline recommends that halogenated volatile anesthetics such as desflurane, enflurane, halothane, isoflurane, methoxyflurane, sevoflurane and the depolarizing muscle relaxants succinylcholine are relatively contraindicated in persons with malignant hyperthermia susceptibility (MHS). See full guideline for disclaimers, further details and supporting evidence. |
|  | **Recommendation:** These results do not eliminate the chance that this patient is susceptible to Malignant Hyperthermia. The genetic cause of about half of all MH survivors, with MH susceptibility confirmed by contracture test, remains unknown [Article:28902675]. |

### **hydrocodone**

**Gene**: CYP2D6    **Diplotype**: \*17/\*29    **Phenotype**: Intermediate Metabolizer

|  |  |
| --- | --- |
| **CPIC** | **Summary:** CYP2D6 intermediate and poor metabolizers should initiate hydrocodone therapy using the label recommended age- or weight-specific dosing. However, if there is no response to hydrocodone in these patients, an alternative analgesic should be considered. |
|  | **Recommendation:** Use hydrocodone label recommended age- or weight-specific dosing. If no response and opioid use is warranted, consider non-codeine or non-tramadol opioid |

### **ibuprofen**

**Gene**: CYP2C9    **Diplotype**: \*1/\*1    **Phenotype**: Normal Metabolizer

|  |  |
| --- | --- |
| **CPIC** | **Summary:** The CPIC Dosing Guideline for celecoxib, flurbiprofen, ibuprofen and lornoxicam recommends initiating therapy with 25-50% of the lowest recommended starting dose for CYP2C9 poor metabolizers and initiating therapy with lowest recommended starting dose for CYP2C9 intermediate metabolizers with activity score of 1. See full guideline for further details and supporting evidence. |
|  | **Recommendation:** Initiate therapy with recommended starting dose. In accordance with the prescribing information, use the lowest effective dosage for shortest duration consistent with individual patient treatment goals. |

### **imipramine**

**Gene**: CYP2C19    **Diplotype**: \*1/\*17    **Phenotype**: Rapid Metabolizer

|  |  |
| --- | --- |
| **CPIC** | **Summary:** Tricyclic antidepressants have comparable pharmacokinetic properties, it may be reasonable to apply the CPIC Dosing Guideline for amitriptyline and CYP2C19, CYP2D6 to other tricyclics including imipramine. The CPIC Dosing Guideline update for amitriptyline recommends an alternative drug for CYP2D6 ultrarapid or poor metabolizers and CYP2C19 ultrarapid, rapid or poor metabolizers. If amitriptyline is warranted, consider a 50% dose reduction in CYP2D6 or CYP2C19 poor metabolizers. For CYP2D6 intermediate metabolizers, a 25% dose reduction should be considered. |
|  | **Recommendation:** Avoid tertiary amine use due to potential for sub-optimal response. Consider alternative drug not metabolized by CYP2C19. TCAs without major CYP2C19 metabolism include the secondary amines nortriptyline and desipramine. If a tertiary amine is warranted, utilize therapeutic drug monitoring to guide dose adjustments. |

**Gene**: CYP2D6    **Diplotype**: \*17/\*29    **Phenotype**: Intermediate Metabolizer

|  |  |
| --- | --- |
| **CPIC** | **Summary:** Tricyclic antidepressants have comparable pharmacokinetic properties, it may be reasonable to apply the CPIC Dosing Guideline for amitriptyline and CYP2C19, CYP2D6 to other tricyclics including imipramine. The CPIC Dosing Guideline update for amitriptyline recommends an alternative drug for CYP2D6 ultrarapid or poor metabolizers and CYP2C19 ultrarapid, rapid or poor metabolizers. If amitriptyline is warranted, consider a 50% dose reduction in CYP2D6 or CYP2C19 poor metabolizers. For CYP2D6 intermediate metabolizers, a 25% dose reduction should be considered. |
|  | **Recommendation:** Consider 25% reduction of recommended starting dose. Utilize therapeutic drug monitoring to guide dose adjustments. |

|  |  |
| --- | --- |
| **DPWG** | **Summary:** CYP2D6 poor metabolizers should receive 30% of the standard dose of imipramine, CYP2D6 intermediate metabolizers should receive 70% of the standard dose, and CYP2D6 ultra-rapid metabolizers should receive 1.7 times the standard dose. Patients should be monitored for the effect and side effects or the plasma concentrations of imipramine and desipramine in order to set the maintenance dose. |
|  | **Recommendation:** Use 70% of the standard dose and monitor the effect and side effects or the plasma concentrations of imipramine and desipramine in order to set the maintenance dose. The therapeutic range is 150-300 ng/mL for the sum of the imipramine and desipramine plasma concentrations. Values exceeding 500 ng/mL are considered toxic. |

### **irinotecan**

**Gene**: UGT1A1    **Diplotype**: \*80+\*28/\*80+\*28    **Phenotype**: Poor Metabolizer

|  |  |
| --- | --- |
| **DPWG** | **Summary:** Dose reductions are recommended for irinotecan for patients who are UGT1A1 \*28/\*28 or UGT1A1 PM, starting with 70% of starting dose and increasing as tolerated, guided by neutrophil count. |
|  | **Recommendation:** Start with 70% of the standard dose If the patient tolerates this initial dose, the dose can be increased, guided by the neutrophil count. |

### **isoflurane**

**Gene**: CACNA1S    **Diplotype**: Reference/Reference    **Phenotype**: Uncertain Susceptibility

|  |  |
| --- | --- |
| **CPIC** | **Summary:** The CPIC Dosing Guideline recommends that halogenated volatile anesthetics such as desflurane, enflurane, halothane, isoflurane, methoxyflurane, sevoflurane and the depolarizing muscle relaxants succinylcholine are relatively contraindicated in persons with malignant hyperthermia susceptibility (MHS). See full guideline for disclaimers, further details and supporting evidence. |
|  | **Recommendation:** These results do not eliminate the chance that this patient is susceptible to Malignant Hyperthermia. The genetic cause of about half of all MH survivors, with MH susceptibility confirmed by contracture test, remains unknown [Article:28902675]. |

**Gene**: RYR1    **Diplotype**: Reference/Reference    **Phenotype**: Uncertain Susceptibility

|  |  |
| --- | --- |
| **CPIC** | **Summary:** The CPIC Dosing Guideline recommends that halogenated volatile anesthetics such as desflurane, enflurane, halothane, isoflurane, methoxyflurane, sevoflurane and the depolarizing muscle relaxants succinylcholine are relatively contraindicated in persons with malignant hyperthermia susceptibility (MHS). See full guideline for disclaimers, further details and supporting evidence. |
|  | **Recommendation:** These results do not eliminate the chance that this patient is susceptible to Malignant Hyperthermia. The genetic cause of about half of all MH survivors, with MH susceptibility confirmed by contracture test, remains unknown [Article:28902675]. |

### **lansoprazole**

**Gene**: CYP2C19    **Diplotype**: \*1/\*17    **Phenotype**: Rapid Metabolizer

|  |  |
| --- | --- |
| **CPIC** | **Summary:** The CPIC Dosing Guideline for omeprazole, lansoprazole, pantoprazole recommends to increase the starting daily dose and to monitor efficacy in CYP2C19 ultrarapid metabolizer. For CYP2C19 rapid and normal metabolizers in the treatment of H. pylori infection and erosive esophagitis increasing the dose might be considered after initiation with the standard starting daily dose. The recommendations for intermediate and poor metabolizer for chronic therapy (>12 weeks) and efficacy achieved is to consider 50% reduction in daily dose. See full guideline for further details and supporting evidence. |
|  | **Recommendation:** Initiate standard starting daily dose. Consider increasing dose by 50-100% for the treatment of H. pylori infection and erosive esophagitis. Daily dose may be given in divided doses. Monitor for efficacy. |

### **lornoxicam**

**Gene**: CYP2C9    **Diplotype**: \*1/\*1    **Phenotype**: Normal Metabolizer

|  |  |
| --- | --- |
| **CPIC** | **Summary:** The CPIC Dosing Guideline for celecoxib, flurbiprofen, ibuprofen and lornoxicam recommends initiating therapy with 25-50% of the lowest recommended starting dose for CYP2C9 poor metabolizers and initiating therapy with lowest recommended starting dose for CYP2C9 intermediate metabolizers with activity score of 1. See full guideline for further details and supporting evidence. |
|  | **Recommendation:** Initiate therapy with recommended starting dose. In accordance with the prescribing information, use the lowest effective dosage for shortest duration consistent with individual patient treatment goals. |

### **lovastatin**

**Gene**: SLCO1B1    **Diplotype**: \*20/\*30    **Phenotype**: Indeterminate

|  |  |
| --- | --- |
| **CPIC** | **Summary:** Prescribe an alternative statin depending on the desired potency for patients with SLCO1B1 decreased function, possible decreased function or poor function phenotype. If lovastatin therapy is warranted in patients with SLCO1B1 decreased or possible decreased phenotype, limit dose to <20mg/day. |
|  | **Recommendation:** No recommendation. |

### **meloxicam**

**Gene**: CYP2C9    **Diplotype**: \*1/\*1    **Phenotype**: Normal Metabolizer

|  |  |
| --- | --- |
| **CPIC** | **Summary:** The CPIC Dosing Guideline for meloxicam recommends alternative therapy for CYP2C9 poor metabolizers due to markedly prolonged half-life, and initiating therapy with 50% of the lowest recommended starting dose or choose an alternative therapy for CYP2C9 intermediate metabolizers with activity score of 1. See full guideline for further details and supporting evidence. |
|  | **Recommendation:** Initiate therapy with recommended starting dose. In accordance with the prescribing information, use the lowest effective dosage for shortest duration consistent with individual patient treatment goals. |

### **mercaptopurine**

**Gene**: NUDT15    **Diplotype**: \*1/\*1    **Phenotype**: Normal Metabolizer

|  |  |
| --- | --- |
| **CPIC** | **Summary:** Consider an alternate agent or extreme dose reduction of mercaptopurine for patients who are TPMT or NUDT15 poor metabolizers. Start at 30-80% of target dose for patients who are TPMT or NUDT15 intermediate metabolizers. |
|  | **Recommendation:** Start with normal starting dose (e.g., 75 mg/m2/day or 1.5 mg/kg/day) and adjust doses of mercaptopurine (and of any other myelosuppressive therapy) without any special emphasis on mercaptopurine compared to other agents. Allow at least 2 weeks to reach steady-state after each dose adjustment. |

**Gene**: TPMT    **Diplotype**: \*1/\*1    **Phenotype**: Normal Metabolizer

|  |  |
| --- | --- |
| **CPIC** | **Summary:** Consider an alternate agent or extreme dose reduction of mercaptopurine for patients who are TPMT or NUDT15 poor metabolizers. Start at 30-80% of target dose for patients who are TPMT or NUDT15 intermediate metabolizers. |
|  | **Recommendation:** Start with normal starting dose (e.g., 75 mg/m2/day or 1.5 mg/kg/day) and adjust doses of mercaptopurine (and of any other myelosuppressive therapy) without any special emphasis on mercaptopurine compared to other agents. Allow at least 2 weeks to reach steady-state after each dose adjustment. |

### **methoxyflurane**

**Gene**: RYR1    **Diplotype**: Reference/Reference    **Phenotype**: Uncertain Susceptibility

|  |  |
| --- | --- |
| **CPIC** | **Summary:** The CPIC Dosing Guideline recommends that halogenated volatile anesthetics such as desflurane, enflurane, halothane, isoflurane, methoxyflurane, sevoflurane and the depolarizing muscle relaxants succinylcholine are relatively contraindicated in persons with malignant hyperthermia susceptibility (MHS). See full guideline for disclaimers, further details and supporting evidence. |
|  | **Recommendation:** These results do not eliminate the chance that this patient is susceptible to Malignant Hyperthermia. The genetic cause of about half of all MH survivors, with MH susceptibility confirmed by contracture test, remains unknown [Article:28902675]. |

**Gene**: CACNA1S    **Diplotype**: Reference/Reference    **Phenotype**: Uncertain Susceptibility

|  |  |
| --- | --- |
| **CPIC** | **Summary:** The CPIC Dosing Guideline recommends that halogenated volatile anesthetics such as desflurane, enflurane, halothane, isoflurane, methoxyflurane, sevoflurane and the depolarizing muscle relaxants succinylcholine are relatively contraindicated in persons with malignant hyperthermia susceptibility (MHS). See full guideline for disclaimers, further details and supporting evidence. |
|  | **Recommendation:** These results do not eliminate the chance that this patient is susceptible to Malignant Hyperthermia. The genetic cause of about half of all MH survivors, with MH susceptibility confirmed by contracture test, remains unknown [Article:28902675]. |

### **metoprolol**

**Gene**: CYP2D6    **Diplotype**: \*17/\*29    **Phenotype**: Intermediate Metabolizer

|  |  |
| --- | --- |
| **DPWG** | **Summary:** For CYP2D6 poor and intermediate metabolizer patients, if a GRADUAL REDUCTION in HEART RATE is desired, or in the event of SYMPTOMATIC BRADYCARDIA, use smaller steps in dose titration and/or prescribe no more than 25% or 50% of the standard dose, respectively. For CYP2D6 ultra metabolizers, use the maximum dose for the relevant indication as a target dose, and if the effectiveness is still insufficient: increase the dose based on effectiveness and side effects to 2.5 times the standard dose or select an alternative drug. |
|  | **Recommendation:** If a GRADUAL REDUCTION in HEART RATE is desired, or in the event of SYMPTOMATIC BRADYCARDIA: 1. use smaller steps in dose titration and/or prescribe no more than 50% of the standard dose. OTHER CASES: 1. no action required |

### **nortriptyline**

**Gene**: CYP2D6    **Diplotype**: \*17/\*29    **Phenotype**: Intermediate Metabolizer

|  |  |
| --- | --- |
| **CPIC** | **Summary:** The CPIC Dosing Guideline update for nortriptyline recommends a 25% dose reduction for CYP2D6 intermediate metabolizers. For CYP2D6 ultrarapid or poor metabolizers, an alternative drug should be considered. If nortriptyline is warranted, consider a 50% dose reduction in CYP2D6 poor metabolizers. |
|  | **Recommendation:** Consider 25% reduction of recommended starting dose. Utilize therapeutic drug monitoring to guide dose adjustments. |

|  |  |
| --- | --- |
| **DPWG** | **Summary:** The Dutch Pharmacogenetics Working Group Guideline for nortriptyline recommends a dose reduction for CYP2D6 poor or intermediate metabolizer patients. For CYP2D6 ultrarapid metabolizers, select an alternative drug or use 1.7 times the standard dose. Monitoring of nortriptyline and 10-hydroxynortriptyline plasma concentrations is recommended. |
|  | **Recommendation:** Use 60% of the standard dose and monitor the effect and side effects or the plasma concentration of nortriptyline in order to set the maintenance dose. The therapeutic range of nortriptyline is 50-150 ng/mL. Values exceeding 250 ng/mL are considered toxic. |

### **omeprazole**

**Gene**: CYP2C19    **Diplotype**: \*1/\*17    **Phenotype**: Rapid Metabolizer

|  |  |
| --- | --- |
| **CPIC** | **Summary:** The CPIC Dosing Guideline for omeprazole, lansoprazole, pantoprazole recommends to increase the starting daily dose and to monitor efficacy in CYP2C19 ultrarapid metabolizer. For CYP2C19 rapid and normal metabolizers in the treatment of H. pylori infection and erosive esophagitis increasing the dose might be considered after initiation with the standard starting daily dose. The recommendations for intermediate and poor metabolizer for chronic therapy (>12 weeks) and efficacy achieved is to consider 50% reduction in daily dose. See full guideline for further details and supporting evidence. |
|  | **Recommendation:** Initiate standard starting daily dose. Consider increasing dose by 50-100% for the treatment of H. pylori infection and erosive esophagitis. Daily dose may be given in divided doses. Monitor for efficacy. |

### **ondansetron**

**Gene**: CYP2D6    **Diplotype**: \*17/\*29    **Phenotype**: Intermediate Metabolizer

|  |  |
| --- | --- |
| **CPIC** | **Summary:** The CPIC dosing guideline for ondansetron recommends selecting an alternate drug for CYP2D6 ultrarapid metabolizers. It is recommended that the alternate drug not be predominantly metabolized by CYP2D6 (eg. granisetron). |
|  | **Recommendation:** Insufficient evidence demonstrating clinical impact based on CYP2D6 genotype. Initiate therapy with recommended starting dose. |

### **pantoprazole**

**Gene**: CYP2C19    **Diplotype**: \*1/\*17    **Phenotype**: Rapid Metabolizer

|  |  |
| --- | --- |
| **CPIC** | **Summary:** The CPIC Dosing Guideline for omeprazole, lansoprazole, pantoprazole recommends to increase the starting daily dose and to monitor efficacy in CYP2C19 ultrarapid metabolizer. For CYP2C19 rapid and normal metabolizers in the treatment of H. pylori infection and erosive esophagitis increasing the dose might be considered after initiation with the standard starting daily dose. The recommendations for intermediate and poor metabolizer for chronic therapy (>12 weeks) and efficacy achieved is to consider 50% reduction in daily dose. See full guideline for further details and supporting evidence. |
|  | **Recommendation:** Initiate standard starting daily dose. Consider increasing dose by 50-100% for the treatment of H. pylori infection and erosive esophagitis. Daily dose may be given in divided doses. Monitor for efficacy. |

### **paroxetine**

**Gene**: CYP2D6    **Diplotype**: \*17/\*29    **Phenotype**: Intermediate Metabolizer

|  |  |
| --- | --- |
| **CPIC** | **Summary:** The CPIC Dosing Guideline for the selective serotonin reuptake inhibitor paroxetine recommends an alternative drug not predominantly metabolized by CYP2D6 for CYP2D6 ultrarapid metabolizers and for CYP2D6 poor metabolizers. For CYP2D6 poor metabolizers, if paroxetine use is warranted, consider a 50% reduction of recommended starting dose and titrate to response. |
|  | **Recommendation:** Initiate therapy with recommended starting dose. |

|  |  |
| --- | --- |
| **DPWG** | **Summary:** Select an alternative drug rather than paroxetine for CYP2D6 ultrarapid metabolizer patients. |
|  | **Recommendation:** NO action is needed for this gene-drug interaction. |

### **peginterferon alfa-2a**

**Gene**: IFNL3    **Diplotype**: T/T    **Phenotype**: -

|  |  |
| --- | --- |
| **CPIC** | **Summary:** IFNL3 (IL28B) variation (rs12979860) is the strongest baseline predictor of response to PEG-interferon-alpha-containing regimens in HCV genotype 1 patients. Patients with the favorable response genotype (rs12979860 CC) have increased likelihood of response (higher SVR rate) to PEG-interferon-alpha-containing regimens as compared to patients with unfavorable response genotype (rs12979860 CT or TT). Consider implications before initiating PEG-IFN alpha and RBV containing regimens. |
|  | **Recommendation:** Implications for PEG-IFN alpha and RBV: Approximately 30% chance for SVR after 48 weeks of treatment. Consider implications before initiating PEG-IFN alpha and RBV containing regimens. Implications for protease inhibitor combinations with PEG-IFN alpha and RBV therapy: Approximately 60% chance for SVR after 24-48 weeks of treatment. Approximately 50% of patients are eligible for shortened therapy (24-28 weeks). Consider implications before initiating PEG-IFN and RBV containing regimens. |

### **peginterferon alfa-2b**

**Gene**: IFNL3    **Diplotype**: T/T    **Phenotype**: -

|  |  |
| --- | --- |
| **CPIC** | **Summary:** IFNL3 (IL28B) variation (rs12979860) is the strongest baseline predictor of response to PEG-interferon-alpha-containing regimens in HCV genotype 1 patients. Patients with the favorable response genotype (rs12979860 CC) have increased likelihood of response (higher SVR rate) to PEG-interferon-alpha-containing regimens as compared to patients with unfavorable response genotype (rs12979860 CT or TT). Consider implications before initiating PEG-IFN alpha and RBV containing regimens. |
|  | **Recommendation:** Implications for PEG-IFN alpha and RBV: Approximately 30% chance for SVR after 48 weeks of treatment. Consider implications before initiating PEG-IFN alpha and RBV containing regimens. Implications for protease inhibitor combinations with PEG-IFN alpha and RBV therapy: Approximately 60% chance for SVR after 24-48 weeks of treatment. Approximately 50% of patients are eligible for shortened therapy (24-28 weeks). Consider implications before initiating PEG-IFN and RBV containing regimens. |

### **phenytoin**

**Gene**: CYP2C9    **Diplotype**: \*1/\*1    **Phenotype**: Normal Metabolizer

|  |  |
| --- | --- |
| **CPIC** | **Summary:** Phenytoin/fosphenytoin is contraindicated in individuals with the HLA-B\*15:02 variant allele ("HLA-B\*15:02-positive") due to significantly increased risk of phenytoin-induced cutaneous adverse reactions of Stevens-Johnson syndrome (SJS) and toxic epidermal necrolysis (TEN). Additionally, patients with the CYP2C9 poor metabolizer phenotype or with a CYP2C9 activity score of 1 may require reduced doses of phenytoin/fosphenytoin. |
|  | **Recommendation:** No adjustments needed from typical dosing strategies. Subsequent doses should be adjusted according to therapeutic drug monitoring, response, and side effects. An HLA-B\*15:02 negative test does not eliminate the risk of phenytoin-induced SJS/TEN, and patients should be carefully monitored according to standard practice. |

### **pimozide**

**Gene**: CYP2D6    **Diplotype**: \*17/\*29    **Phenotype**: Intermediate Metabolizer

|  |  |
| --- | --- |
| **DPWG** | **Summary:** Patients who are CYP2D6 intermediate metabolizers should be given no more than 80% of the standard maximum dose of pimozide while patients who are CYP2D6 poor metabolizers should be given no more than 50% of the standard maximum dose. |
|  | **Recommendation:** Use no more than the following doses (80% of the standard maximum dose): 12 years and older: 16 mg/day younger than 12 years: 0.08 mg/kg per day to a maximum of 3 mg/day |

### **piroxicam**

**Gene**: CYP2C9    **Diplotype**: \*1/\*1    **Phenotype**: Normal Metabolizer

|  |  |
| --- | --- |
| **CPIC** | **Summary:** The CPIC Dosing Guideline for piroxicam recommends that CYP2C9 poor metabolizers and intermediate metabolizers with activity score of 1 should choose an alternative therapy not metabolized by CYP2C9 or not significantly impacted by CYP2C9 genetic variants in vivo or choose an NSAID metabolized by CYP2C9 but with a shorter half-life. See full guideline for further details and supporting evidence. |
|  | **Recommendation:** Initiate therapy with recommended starting dose. In accordance with the prescribing information, use the lowest effective dosage for shortest duration consistent with individual patient treatment goals. |

### **pitavastatin**

**Gene**: SLCO1B1    **Diplotype**: \*20/\*30    **Phenotype**: Indeterminate

|  |  |
| --- | --- |
| **CPIC** | **Summary:** Prescribe ≤1mg as a starting dose for patients with SLCO1B1 poor function phenotype. Prescribe ≤2mg as a starting dose for patients with SLCO1B1 decreased or possible decreased phenotype. Adjust doses of pitavastatin based on disease-specific guidelines. Consider an alternative statin or combination therapy if higher doses are needed. |
|  | **Recommendation:** No recommendation. |

### **pravastatin**

**Gene**: SLCO1B1    **Diplotype**: \*20/\*30    **Phenotype**: Indeterminate

|  |  |
| --- | --- |
| **CPIC** | **Summary:** Prescribe ≤40mg as a starting dose and adjust doses of pravastatin based on disease-specific guidelines for patients with SLCO1B1 poor function phenotype. Prescribe desired starting dose and adjust doses of pravastatin based on disease-specific guidelines for patients with SLCO1B1 decreased or possible decreased phenotype. Prescriber should be aware of possible increased risk for myopathy with pravastatin especially with doses >40mg per day. |
|  | **Recommendation:** No recommendation. |

### **propafenone**

**Gene**: CYP2D6    **Diplotype**: \*17/\*29    **Phenotype**: Intermediate Metabolizer

|  |  |
| --- | --- |
| **DPWG** | **Summary:** Reduce the dose of propafenone by 70% for CYP2D6 poor metabolizers, and monitor propafenone plasma concentrations or use an alternative drug for CYP2D6 intermediate and ultrarapid metabolizers. |
|  | **Recommendation:** It is not possible to offer adequately substantiated recommendations for dose adjustment based on the literature. 1. Either guide the dose by therapeutic drug monitoring, perform an ECG and be alert to side effects 2. Or choose an alternative. Antiarrhythmic drugs that are hardly if at all metabolised by CYP2D6 include, for example, sotalol, disopyramide, quinidine and amiodarone. |

### **rasburicase**

**Gene**: G6PD    **Diplotype**: A- 202A\_376G/A- 202A\_376G    **Phenotype**: Deficient

|  |  |
| --- | --- |
| **CPIC** | **Summary:** Rasburicase is contraindicated in G6PD deficient patients with or without chronic non-spherocytic hemolytic anemia (CNSHA). In patients with a negative or inconclusive genetic test result an enzyme activity test is recommended prior to rasburicase treatment to determine whether a patient is G6PD deficient. The G6PD gene is X-linked and therefore males only have one copy, whereas females have two copies. See full guideline for disclaimers, further details and supporting evidence. |
|  | **Recommendation:** Rasburicase is contraindicated; alternatives include allopurinol. |

### **ribavirin**

**Gene**: IFNL3    **Diplotype**: T/T    **Phenotype**: -

|  |  |
| --- | --- |
| **CPIC** | **Summary:** IFNL3 (IL28B) variation (rs12979860) is the strongest baseline predictor of response to PEG-interferon-alpha-containing regimens in HCV genotype 1 patients. Patients with the favorable response genotype (rs12979860 CC) have increased likelihood of response (higher SVR rate) to PEG-interferon-alpha-containing regimens as compared to patients with unfavorable response genotype (rs12979860 CT or TT). Consider implications before initiating PEG-IFN alpha and RBV containing regimens. |
|  | **Recommendation:** Implications for PEG-IFN alpha and RBV: Approximately 30% chance for SVR after 48 weeks of treatment. Consider implications before initiating PEG-IFN alpha and RBV containing regimens. Implications for protease inhibitor combinations with PEG-IFN alpha and RBV therapy: Approximately 60% chance for SVR after 24-48 weeks of treatment. Approximately 50% of patients are eligible for shortened therapy (24-28 weeks). Consider implications before initiating PEG-IFN and RBV containing regimens. |

### **risperidone**

**Gene**: CYP2D6    **Diplotype**: \*17/\*29    **Phenotype**: Intermediate Metabolizer

|  |  |
| --- | --- |
| **DPWG** | **Summary:** The Dutch Pharmacogenetics Working Group Guideline for risperidone recommends decreasing the dose for CYP2D6 poor metabolizers and using an alternative drug or titrate the dose according to the maximum dose for the active metabolite for CYP2D6 ultrarapid metabolizers. |
|  | **Recommendation:** NO action is needed for this gene-drug interaction. |

### **rosuvastatin**

**Gene**: SLCO1B1    **Diplotype**: \*20/\*30    **Phenotype**: Indeterminate

|  |  |
| --- | --- |
| **CPIC** | **Summary:** Prescribe ≤20mg as a starting dose and adjust doses of rosuvastatin based on disease-specific and specific population guidelines for patients who are SLCO1B1 or ABCG2 poor function phenotype. If dose >20mg needed for desired efficacy, consider combination therapy (i.e. rosuvastatin plus non-statin guideline directed medical therapy). Patients with both ABCG2 poor function and SLCO1B1 poor/decreased function should be prescribed ≤10mg as a starting dose. |
|  | **Recommendation:** No recommendation. |

### **sevoflurane**

**Gene**: RYR1    **Diplotype**: Reference/Reference    **Phenotype**: Uncertain Susceptibility

|  |  |
| --- | --- |
| **CPIC** | **Summary:** The CPIC Dosing Guideline recommends that halogenated volatile anesthetics such as desflurane, enflurane, halothane, isoflurane, methoxyflurane, sevoflurane and the depolarizing muscle relaxants succinylcholine are relatively contraindicated in persons with malignant hyperthermia susceptibility (MHS). See full guideline for disclaimers, further details and supporting evidence. |
|  | **Recommendation:** These results do not eliminate the chance that this patient is susceptible to Malignant Hyperthermia. The genetic cause of about half of all MH survivors, with MH susceptibility confirmed by contracture test, remains unknown [Article:28902675]. |

**Gene**: CACNA1S    **Diplotype**: Reference/Reference    **Phenotype**: Uncertain Susceptibility

|  |  |
| --- | --- |
| **CPIC** | **Summary:** The CPIC Dosing Guideline recommends that halogenated volatile anesthetics such as desflurane, enflurane, halothane, isoflurane, methoxyflurane, sevoflurane and the depolarizing muscle relaxants succinylcholine are relatively contraindicated in persons with malignant hyperthermia susceptibility (MHS). See full guideline for disclaimers, further details and supporting evidence. |
|  | **Recommendation:** These results do not eliminate the chance that this patient is susceptible to Malignant Hyperthermia. The genetic cause of about half of all MH survivors, with MH susceptibility confirmed by contracture test, remains unknown [Article:28902675]. |

### **simvastatin**

**Gene**: SLCO1B1    **Diplotype**: \*20/\*30    **Phenotype**: Indeterminate

|  |  |
| --- | --- |
| **CPIC** | **Summary:** Prescribe an alternative statin depending on the desired potency for patients with SLCO1B1 decreased function, possible decreased function or poor function phenotype. If simvastatin therapy is warranted in patients with SLCO1B1 decreased or possible decreased phenotype, limit dose to <20mg/day. |
|  | **Recommendation:** No recommendation. |

### **succinylcholine**

**Gene**: RYR1    **Diplotype**: Reference/Reference    **Phenotype**: Uncertain Susceptibility

|  |  |
| --- | --- |
| **CPIC** | **Summary:** The CPIC Dosing Guideline recommends that halogenated volatile anesthetics such as desflurane, enflurane, halothane, isoflurane, methoxyflurane, sevoflurane and the depolarizing muscle relaxants succinylcholine are relatively contraindicated in persons with malignant hyperthermia susceptibility (MHS). See full guideline for disclaimers, further details and supporting evidence. |
|  | **Recommendation:** These results do not eliminate the chance that this patient is susceptible to Malignant Hyperthermia. The genetic cause of about half of all MH survivors, with MH susceptibility confirmed by contracture test, remains unknown [Article:28902675]. |

**Gene**: CACNA1S    **Diplotype**: Reference/Reference    **Phenotype**: Uncertain Susceptibility

|  |  |
| --- | --- |
| **CPIC** | **Summary:** The CPIC Dosing Guideline recommends that halogenated volatile anesthetics such as desflurane, enflurane, halothane, isoflurane, methoxyflurane, sevoflurane and the depolarizing muscle relaxants succinylcholine are relatively contraindicated in persons with malignant hyperthermia susceptibility (MHS). See full guideline for disclaimers, further details and supporting evidence. |
|  | **Recommendation:** These results do not eliminate the chance that this patient is susceptible to Malignant Hyperthermia. The genetic cause of about half of all MH survivors, with MH susceptibility confirmed by contracture test, remains unknown [Article:28902675]. |

### **tacrolimus**

**Gene**: CYP3A5    **Diplotype**: \*1/\*3    **Phenotype**: Intermediate Metabolizer

|  |  |
| --- | --- |
| **RNPGx** | **Summary:** Testing for the CYP3A5\*3 and CYP3A4\*22 alleles is recommended in patients receiving a kidney, heart or lung transplant. CYP3A extensive or intermediate meatbolizers (EMs or IMs) should be given an initial dose of tacrolimus 1.5-2 times higher than that recommended for CYP3A poor metabolizers (PMs), up to a maximum dose of 0.30 mg/kg/day. |
|  | **Recommendation:** Based on TDM, 1.5 to 2 times the dose recommended to non-expressors; maximum dose 0.30mg/kg/day. |

|  |  |
| --- | --- |
| **CPIC** | **Summary:** The CPIC dosing guideline for tacrolimus recommends increasing the starting dose by 1.5 to 2 times the recommended starting dose in patients who are CYP3A5 intermediate or extensive metabolizers, though total starting dose should not exceed 0.3 mg/kg/day. Therapeutic drug monitoring should also be used to guide dose adjustments. |
|  | **Recommendation:** Increase starting dose 1.5 to 2 times recommended starting dose. Total starting dose should not exceed 0.3mg/kg/day. Use therapeutic drug monitoring to guide dose adjustments |

### **tamoxifen**

**Gene**: CYP2D6    **Diplotype**: \*17/\*29    **Phenotype**: Intermediate Metabolizer

|  |  |
| --- | --- |
| **CPIC** | **Summary:** The CPIC Dosing Guideline for tamoxifen recommends the use of alternative hormonal therapy such as an aromatase inhibitor for postmenopausal women or aromatase inhibitor along with ovarian function suppression in premenopausal women for CYP2D6 poor metabolizer, if aromatase inhibitor use is not contraindicated. For CYP2D6 intermediate metabolizers and CYP2D6 allele combinations resulting in an activity score (AS) of 1 the recommendation is to consider the recommendations stated for the CYP2D6 poor metabolizer. If aromatase inhibitor use is contraindicated, consideration should be given to use a higher but FDA approved tamoxifen dose for CYP2D6 intermediate metabolizers and CYP2D6 allele combinations resulting in an AS of 1. For poor metabolizer, higher dose tamoxifen (40 mg/day) increases but does not normalize endoxifen concentrations and can be considered if there are contraindications to aromatase inhibitor therapy. |
|  | **Recommendation:** Consider hormonal therapy such as an aromatase inhibitor for postmenopausal women or aromatase inhibitor along with ovarian function suppression in premenopausal women, given that these approaches are superior to tamoxifen regardless of CYP2D6 genotype [Articles:26211827, 24881463]. If aromatase inhibitor use is contraindicated, consideration should be given to use a higher but FDA approved tamoxifen dose (40 mg/day) [Article:27226358]. Avoid CYP2D6 strong to weak inhibitors. |

|  |  |
| --- | --- |
| **CPNDS** | **Summary:** The Canadian Pharmacogenomics Network for Drug Safety (CPNDS) clinical recommendation group has published clinical practice guidelines for CYP2D6 as a treatment decision aid for ER-positive non-metastatic breast cancer patients. The guidelines recommend that alternatives to standard tamoxifen treatments may be considered in CYP2D6 poor or intermediate metabolizers with an emphasis on the use of comprehensive CYP2D6 genotyping panels in guiding treatment decisions. |
|  | **Recommendation:** aromatase inhibitor (AI) (with ovarian suppressor in premenopausal women) or Tamoxifen (40 mg/day) when AIs are contraindicated |

|  |  |
| --- | --- |
| **DPWG** | **Summary:** For CYP2D6 poor and intermediate metabolizers, consider an alternative medication or a dose increase. For intermediate metabolizers, avoid concomitant CYP2D6 inhibitor use. |
|  | **Recommendation:** 1. Select an alternative or measure the endoxifen concentration and increase the dose if necessary by a factor of 1.5-2. Aromatase inhibitors are a possible alternative for post-menopausal women. 2. If TAMOXIFEN is selected: avoid co-medication with CYP2D6 inhibitors such as paroxetine and fluoxetine |

### **tenoxicam**

**Gene**: CYP2C9    **Diplotype**: \*1/\*1    **Phenotype**: Normal Metabolizer

|  |  |
| --- | --- |
| **CPIC** | **Summary:** The CPIC Dosing Guideline for tenoxicam recommends that CYP2C9 poor metabolizers and intermediate metabolizers with activity score of 1 should choose an alternative therapy not metabolized by CYP2C9 or not significantly impacted by CYP2C9 genetic variants in vivo or choose an NSAID metabolized by CYP2C9 but with a shorter half-life. See full guideline for further details and supporting evidence. |
|  | **Recommendation:** Initiate therapy with recommended starting dose. In accordance with the prescribing information, use the lowest effective dosage for shortest duration consistent with individual patient treatment goals. |

### **thioguanine**

**Gene**: TPMT    **Diplotype**: \*1/\*1    **Phenotype**: Normal Metabolizer

|  |  |
| --- | --- |
| **CPIC** | **Summary:** Consider an alternate agent or extreme dose reduction of thioguanine for patients who are TPMT or NUDT15 poor metabolizers. Start at 50-80% of target dose for patients who are TPMT or NUDT15 intermediate metabolizers. |
|  | **Recommendation:** Start with normal starting dose (e.g. 40-60 mg/m2/day) and adjust doses of thioguanine and of other myelosuppressive therapy without any special emphasis on thioguanine. Allow 2 weeks to reach steady-state after each dose adjustment. |

**Gene**: NUDT15    **Diplotype**: \*1/\*1    **Phenotype**: Normal Metabolizer

|  |  |
| --- | --- |
| **CPIC** | **Summary:** Consider an alternate agent or extreme dose reduction of thioguanine for patients who are TPMT or NUDT15 poor metabolizers. Start at 50-80% of target dose for patients who are TPMT or NUDT15 intermediate metabolizers. |
|  | **Recommendation:** Start with normal starting dose (40-60 mg/day). Adjust doses of thioguanine and of other myelosuppressive therapy without any special emphasis on thioguanine. Allow 2 weeks to reach steady-state after each dose adjustment. |

### **tramadol**

**Gene**: CYP2D6    **Diplotype**: \*17/\*29    **Phenotype**: Intermediate Metabolizer

|  |  |
| --- | --- |
| **CPIC** | **Summary:** Alternate non-codeine analgesics are recommended for CYP2D6 ultrarapid and poor metabolizers. A label recommended age- or weight-specific dose of tramadol is warranted for CYP2D6 normal and intermediate metabolizers. |
|  | **Recommendation:** Use tramadol label recommended age- or weight-specific dosing. If no response and opioid use is warranted, consider a non-codeine opioid |

|  |  |
| --- | --- |
| **DPWG** | **Summary:** Be alert to a reduced efficacy of tramadol in CYP2D6 intermediate or poor metabolizers. If tramadol is not effective in these patients, try a dose increase or select an alternative to tramadol (not codeine) and be alert for symptoms of insufficient pain relief. For CYP2D6 ultrarapid metabolizers, use an alternative to tramadol (not codeine) or use 40% of the standard dose and be alert to side effects. |
|  | **Recommendation:** It is not possible to provide a recommendation for dose adjustment, because the total analgesic effect changes when the ratio between the mother compound and the active metabolite changes. 1. be alert to a reduced effectiveness 2. in the case of inadequate effectiveness: a. try a dose increase b. if this does not work: choose an alternative. Do not select codeine, as this is also metabolised by CYP2D6. Morphine is not metabolised by CYP2D6. Oxycodone is metabolised by CYP2D6 to a limited extent, but this does not result in differences in analgesia in patients. 3. if no alternative is selected: advise the patient to report inadequate analgesia |

### **trimipramine**

**Gene**: CYP2C19    **Diplotype**: \*1/\*17    **Phenotype**: Rapid Metabolizer

|  |  |
| --- | --- |
| **CPIC** | **Summary:** Tricyclic antidepressants have comparable pharmacokinetic properties, it may be reasonable to apply the CPIC Dosing Guideline for amitriptyline and CYP2C19, CYP2D6 to other tricyclics including trimipramine. The CPIC Dosing Guideline update for amitriptyline recommends an alternative drug for CYP2D6 ultrarapid or poor metabolizers and CYP2C19 ultrarapid, rapid or poor metabolizers. If amitriptyline is warranted, consider a 50% dose reduction in CYP2D6 or CYP2C19 poor metabolizers. For CYP2D6 intermediate metabolizers, a 25% dose reduction should be considered. |
|  | **Recommendation:** Avoid tertiary amine use due to potential for sub-optimal response. Consider alternative drug not metabolized by CYP2C19. TCAs without major CYP2C19 metabolism include the secondary amines nortriptyline and desipramine. If a tertiary amine is warranted, utilize therapeutic drug monitoring to guide dose adjustments. |

**Gene**: CYP2D6    **Diplotype**: \*17/\*29    **Phenotype**: Intermediate Metabolizer

|  |  |
| --- | --- |
| **CPIC** | **Summary:** Tricyclic antidepressants have comparable pharmacokinetic properties, it may be reasonable to apply the CPIC Dosing Guideline for amitriptyline and CYP2C19, CYP2D6 to other tricyclics including trimipramine. The CPIC Dosing Guideline update for amitriptyline recommends an alternative drug for CYP2D6 ultrarapid or poor metabolizers and CYP2C19 ultrarapid, rapid or poor metabolizers. If amitriptyline is warranted, consider a 50% dose reduction in CYP2D6 or CYP2C19 poor metabolizers. For CYP2D6 intermediate metabolizers, a 25% dose reduction should be considered. |
|  | **Recommendation:** Consider 25% reduction of recommended starting dose. Utilize therapeutic drug monitoring to guide dose adjustments. |

### **tropisetron**

**Gene**: CYP2D6    **Diplotype**: \*17/\*29    **Phenotype**: Intermediate Metabolizer

|  |  |
| --- | --- |
| **CPIC** | **Summary:** The CPIC dosing guideline for tropisetron recommends selecting an alternate drug for CYP2D6 ultrarapid metabolizers. It is recommended that the alternate drug not be predominantly metabolized by CYP2D6 (eg. granisetron). |
|  | **Recommendation:** Insufficient evidence demonstrating clinical impact based on CYP2D6 genotype. Initiate therapy with recommended starting dose. |

### **venlafaxine**

**Gene**: CYP2D6    **Diplotype**: \*17/\*29    **Phenotype**: Intermediate Metabolizer

|  |  |
| --- | --- |
| **DPWG** | **Summary:** For CYP2D6 poor (PM) and intermediate metabolizers (IM), select an alternative to venlafaxine or reduce the dose and monitor patient's plasma metabolite level. For CYP2D6 ultrarapid metabolizers (UM), increase dose to 150% of the normal dose or select an alternative to venlafaxine. |
|  | **Recommendation:** It is not possible to offer adequately substantiated advice for dose reduction based on the literature. - avoid venlafaxine. Antidepressants that are not metabolised by CYP2D6 - or to a lesser extent - include, for example, duloxetine, mirtazapine, citalopram and sertraline. - if it is not possible to avoid venlafaxine and side effects occur: 1. reduce the dose 2. monitor the effect and side effects or check the plasma concentrations of venlafaxine and O-desmethylvenlafaxine. It is not known whether it is possible to reduce the dose to such an extent that the side effects disappear, while the effectiveness is maintained. In general, it is assumed that the effectiveness is determined by the sum of the plasma concentrations of venlafaxine and O-desmethylvenlafaxine. However, the side effects do not appear to be related to this sum. |

### **voriconazole**

**Gene**: CYP2C19    **Diplotype**: \*1/\*17    **Phenotype**: Rapid Metabolizer

|  |  |
| --- | --- |
| **CPIC** | **Summary:** The CPIC dosing guideline for voriconazole recommends selecting an alternative agent that is not dependent on CYP2C19 metabolism in adults who are CYP2C19 ultrarapid metabolizers, rapid metabolizers or poor metabolizers. In pediatric patients, an alternative agent should be used in patients who are ultrarapid metabolizers or poor metabolizers. In pediatric rapid metabolizers, therapy should be initiated at recommended standard case dosing, then therapeutic dosing monitoring should be used to titrate dose to therapeutic trough concentrations. |
|  | **Recommendation:** Choose an alternative agent that is not dependent on CYP2C19 metabolism as primary therapy in lieu of voriconazole. Such agents include isavuconazole, liposomal amphotericin B, and posaconazole. |

### **warfarin**

**Gene**: VKORC1    **Diplotype**: C/T    **Phenotype**: -

|  |  |
| --- | --- |
| **CPIC** | **Summary:** The updated guideline for pharmacogenetics-guided warfarin dosing is published by the Clinical Pharmacogenetics Implementation Consortium. The recommendations for dosing are for adult and pediatric patients that are specific to continental ancestry, and are based on genotypes from CYP2C9, VKORC1, CYP4F2, and rs12777823. |
|  | **Recommendation:** A decreased dose of warfarin. Calculate warfarin dose using a validated pharmacogenetic algorithm [Articles:18305455, 19228618]. |

### **zuclopenthixol**

**Gene**: CYP2D6    **Diplotype**: \*17/\*29    **Phenotype**: Intermediate Metabolizer

|  |  |
| --- | --- |
| **DPWG** | **Summary:** For CYP2D6 poor and intermediate metabolizers, reduce zuclopenthixol dose or select an alternative drug that is not metabolized by CYP2D6. For ultrarapid metabolizers, be alert to low zuclopenthixol plasma concentrations and, if necessary, increase the dose or select an alternative drug. |
|  | **Recommendation:** Use 75% of the standard dose. |

## **Diplotype Detail**

### **Multi-variant allele**

PAnno ranking model is applied to predict diplotypes consisting of multiple variants. The diplotypes are inferred by integrating allele definition consistency as well as the population allele frequency. PGx genes include CYP2B6, CYP2C19, CYP2C8, CYP2C9, CYP2D6, CYP3A4, CYP3A5, CYP4F2, DPYD, NUDT15, SLCO1B1, TPMT, and UGT1A1. Note that PAnno assumes that no variation occurs for the missing positions in the submitted VCF file.

### **CYP2B6: \*1/\*18**

Please notice that CYP2B6\*29, CYP2B6\*30 are not considered in the current version, which could potentially have an impact on the results.

| Position | Variant | Effect on Protein | Definition of \*1 | Definition of \*18 | Variant Call |
| --- | --- | --- | --- | --- | --- |
| chr19:40991224 | rs34223104 |  | T | T | T/T |
| chr19:40991367 | rs34883432 | p.Q21L | A | A | Missing |
| chr19:40991369 | rs8192709 | p.R22C | C | C | Missing |
| chr19:40991381 | rs33973337 | p.T26S | A | A | A/A |
| chr19:40991388 | rs33980385 | p.D28G | A | A | Missing |
| chr19:40991390 | rs33926104 | p.R29S | C | C | Missing |
| chr19:40991391 | rs34284776 | p.R29P | G | G | Missing |
| chr19:40991441 | rs35303484 | p.M46V | A | A | Missing |
| chr19:41004015 | rs281864907 | p.Y62X | T | T | Missing |
| chr19:41004125 | rs36060847 | p.G99E | G | G | G/G |
| chr19:41004158 | rs186335453 | p.G110V | G | G | Missing |
| chr19:41004303 | rs139801276 | p.I114T | T | T | Missing |
| chr19:41004377 | rs12721655 | p.K139E | A | A | Missing |
| chr19:41004381 | rs35773040 | p.R140Q | G | G | Missing |
| chr19:41004406 | rs145884402 | p.E148D | G | G | Missing |
| chr19:41006919 | rs3826711 | p.P167A | C | C | Missing |
| chr19:41006923 | rs36056539 | p.T168I | C | C | Missing |
| chr19:41006936 | rs3745274 | p.Q172H | G | G | Missing |
| chr19:41006968 | rs373489637 | p.V183G | T | T | Missing |
| chr19:41007013 | rs36079186 | p.M198T | T | T | Missing |
| chr19:41009350 | rs45482602 | p.S259R | C | C | C/C |
| chr19:41009358 | rs2279343 | p.K262R | A | R | A/A |
| chr19:41010006 | rs139029625 | p.A279P | G | G | Missing |
| chr19:41010088 | rs34698757 | p.T306S | C | C | Missing |
| chr19:41010108 | rs193922917 | p.L313I | C | C | Missing |
| chr19:41012316 | rs28399499 | p.I328T | T | C | T/C |
| chr19:41012339 | rs34826503 | p.R336C | C | C | Missing |
| chr19:41012465 | rs34097093 | p.R378X | C | C | Missing |
| chr19:41012693 | rs35979566 | p.I391N | T | T | T/T |
| chr19:41012740 | rs193922918 | p.A407T | G | G | Missing |
| chr19:41012803 | rs35010098 | p.P428T | C | C | Missing |
| chr19:41016726 | rs3211369 | p.M459V | A | A | Missing |
| chr19:41016778 | rs564083989 | p.G476D | G | G | Missing |
| chr19:41016805 |  | p.Q485L | A | A | Missing |
| chr19:41016810 | rs3211371 | p.R487S; p.R487C | C | C | Missing |

### **CYP2C8: \*1/\*1**

| Position | Variant | Effect on Protein | Definition of \*1 | Variant Call |
| --- | --- | --- | --- | --- |
| chr10:95067273 | rs11572080 | p.R139K | C | C/C |
| chr10:95067218 | rs72558196 | p.T159fs | T | Missing |
| chr10:95064931 | rs142886225 | p.G171S | C | Missing |
| chr10:95064901 | rs41286886 | p.V181I | C | Missing |
| chr10:95064886 | rs72558195 | p.R186G; p.R186X | G | Missing |
| chr10:95058485 |  | p.I223M | A | Missing |
| chr10:95058442 | rs188934928 | p.A238P | C | C/C |
| chr10:95058424 | rs11572102 | p.I244V | T | Missing |
| chr10:95058414 | rs769460274 | p.K247R | T | Missing |
| chr10:95058362 | rs1058930 | p.I264M | G | Missing |
| chr10:95058349 | rs11572103 | p.I269F | T | Missing |
| chr10:95045951 | rs78637571 | p.E274X | C | Missing |
| chr10:95043047 | rs146806199 | p.I331T | A | Missing |
| chr10:95042958 | rs45438799 | p.L361F | G | Missing |
| chr10:95042890 |  | p.K383N | C | Missing |
| chr10:95038992 | rs10509681 | p.K399R | T | Missing |
| chr10:95037219 | rs3832694 | p.461delV | ACA | ACA/ACA |

### **CYP2C9: \*1/\*1**

| Position | Variant | Effect on Protein | Definition of \*1 | Variant Call |
| --- | --- | --- | --- | --- |
| chr10:94942306 | rs1289704600 | p.A149V | C | Missing |
| chr10:94942308 | rs17847037 | p.R150C | C | C/C |
| chr10:94947439 |  | p.Q214H | G | Missing |
| chr10:94986136 | rs1254213342 | p.N418T | A | Missing |
| chr10:94942213 | rs1304490498 | p.K118fs | AGAAATGGAA | AGAAATGGAA/AGAAATGGAA |
| chr10:94949283 | rs9332131 | p.K273fs | A | Missing |
| chr10:94986042 | rs764211126 | p.I387V | A | Missing |
| chr10:94986073 | rs72558193 | p.D397A | A | Missing |
| chr10:94988852 | rs776908257 | p.R433W | C | Missing |
| chr10:94941976 |  | p.G96A | G | Missing |
| chr10:94942309 | rs7900194 | p.R150H; p.R150L | G | Missing |
| chr10:94947869 |  | p.D191G | A | Missing |
| chr10:94981302 | rs1250577724 | p.L361I | C | Missing |
| chr10:94938683 | rs114071557 | p.M1V | A | Missing |
| chr10:94938737 | rs67807361 | p.L19I | C | Missing |
| chr10:94938771 | rs142240658 | p.P30L | C | Missing |
| chr10:94938803 | rs2031308986 | p.N41D | A | A/A |
| chr10:94938828 | rs564813580 | p.D49G | A | A/A |
| chr10:94941897 | rs371055887 | p.G70R | G | Missing |
| chr10:94941915 |  | p.V76M | G | Missing |
| chr10:94941958 | rs72558187 | p.L90P | T | Missing |
| chr10:94941982 | rs762239445 | p.G98V | G | G/G |
| chr10:94942018 |  | p.F110S | T | Missing |
| chr10:94942216 | rs774607211 | p.K119R | A | Missing |
| chr10:94942230 | rs767576260 | p.R124W | C | Missing |
| chr10:94942231 | rs12414460 | p.R124Q | G | Missing |
| chr10:94942233 | rs375805362 | p.R125C | C | Missing |
| chr10:94942234 | rs72558189 | p.R125H; p.R125L | G | G/G |
| chr10:94942249 | rs200965026 | p.T130R; p.T130M | C | Missing |
| chr10:94942254 | rs199523631 | p.R132W | C | Missing |
| chr10:94942255 | rs200183364 | p.R132Q | G | G/G |
| chr10:94942290 | rs1799853 | p.R144C | C | C/C |
| chr10:94942291 | rs141489852 | p.R144H | G | G/G |
| chr10:94942305 | rs754487195 | p.A149T | G | Missing |
| chr10:94947782 | rs72558190 | p.S162X | C | Missing |
| chr10:94947785 | rs774550549 | p.P163L | C | Missing |
| chr10:94988855 |  | p.I434F | A | Missing |
| chr10:94981296 | rs1057910 | p.I359L | A | Missing |
| chr10:94988917 | rs769942899 | p.Q454H | G | Missing |
| chr10:94981225 | rs367826293 | p.R335Q | G | Missing |
| chr10:94981250 | rs750820937 | p.S343R | C | Missing |
| chr10:94988955 | rs767284820 | p.L467P | T | Missing |
| chr10:94981301 | rs28371686 | p.D360E | C | Missing |
| chr10:94989020 | rs9332239 | p.P489S | C | Missing |
| chr10:94981305 | rs578144976 | p.L362V | C | Missing |
| chr10:94988925 | rs202201137 | p.N457S | A | A/A |
| chr10:94988984 | rs781583846 | p.A477T | G | Missing |
| chr10:94989023 | rs868182778 | p.V490F | G | Missing |
| chr10:94981365 |  | p.P382S | C | Missing |
| chr10:94988880 |  | p.G442V | G | G/G |
| chr10:94947907 |  | p.N204H | A | Missing |
| chr10:94947917 | rs1326630788 | p.I207T | T | T/T |
| chr10:94947938 | rs2031531005 | p.Q214L | A | Missing |
| chr10:94949129 |  | p.I222V | A | Missing |
| chr10:94949144 |  | p.P227S | C | Missing |
| chr10:94949217 | rs2256871 | p.H251R | A | Missing |
| chr10:94949280 | rs9332130 | p.E272G | A | Missing |
| chr10:94972119 | rs182132442 | p.P279T | C | Missing |
| chr10:94972123 |  | p.S280C | C | Missing |
| chr10:94972134 |  | p.I284V | A | Missing |
| chr10:94972179 | rs72558192 | p.T299A | A | Missing |
| chr10:94972180 | rs988617574 | p.T299R | C | Missing |
| chr10:94972233 | rs1237225311 | p.P317S | C | Missing |
| chr10:94981199 |  | p.E326D | G | G/G |
| chr10:94981201 | rs57505750 | p.I327T | T | Missing |
| chr10:94981224 | rs28371685 | p.R335W | C | Missing |
| chr10:94981230 | rs1274535931 | p.P337T | C | Missing |
| chr10:94981281 | rs749060448 | p.E354K | G | G/G |
| chr10:94981297 | rs56165452 | p.I359T | T | Missing |
| chr10:94981371 | rs542577750 | splicing defect | G | Missing |

### **CYP2C19: \*1/\*17**

Please notice that CYP2C19\*36, CYP2C19\*37 are not considered in the current version, which could potentially have an impact on the results.

| Position | Variant | Effect on Protein | Definition of \*1 | Definition of \*17 | Variant Call |
| --- | --- | --- | --- | --- | --- |
| chr10:94761900 | rs12248560 | p.expression | C | T | C/T |
| chr10:94762706 | rs28399504 | p.M1V | A | A | Missing |
| chr10:94762712 | rs367543002 | p.P3S | C | C | Missing |
| chr10:94762715 | rs367543003 | p.F4L | T | T | Missing |
| chr10:94762755 | rs55752064 | p.L17P | T | T | Missing |
| chr10:94762760 | rs17882687 | p.I19L | A | A | Missing |
| chr10:94762788 | rs1564656981 | p.K28I | A | A | A/A |
| chr10:94762856 | rs1564657013 | p.S51G | A | A | Missing |
| chr10:94775106 | rs145328984 | p.R73C | C | C | Missing |
| chr10:94775121 | rs1564660997 | p.H78Y | C | C | Missing |
| chr10:94775160 | rs118203756 | p.G91R | G | G | Missing |
| chr10:94775185 | rs1288601658 | p.H99R | A | A | Missing |
| chr10:94775367 | rs12769205 | splicing defect | A | A | Missing |
| chr10:94775416 | rs41291556 | p.W120R | T | T | Missing |
| chr10:94775423 | rs17885179 | p.E122A | A | A | Missing |
| chr10:94775453 | rs72552267 | p.R132Q | G | G | G/G |
| chr10:94775489 | rs17884712 | p.R144H | G | G | Missing |
| chr10:94775507 | rs58973490 | p.R150H | G | G | G/G |
| chr10:94780574 | rs140278421 | p.R186P | G | G | Missing |
| chr10:94780579 | rs370803989 | p.D188N | G | G | Missing |
| chr10:94780653 | rs4986893 | p.W212X | G | G | Missing |
| chr10:94781858 | rs6413438 | p.P227L | C | C | C/C |
| chr10:94781859 | rs4244285 | splicing defect | G | G | Missing |
| chr10:94781944 | rs375781227 | p.D256N | G | G | Missing |
| chr10:94781999 | rs72558186 | splicing defect | T | T | Missing |
| chr10:94842861 | rs138142612 | p.R329H | G | G | Missing |
| chr10:94842866 | rs3758581 | p.I331V | G | G | G/G |
| chr10:94842879 | rs118203757 | p.R335Q | G | G | Missing |
| chr10:94842995 | rs113934938 | p.V374I | G | G | Missing |
| chr10:94849995 | rs17879685 | p.R410C | C | C | Missing |
| chr10:94852738 | rs56337013 | p.R433W | C | C | Missing |
| chr10:94852765 | rs192154563 | p.R442C | C | C | Missing |
| chr10:94852785 | rs118203759 | p.F448L | C | C | Missing |
| chr10:94852914 | rs55640102 | p.X491C | A | A | Missing |

### **CYP2D6: \*17/\*29**

Please notice that CYP2D6\*5, CYP2D6\*13, CYP2D6\*61, CYP2D6\*63, CYP2D6\*68 and CYP2D6 CNVs are not considered in the current version, which could potentially have an impact on the results.

| Position | Variant | Effect on Protein | Definition of \*17 | Definition of \*29 | Variant Call |
| --- | --- | --- | --- | --- | --- |
| chr22:42129042 | rs1135824 | p.N166D | T | T | Missing |
| chr22:42129033 | rs5030865 | p.G169R; p.G169X | C | C | Missing |
| chr22:42128945 | rs3892097 | splicing defect | C | C | Missing |
| chr22:42128934 | rs72549356 | p.174\_175insFRPx2; p.174\_175insFRP | AAAGGGGCG | AAAGGGGCG | Missing |
| chr22:42128878 |  | p.C191F | C | C | Missing |
| chr22:42128813 | rs150163869 |  | R | R | Missing |
| chr22:42128351 | rs377725912 | splicing defect | C | C | C/C |
| chr22:42130729 |  | p.L22X | G | G | Missing |
| chr22:42130655 | rs774671100 | p.L47fs | A | A | Missing |
| chr22:42129799 | rs76802407 | p.D97E | G | G | Missing |
| chr22:42129084 | rs5030655 | p.W152fs | A | A | Missing |
| chr22:42128817 | rs72549354 | p.L213fs | C | C | Missing |
| chr22:42128796 |  | p.L220fs | G | G | Missing |
| chr22:42128251 | rs72549353 | p.T256fs | TTAG | TTAG | Missing |
| chr22:42128242 | rs35742686 | p.R259fs | T | T | Missing |
| chr22:42128218 | rs72549352 | p.R269fs | G | G | G/G |
| chr22:42128199 | rs72549351 | p.T272fs | TCAG(2) | TCAG(2) | TCAG(2)/TCAG(2) |
| chr22:42128176 | rs5030656 | p.K281del | TCT | TCT | TCT/TCT |
| chr22:42127963 | rs267608279 | p.S288fs | G | G | Missing |
| chr22:42127846 | rs730882170 | p.M321fs | CACATCCGGATGTAGGATC | CACATCCGGATGTAGGATC | CACATCCGGATGTAGGATC/CACATCCGGATGTAGGATC |
| chr22:42126982 | rs757396767 | p.L395fs | AG | AG | Missing |
| chr22:42126658 | rs765776661 | p.468\_470dupVPT | AGTGGGCAC | AGTGGGCAC | AGTGGGCAC/AGTGGGCAC |
| chr22:42127973 | rs1135829 | p.N285S | T | T | T/T |
| chr22:42129809 | rs28371704 | p.H94R | T | T | Missing |
| chr22:42129770 | rs28371706 | p.Y107I; p.T107N | A | G | G/A |
| chr22:42129071 | rs267608302 | p.E156A; p.E156V | T | T | Missing |
| chr22:42128903 |  | p.S183X | del | del | Missing |
| chr22:42127922 | rs1406719554 | p.L302P | A | A | Missing |
| chr22:42129183 | rs374616348 | p.V119M | C | C | Missing |
| chr22:42130778 | rs773790593 | p.A5V | G | G | Missing |
| chr22:42130773 | rs72549358 | p.V7M | C | C | Missing |
| chr22:42130719 | rs267608313 | p.R25W | G | G | Missing |
| chr22:42130715 | rs28371696 | p.R26H | C | C | Missing |
| chr22:42130710 | rs138100349 | p.R28C | G | G | Missing |
| chr22:42130692 | rs1065852 | p.P34S | G | G | Missing |
| chr22:42130668 | rs5030862 | p.G42R | C | C | Missing |
| chr22:42130667 | rs118203758 | p.G42E | C | C | C/C |
| chr22:42129910 | rs201377835 | splicing defect | C | C | Missing |
| chr22:42129906 | rs267608311 | p.R62W | G | G | Missing |
| chr22:42129887 | rs1456026511 | p.V68G | A | A | Missing |
| chr22:42129836 | rs267608310 | p.A85V | G | G | Missing |
| chr22:42129827 | rs267608276 | p.R88P | C | C | Missing |
| chr22:42129821 | rs267608309 | p.A90V | G | G | Missing |
| chr22:42129819 | rs28371703 | p.L91M | G | G | Missing |
| chr22:42129780 | rs267608308 | p.V104M | C | C | Missing |
| chr22:42129779 | rs76187628 | p.V104A | A | A | Missing |
| chr22:42129771 | rs74802369 | p.T107S | T | T | T/T |
| chr22:42129765 | rs78459009 | p.I109V | T | T | Missing |
| chr22:42129759 | rs535642512 | p.G111S | C | C | Missing |
| chr22:42129180 | rs1135822 | p.F120I | A | A | Missing |
| chr22:42129174 | rs1135823 | p.A122S | C | C | Missing |
| chr22:42129166 | rs766391487 | p.Y124X | A | A | Missing |
| chr22:42129155 | rs1180015037 | p.W128X | C | C | Missing |
| chr22:42129134 | rs781457579 | p.S135F | G | G | Missing |
| chr22:42129132 | rs61736512 | p.V136M | C | T | C/T |
| chr22:42129130 | rs1058164 |  | G | G | G/G |
| chr22:42129113 | rs375135093 | p.L142S | A | A | Missing |
| chr22:42129098 | rs569229126 | p.K147R | T | T | Missing |
| chr22:42129087 | rs78482768 | p.Q151E | G | G | Missing |
| chr22:42129075 | rs28371710 | p.E155K | C | C | Missing |
| chr22:42129056 |  | p.C161S | C | C | Missing |
| chr22:42129037 | rs1135825 | p.H167Q | G | G | Missing |
| chr22:42129036 | rs1135826 | p.S168A | A | A | Missing |
| chr22:42126914 | rs28371733 | p.E418K | C | C | Missing |
| chr22:42127457 | rs77312092 | p.R388H | C | C | Missing |
| chr22:42126956 | rs1931013246 | p.K404Q | T | T | Missing |
| chr22:42127523 | rs1555888910 | p.F366S | A | A | Missing |
| chr22:42126896 | rs763964554 | p.Q424X | G | G | Missing |
| chr22:42127514 |  | p.I369T | A | A | Missing |
| chr22:42126877 | rs3021084 | p.P430L | G | G | Missing |
| chr22:42126752 | rs569439709 | p.G439D | C | C | C/C |
| chr22:42126749 | rs267608319 | p.R440H | C | C | Missing |
| chr22:42126747 | rs730882251 | p.R441C | G | G | G/G |
| chr22:42126746 | rs532668079 | p.R441H | C | C | C/C |
| chr22:42127512 | rs61745683 | p.V370I | C | C | C/T |
| chr22:42127473 | rs75386357 | p.E383K | C | C | Missing |
| chr22:42126735 | rs751092905 | p.G445R | C | C | Missing |
| chr22:42126719 | rs369177208 | p.R450H | C | C | Missing |
| chr22:42126697 |  | p.F457L | G | G | Missing |
| chr22:42126681 |  | p.H463D | G | G | Missing |
| chr22:42126663 | rs1135833 | p.P469A | G | G | G/G |
| chr22:42126660 | rs1135835 | p.T470A | T | T | Missing |
| chr22:42126647 | rs141756339 | p.R474Q | C | C | Missing |
| chr22:42126636 | rs28371735 | p.H478Y | G | G | Missing |
| chr22:42126635 | rs766507177 | p.H478P | T | T | T/T |
| chr22:42126634 |  | p.H478Q | A | A | A/A |
| chr22:42126633 |  | p.G479R | C | C | Missing |
| chr22:42126627 |  | p.F481V | A | A | Missing |
| chr22:42126624 | rs74478221 | p.A482T | C | C | Missing |
| chr22:42126623 | rs75467367 | p.A482G | G | G | Missing |
| chr22:42126611 | rs1135840 | p.S486T | G | G | G/G |
| chr22:42126605 | rs568495591 | p.S488F | G | G | Missing |
| chr22:42126578 | rs1440526469 | p.R497H | C | C | Missing |
| chr22:42128879 |  | p.C191R | A | A | Missing |
| chr22:42128848 | rs745365204 | p.R201H | C | C | Missing |
| chr22:42128812 | rs199535154 | p.L213P | A | A | Missing |
| chr22:42128329 | rs373813287 | p.L230F | G | G | Missing |
| chr22:42128325 | rs17002853 | p.L231P | A | A | Missing |
| chr22:42128308 | rs28371717 | p.A237S | C | C | Missing |
| chr22:42128272 |  | p.T249P | T | T | Missing |
| chr22:42128235 | rs267608297 | p.T261I | G | G | Missing |
| chr22:42128217 | rs148769737 | p.P267H | G | G | Missing |
| chr22:42128212 | rs367543000 | p.R269X | G | G | G/G |
| chr22:42128185 | rs77913725 | p.E278K | C | C | Missing |
| chr22:42128181 | rs1135828 | p.M279K | A | A | Missing |
| chr22:42127941 | rs16947 | p.R296C | A | A | A/A |
| chr22:42127938 | rs949717872 | p.I297L | T | T | Missing |
| chr22:42127899 |  | p.T310A | T | T | Missing |
| chr22:42127856 | rs5030867 | p.H324P | T | T | T/T |
| chr22:42127852 | rs79292917 | splicing defect | C | C | Missing |
| chr22:42127841 | rs72549349 | splicing defect | C | C | Missing |
| chr22:42127803 | rs28371725 | splicing defect | C | C | Missing |
| chr22:42127631 | rs141009491 | p.R330P | C | C | Missing |
| chr22:42127619 | rs72549348 | p.E334A | T | T | Missing |
| chr22:42127611 | rs78209835 | p.D337N | C | C | Missing |
| chr22:42127610 | rs748712690 | p.D337G | T | T | Missing |
| chr22:42127608 | rs59421388 | p.V338M | C | T | C/T |
| chr22:42127602 |  | p.G340R | C | C | Missing |
| chr22:42127593 | rs267608295 | p.R343G | G | G | Missing |
| chr22:42127590 | rs72549347 | p.R344X | G | G | Missing |
| chr22:42127589 | rs76088846 | p.R344Q | C | C | Missing |
| chr22:42127565 | rs61736517 | p.H352R | T | T | Missing |
| chr22:42127556 | rs202102799 | p.Y355C | T | T | Missing |
| chr22:42127530 | rs72549346 | p.Q364fs | del | del | Missing |
| chr22:42127526 | rs1058172 | p.R365H | C | C | Missing |
| chr22:42126938 | rs769157652 | p.E410K | C | C | C/C |
| chr22:42126926 | rs747089665 | p.R414C | G | G | Missing |
| chr22:42130761 | rs769258 | p.V11M | C | C | Missing |

### **CYP3A4: \*1/\*36**

| Position | Variant | Effect on Protein | Definition of \*1 | Definition of \*36 | Variant Call |
| --- | --- | --- | --- | --- | --- |
| chr7:99784075 | rs188389063 | p.L3V | G | G | Missing |
| chr7:99784038 | rs12721634 | p.L15P | A | A | Missing |
| chr7:99784018 | rs570051168 | p.L22V | G | G | Missing |
| chr7:99778079 | rs56324128 | p.G56D | C | C | C/C |
| chr7:99770217 | rs1449865051 | p.F113I | A | A | Missing |
| chr7:99770202 | rs55951658 | p.I118V | T | T | Missing |
| chr7:99770166 | rs778013004 | p.R130X | G | G | Missing |
| chr7:99770165 | rs72552799 | p.R130Q | C | C | Missing |
| chr7:99769805 | rs57409622 | p.R162W | G | G | Missing |
| chr7:99769804 | rs4986907 | p.R162Q | C | C | Missing |
| chr7:99769781 | rs72552798 | p.V170I | C | C | Missing |
| chr7:99769769 | rs4986908 | p.D174H | C | C | Missing |
| chr7:99768693 | rs35599367 | splicing defect | G | G | Missing |
| chr7:99768470 | rs12721627 | p.T185S | G | G | Missing |
| chr7:99768458 | rs4987161 | p.F189S | A | A | Missing |
| chr7:99768424 | rs113667357 | p.Q200H | T | T | T/T |
| chr7:99768371 | rs55901263 | p.P218R | G | G | Missing |
| chr7:99768360 | rs55785340 | p.S222P | A | A | Missing |
| chr7:99766440 | rs138105638 | p.R268X | G | G | Missing |
| chr7:99766412 | rs4646438 | p.D277fs | T | T | Missing |
| chr7:99764003 | rs28371759 | p.L293P | A | A | Missing |
| chr7:99763925 | rs201821708 | p.Y319C | T | T | Missing |
| chr7:99763909 | rs1303250043 | p.H324Q | G | G | Missing |
| chr7:99763877 | rs368296206 | p.I335T | A | A | Missing |
| chr7:99763843 | rs2242480 | p.expression | C | T | C/T |
| chr7:99762206 | rs67784355 | p.T363M | G | G | Missing |
| chr7:99762186 | rs756833413 | p.A370S | C | C | Missing |
| chr7:99762177 | rs12721629 | p.L373F | G | G | Missing |
| chr7:99762047 | rs4986909 | p.P416L | G | G | Missing |
| chr7:99760956 | rs774109750 | p.I427V | T | T | Missing |
| chr7:99760901 | rs4986910 | p.M445T | A | A | Missing |
| chr7:99760836 | rs4986913 | p.P467S | G | G | Missing |
| chr7:99758188 | rs67666821 | p.P488fs | T | T | Missing |

### **CYP3A5: \*1/\*3**

| Position | Variant | Effect on Protein | Definition of \*1 | Definition of \*3 | Variant Call |
| --- | --- | --- | --- | --- | --- |
| chr7:99652771 | rs41303343 | p.T346fs | A | A | Missing |
| chr7:99676198 | rs55817950 | p.R28C | G | G | Missing |
| chr7:99665212 | rs10264272 | splicing defect | C | C | Missing |
| chr7:99672916 | rs776746 | splicing defect | T | C | T/C |
| chr7:99660516 | rs28383479 | p.A337T | C | C | Missing |

### **CYP4F2: \*1/\*1**

| Position | Variant | Effect on Protein | Definition of \*1 | Variant Call |
| --- | --- | --- | --- | --- |
| chr19:15897578 | rs3093105 | p.W12G | A | Missing |
| chr19:15879621 | rs2108622 | p.V433M | C | Missing |

### **DPYD: c.85T>C (\*9A)/c.1349C>T**

| Position | Variant | Effect on Protein | Definition of c.85T>C (\*9A) | Definition of c.1349C>T | Variant Call |
| --- | --- | --- | --- | --- | --- |
| chr1:97740411 | rs72549309 |  | ATGA(2) | ATGA(2) | ATGA(2)/ATGA(2) |
| chr1:97450067 | rs72549303 | p.P633Qfs | G | G | Missing |
| chr1:97699399 | rs72549307 | p.Y211C | T | T | Missing |
| chr1:97691776 | rs1801266 | p.R235W | G | G | G/G |
| chr1:97679170 | rs45589337 | p.K259E | T | T | Missing |
| chr1:97595149 | rs146356975 | p.K290E | T | T | Missing |
| chr1:97595088 | rs150437414 | p.L310S | A | A | Missing |
| chr1:97595083 | rs145112791 | p.L312F | G | G | Missing |
| chr1:97593379 | rs201018345 | p.A323T | C | C | Missing |
| chr1:97593343 | rs72549306 | p.V335L | C | C | Missing |
| chr1:97573839 | rs200064537 | p.N420K | A | A | Missing |
| chr1:97373629 | rs138545885 | p.A664S | C | C | Missing |
| chr1:97593289 | rs143154602 | p.R353C | G | G | Missing |
| chr1:97593238 | rs72549305 | p.I370V | T | T | Missing |
| chr1:97699533 | rs139834141 | p.M166I | C | C | Missing |
| chr1:97699506 | rs6670886 | p.S175S | C | C | Missing |
| chr1:97579893 | rs75017182 |  | G | G | Missing |
| chr1:97699474 | rs115232898 | p.Y186C | T | T | T/T |
| chr1:97573943 | rs78060119 | p.E386X | C | C | Missing |
| chr1:97573918 | rs143815742 | p.R394L | C | C | C/C |
| chr1:97573881 | rs61622928 | p.M406I | C | C | Missing |
| chr1:97573863 | rs56038477 |  | C | C | Missing |
| chr1:97883329 | rs1801265 | p.C29R | G | A | A/G |
| chr1:97573821 | rs764666241 | p.M426I | C | C | Missing |
| chr1:97573805 | rs142512579 | p.D432N | C | C | C/C |
| chr1:97573785 | rs186169810 | p.F438L | A | A | Missing |
| chr1:97549735 | rs72975710 | p.A450V | G | A | G/A |
| chr1:97549726 | rs144395748 | p.P453R | G | G | G/G |
| chr1:97549713 | rs57918000 | p.N457N | G | G | Missing |
| chr1:97549681 | rs199549923 | p.T468N | G | G | Missing |
| chr1:97515923 | rs148994843 | p.V515I | C | C | Missing |
| chr1:97549609 | rs72549304 | p.S492L | G | G | Missing |
| chr1:97549600 | rs111858276 | p.D495G | T | T | Missing |
| chr1:97549565 | rs138391898 | p.V507I | C | C | Missing |
| chr1:97515889 | rs190951787 | p.T526S | G | G | Missing |
| chr1:97515865 | rs1801158 | p.S534N | C | C | Missing |
| chr1:97515851 | rs142619737 | p.G539R | C | C | Missing |
| chr1:97515839 | rs1801159 | p.I543V | T | T | Missing |
| chr1:97515787 | rs55886062 | p.I560S | A | A | Missing |
| chr1:97515784 | rs201615754 | p.R561L | C | C | Missing |
| chr1:97450190 | rs59086055 | p.R592W | G | G | Missing |
| chr1:97450189 | rs138616379 | p.R592Q | C | C | Missing |
| chr1:97450187 | rs145773863 | p.G593R | C | C | Missing |
| chr1:97450168 | rs147601618 | p.M599T | A | A | A/A |
| chr1:97450059 | rs3918289 | p.N635K | G | G | Missing |
| chr1:97450058 | rs3918290 | splicing defect | C | C | Missing |
| chr1:97382461 | rs55971861 | p.I636L | T | T | Missing |
| chr1:97373598 | rs137999090 | p.G674D | C | C | Missing |
| chr1:97306195 | rs145548112 | p.A721T | C | C | Missing |
| chr1:97305372 | rs146529561 | p.A729V | G | G | G/G |
| chr1:97305364 | rs1801160 | p.V732I | C | C | Missing |
| chr1:97305363 | rs60511679 | p.V732G | A | A | Missing |
| chr1:97305279 | rs112766203 | p.T760I | G | G | Missing |
| chr1:97234991 | rs56005131 | p.T768K | G | G | Missing |
| chr1:97234958 | rs199634007 | p.T779N | G | G | Missing |
| chr1:97193209 | rs200687447 | p.E828K | C | C | C/C |
| chr1:97193109 | rs60139309 | p.K861R | T | T | Missing |
| chr1:97098632 | rs201035051 | p.K875Q | T | T | Missing |
| chr1:97079077 | rs202144771 | p.L993F | G | G | Missing |
| chr1:97740400 | rs150385342 | p.A105T | C | C | Missing |
| chr1:97079076 | rs139459586 | p.L993R | A | A | Missing |
| chr1:97079071 | rs1801268 | p.V995F | C | C | Missing |
| chr1:97079005 | rs140114515 | p.V1017I | C | C | Missing |
| chr1:97078993 | rs148799944 | p.V1021L | C | C | Missing |
| chr1:97078987 | rs114096998 | p.P1023T | G | G | G/G |
| chr1:97883368 | rs150036960 | p.L16V | G | G | Missing |
| chr1:97883353 | rs72549310 | p.R21X | G | G | Missing |
| chr1:97883352 | rs80081766 | p.R21Q | C | C | Missing |
| chr1:97721650 | rs141462178 | p.M115V | T | T | Missing |
| chr1:97721542 | rs200562975 | p.N151D | T | T | T/T |
| chr1:97699535 | rs2297595 | p.M166V | T | T | Missing |
| chr1:97593322 | rs183385770 | p.D342N | C | C | C/C |
| chr1:97079121 | rs72547601 | p.H978R | T | T | Missing |
| chr1:97450068 | rs17376848 | p.F632F | A | A | Missing |
| chr1:97098616 | rs55674432 | p.G880V | C | C | C/C |
| chr1:97098599 | rs147545709 | p.R886C | G | G | Missing |
| chr1:97098598 | rs1801267 | p.R886H | C | C | Missing |
| chr1:97699430 | rs72549308 | p.S201R | T | T | Missing |
| chr1:97573919 | rs140602333 | p.R394W | G | G | G/G |
| chr1:97082391 | rs67376798 | p.D949V | T | T | Missing |
| chr1:97082365 | rs141044036 | p.K958E | T | T | Missing |
| chr1:97079139 | rs145529148 | p.Q972R | T | T | Missing |
| chr1:97079133 | rs72547602 | p.D974V | T | T | T/T |

### **NUDT15: \*1/\*1**

| Position | Variant | Effect on Protein | Definition of \*1 | Variant Call |
| --- | --- | --- | --- | --- |
| chr13:48037826 | rs777311140 | p.C28fs | del | Missing |
| chr13:48037784 | rs746071566 | p.del17\_18GV; p.V18\_V19insGV | GAGTCG(3) | Missing |
| chr13:48040982 | rs1457579126 | p.N74fs | A | Missing |
| chr13:48041104 | rs761191455 | p.E115fs | G | Missing |
| chr13:48037847 | rs766023281 | p.R34T | G | Missing |
| chr13:48037748 | rs769369441 | p.M1T | T | Missing |
| chr13:48037749 |  | p.M1I | G | Missing |
| chr13:48037798 | rs186364861 | p.V18I | G | Missing |
| chr13:48037849 |  | p.K35E | A | Missing |
| chr13:48037885 | rs1950545307 | p.G47R | G | Missing |
| chr13:48037902 | rs149436418 | p.F52L | C | C/C |
| chr13:48041113 | rs1368252918 | p.E118X | G | G/G |
| chr13:48045690 | rs768324690 | p.P129R | C | Missing |
| chr13:48045719 | rs116855232 | p.R139C | C | Missing |
| chr13:48045720 | rs147390019 | p.R139H | G | Missing |
| chr13:48045771 | rs139551410 | p.L156Q | T | Missing |
| chr13:48037834 | rs1202487323 | p.L30V | C | Missing |

### **SLCO1B1: \*20/\*30**

Please notice that SLCO1B1\*48, SLCO1B1\*49 are not considered in the current version, which could potentially have an impact on the results.

| Position | Variant | Effect on Protein | Definition of \*20 | Definition of \*30 | Variant Call |
| --- | --- | --- | --- | --- | --- |
| chr12:21172734 | rs139257324 | p.R57W | C | C | Missing |
| chr12:21172776 | rs373327528 | p.G71R | G | G | Missing |
| chr12:21172782 | rs56101265 | p.F73L | T | T | Missing |
| chr12:21174595 | rs56061388 | p.V82A | T | T | Missing |
| chr12:21176804 | rs2306283 | p.N130D | G | G | G/G |
| chr12:21176868 | rs2306282 | p.N151S | A | A | Missing |
| chr12:21176871 |  | p.R152L | G | G | Missing |
| chr12:21176879 | rs11045819 | p.P155T | C | C | Missing |
| chr12:21176883 | rs72559745 | p.E156G | A | A | Missing |
| chr12:21176898 | rs77271279 | splicing defect | G | G | Missing |
| chr12:21178612 | rs141467543 | p.Y173C | A | A | A/A |
| chr12:21178615 | rs4149056 | p.V174A | T | T | Missing |
| chr12:21178957 | rs79135870 | p.I222V | A | G | A/G |
| chr12:21196951 | rs11045852 | p.I245V | A | A | Missing |
| chr12:21196975 | rs183501729 | p.R253X | C | C | Missing |
| chr12:21196976 | rs11045853 | p.R253Q | G | G | Missing |
| chr12:21200544 | rs72559747 | p.P336R | C | C | Missing |
| chr12:21200595 | rs55901008 | p.I353T | T | T | Missing |
| chr12:21202553 | rs1228465562 | p.F400V | T | T | Missing |
| chr12:21202555 | rs59113707 | p.F400L | C | C | Missing |
| chr12:21202649 | rs56387224 | p.N432D | A | A | Missing |
| chr12:21202664 | rs142965323 | p.G437R | G | G | Missing |
| chr12:21205921 | rs72559748 | p.D462G | A | A | Missing |
| chr12:21205999 | rs59502379 | p.G488A | G | G | Missing |
| chr12:21206031 | rs74064213 | p.I499V | A | A | A/A |
| chr12:21222355 | rs71581941 | p.R580X | C | C | C/C |
| chr12:21239042 | rs34671512 | p.L643F | C | A | A/C |
| chr12:21239077 | rs56199088 | p.D655G | A | A | Missing |
| chr12:21239113 | rs55737008 | p.E667G | A | A | A/A |
| chr12:21239145 | rs200995543 | p.H678Y | C | C | C/C |
| chr12:21239158 | rs140790673 | p.S682F | C | C | Missing |

### **TPMT: \*1/\*1**

| Position | Variant | Effect on Protein | Definition of \*1 | Variant Call |
| --- | --- | --- | --- | --- |
| chr6:18149127 | rs9333569 | p.M1V | T | Missing |
| chr6:18149126 | rs267607275 | p.M1T | A | A/A |
| chr6:18149045 | rs72552742 | p.E28V | T | Missing |
| chr6:18149032 | rs759836180 | p.K32KfsX58 | del | Missing |
| chr6:18149022 | rs750424422 | p.G36S | C | Missing |
| chr6:18149004 |  | p.Q42E | G | Missing |
| chr6:18147910 | rs72552740 | p.L49S | A | Missing |
| chr6:18147856 |  | p.F67S | A | Missing |
| chr6:18147851 | rs200591577 | p.L69V | G | Missing |
| chr6:18147845 | rs777686348 | p.G71R | C | Missing |
| chr6:18147838 | rs281874771 | p.A73V | G | G/G |
| chr6:18143724 | rs1800462 | p.A80P | C | Missing |
| chr6:18143718 | rs111901354 | p.R82W | G | Missing |
| chr6:18143700 | rs753545734 | p.G88S | C | C/C |
| chr6:18143643 |  | p.Y107D | A | Missing |
| chr6:18143622 | rs115106679 | p.E114K | C | Missing |
| chr6:18143613 |  | p.G117R | C | Missing |
| chr6:18143606 | rs151149760 | p.K119T | T | T/T |
| chr6:18143597 |  | p.K122T | T | T/T |
| chr6:18139710 | rs200220210 | p.S125L | G | Missing |
| chr6:18139689 | rs72552738 | p.C132Y | C | Missing |
| chr6:18139027 | rs72552737 | p.G144R | C | Missing |
| chr6:18138997 | rs1800460 | p.A154T | C | Missing |
| chr6:18138970 | rs112339338 | p.R163C | G | Missing |
| chr6:18133890 | rs9333570 |  | C | Missing |
| chr6:18133887 | rs201695576 | p.Y166C | T | Missing |
| chr6:18133884 | rs74423290 | p.A167G | G | Missing |
| chr6:18133870 | rs772832951 | p.S172P | A | Missing |
| chr6:18133847 | rs6921269 | p.Q179H | C | Missing |
| chr6:18133845 | rs75543815 | p.Y180F | T | Missing |
| chr6:18132163 |  | p.V199I | C | Missing |
| chr6:18132147 | rs79901429 | p.I204T | A | Missing |
| chr6:18132136 | rs72556347 | p.F208L | A | Missing |
| chr6:18130781 | rs1800584 |  | C | Missing |
| chr6:18130772 | rs377085266 | p.C212R | A | Missing |
| chr6:18130762 | rs56161402 | p.R215H | C | Missing |
| chr6:18130758 | rs398122996 | p.C216X | A | Missing |
| chr6:18130729 | rs139392616 | p.R226Q | C | Missing |
| chr6:18130725 | rs72552736 | p.H227Q | A | Missing |
| chr6:18130694 | rs150900439 | p.K238E | T | Missing |
| chr6:18130687 | rs1142345 | p.Y240C; p.Y240S | T | Missing |
| chr6:18143728 | rs1256618794 | p.W78C | C | Missing |
| chr6:18138969 | rs144041067 | p.R163H; p.R163P | C | Missing |

### **UGT1A1: \*80+\*28/\*80+\*28**

| Position | Variant | Effect on Protein | Definition of \*80+\*28 | Variant Call |
| --- | --- | --- | --- | --- |
| chr2:233759924 | rs887829 | 5' Flanking | T | T/T |
| chr2:233760235 | rs3064744 | 5' Flanking | TA(8) | CAT/CAT |
| chr2:233760498 | rs4148323 | p.G71R | G | Missing |
| chr2:233760973 | rs35350960 | p.P229Q | C | Missing |

### **Single-variant allele**

Single-variant alleles constitute diplotypes that do not involve the judgment of multiple variants and the corresponding genes generally have not yet been standardized by a nomenclature committee, such as rs9923231 for VKORC1.

| Gene | Variant | Variant Call |
| --- | --- | --- |
| - | rs12777823 | Missing |
| ABCG2 | rs2231142 | Missing |
| ACE | rs1799752 | A/A |
| ADD1 | rs4961 | Missing |
| ADRB2 | rs1042713 | Missing |
| ALDH2 | rs671 | Missing |
| APOE | rs7412 | Missing |
| ATIC | rs4673993 | Missing |
| CACNA1S | rs1800559 | Missing |
| CACNA1S | rs772226819 | Missing |
| CES1 | rs71647871 | Missing |
| CFTR | rs113993958 | Missing |
| CFTR | rs113993960 | Missing |
| CFTR | rs115545701 | Missing |
| CFTR | rs11971167 | Missing |
| CFTR | rs121908752 | Missing |
| CFTR | rs121908753 | Missing |
| CFTR | rs121908755 | Missing |
| CFTR | rs121908757 | Missing |
| CFTR | rs121909005 | Missing |
| CFTR | rs121909013 | Missing |
| CFTR | rs121909020 | G/G |
| CFTR | rs121909041 | T/T |
| CFTR | rs141033578 | Missing |
| CFTR | rs150212784 | Missing |
| CFTR | rs186045772 | T/T |
| CFTR | rs193922525 | Missing |
| CFTR | rs199826652 | Missing |
| CFTR | rs200321110 | Missing |
| CFTR | rs202179988 | Missing |
| CFTR | rs267606723 | G/G |
| CFTR | rs368505753 | Missing |
| CFTR | rs397508256 | Missing |
| CFTR | rs397508288 | Missing |
| CFTR | rs397508387 | Missing |
| CFTR | rs397508442 | Missing |
| CFTR | rs397508513 | A/A |
| CFTR | rs397508537 | Missing |
| CFTR | rs397508759 | G/G |
| CFTR | rs397508761 | Missing |
| CFTR | rs74503330 | Missing |
| CFTR | rs74551128 | Missing |
| CFTR | rs75039782 | Missing |
| CFTR | rs75527207 | Missing |
| CFTR | rs75541969 | Missing |
| CFTR | rs76151804 | Missing |
| CFTR | rs77834169 | Missing |
| CFTR | rs77932196 | Missing |
| CFTR | rs78655421 | Missing |
| CFTR | rs78769542 | Missing |
| CFTR | rs80224560 | Missing |
| CFTR | rs80282562 | Missing |
| CHRNA5 | rs16969968 | Missing |
| CYP2B6 | rs28399499 | T/C |
| CYP2B6 | rs3745274 | Missing |
| CYP3A4 | rs4646437 | G/A |
| CYP4F2 | rs2108622 | Missing |
| DPYD | rs115232898 | T/T |
| DPYD | rs148994843 | Missing |
| DPYD | rs17376848 | Missing |
| DPYD | rs1801158 | Missing |
| DPYD | rs1801159 | Missing |
| DPYD | rs1801160 | Missing |
| DPYD | rs1801265 | A/G |
| DPYD | rs1801266 | G/G |
| DPYD | rs1801268 | Missing |
| DPYD | rs2297595 | Missing |
| DPYD | rs3918290 | Missing |
| DPYD | rs55886062 | Missing |
| DPYD | rs56005131 | Missing |
| DPYD | rs56038477 | Missing |
| DPYD | rs59086055 | Missing |
| DPYD | rs67376798 | Missing |
| DPYD | rs72549303 | Missing |
| DPYD | rs72549306 | Missing |
| DPYD | rs72549309 | Missing |
| DPYD | rs75017182 | Missing |
| DPYD | rs78060119 | Missing |
| EGFR | rs121434568 | Missing |
| EGFR | rs121434569 | Missing |
| F5 | rs6025 | Missing |
| FCGR3A | rs396991 | Missing |
| HLA-A | \*15:02 | Missing |
| HLA-A | \*31:01 | Missing |
| HLA-A | \*31:01:02 | Zero copy |
| HLA-A | \*33:03 | Missing |
| HLA-B | \*13:01:01 | Zero copy |
| HLA-B | \*15:02 | Missing |
| HLA-B | \*15:02:01 | Zero copy |
| HLA-B | \*15:11 | Missing |
| HLA-B | \*15:11:01 | Zero copy |
| HLA-B | \*31:01 | Missing |
| HLA-B | \*38:02:01 | Zero copy |
| HLA-B | \*40:01:01 | Zero copy |
| HLA-B | \*57:01 | Missing |
| HLA-B | \*57:01:01 | Zero copy |
| HLA-B | \*58:01 | Missing |
| HLA-B | \*59:01:01:01 | Zero copy |
| HLA-C | \*01:02:01 | Zero copy |
| HLA-C | \*03:02 | Missing |
| HLA-C | \*04:01:01:01 | Zero copy |
| HLA-C | \*06:02:01:01 | Zero copy |
| HLA-C | \*07:27:01 | Missing |
| HLA-C | \*08:01 | Missing |
| HLA-DPB1 | \*03:01:01 | Missing |
| HLA-DRB1 | \*01:01:01 | Zero copy |
| IFNL3 | rs11881222 | G/G |
| IFNL3 | rs12979860 | T/T |
| IFNL3 | rs8099917 | Missing |
| IFNL4 | rs11322783 | Missing |
| IFNL4 | rs12979860 | T/T |
| ITPA | rs1127354 | Missing |
| ITPA | rs7270101 | Missing |
| KIF6 | rs20455 | A/G |
| MT-ND1 | rs267606617 | Missing |
| MT-RNR1 | rs267606617 | Missing |
| MT-RNR1 | rs267606618 | Missing |
| MT-RNR1 | rs267606619 | Missing |
| MTHFR | rs1801133 | G/A |
| NUDT15 | rs116855232 | Missing |
| RARG | rs2229774 | Missing |
| RYR1 | rs112563513 | Missing |
| RYR1 | rs118192116 | Missing |
| RYR1 | rs118192122 | Missing |
| RYR1 | rs118192124 | Missing |
| RYR1 | rs118192161 | Missing |
| RYR1 | rs118192162 | Missing |
| RYR1 | rs118192163 | Missing |
| RYR1 | rs118192167 | Missing |
| RYR1 | rs118192168 | Missing |
| RYR1 | rs118192170 | Missing |
| RYR1 | rs118192172 | Missing |
| RYR1 | rs118192175 | Missing |
| RYR1 | rs118192176 | Missing |
| RYR1 | rs118192177 | Missing |
| RYR1 | rs118192178 | Missing |
| RYR1 | rs121918592 | Missing |
| RYR1 | rs121918593 | Missing |
| RYR1 | rs121918594 | G/G |
| RYR1 | rs121918595 | Missing |
| RYR1 | rs121918596 | Missing |
| RYR1 | rs1801086 | Missing |
| RYR1 | rs193922747 | Missing |
| RYR1 | rs193922748 | Missing |
| RYR1 | rs193922753 | Missing |
| RYR1 | rs193922762 | Missing |
| RYR1 | rs193922764 | Missing |
| RYR1 | rs193922768 | Missing |
| RYR1 | rs193922770 | Missing |
| RYR1 | rs193922772 | G/G |
| RYR1 | rs193922802 | Missing |
| RYR1 | rs193922803 | Missing |
| RYR1 | rs193922807 | Missing |
| RYR1 | rs193922809 | G/G |
| RYR1 | rs193922816 | Missing |
| RYR1 | rs193922818 | G/G |
| RYR1 | rs193922832 | G/G |
| RYR1 | rs193922843 | Missing |
| RYR1 | rs193922876 | Missing |
| RYR1 | rs193922878 | Missing |
| RYR1 | rs28933396 | Missing |
| RYR1 | rs28933397 | Missing |
| RYR1 | rs63749869 | Missing |
| SCN1A | rs3812718 | C/T |
| SLC19A1 | rs1051266 | Missing |
| SLC28A3 | rs7853758 | G/A |
| SLCO1B1 | rs4149056 | Missing |
| TNF | rs1800629 | Missing |
| UGT1A1 | rs10929302 | A/A |
| VKORC1 | rs2359612 | A/G |
| VKORC1 | rs2884737 | Missing |
| VKORC1 | rs61742245 | Missing |
| VKORC1 | rs7294 | C/T |
| VKORC1 | rs8050894 | C/G |
| VKORC1 | rs9923231 | C/T |
| VKORC1 | rs9934438 | G/A |
| XPNPEP2 | rs3788853 | C/A |
| XRCC1 | rs25487 | T/C |

## **Phenotype Prediction**

For the clinically available drugs, PAnno integrates the effects of multiple diplotypes for each drug in terms of toxicity, dosage, efficacy, and metabolism. The predicted phenotypes are based on PharmGKB's high-confidence clinical annotations (evidence levels 1A, 1B, 2A, 2B) and are indicated as decreased, normal, and increased.

Drugs not further annotated due to "Avoid use": amitriptyline, antidepressants, clomipramine, doxepin, imipramine, trimipramine, venlafaxine.  
Drugs not included in clinical annotations used by PAnno: eliglustat, antidepressants, pimozide, thioguanine, brexpiprazole, fosphenytoin.

| Drug | Toxicity | Dosage | Efficacy | Metabolism |
| --- | --- | --- | --- | --- |
| azathioprine | ◎ Normal | ◎ Normal | - | - |
| capecitabine | ◎ Normal | - | - | - |
| celecoxib | - | - | - | ◎ Normal |
| clopidogrel | ⤋ Decreased | - | ⤋ Decreased | ⤊ Increased |
| codeine | - | - | - | ⤋ Decreased |
| desflurane | ⤋ Decreased | - | - | - |
| efavirenz | ⤊ Increased | - | - | ⤋ Decreased |
| enflurane | ⤋ Decreased | - | - | - |
| fluorouracil | ⤋ Decreased | - | - | - |
| flurbiprofen | - | - | - | ◎ Normal |
| fluvastatin | ◎ Normal | - | - | ◎ Normal |
| halothane | ⤋ Decreased | - | - | - |
| ibuprofen | - | - | - | ◎ Normal |
| irinotecan | ⤊ Increased | - | - | - |
| isoflurane | ⤋ Decreased | - | - | - |
| lansoprazole | - | - | - | ⤊ Increased |
| lornoxicam | - | - | - | ◎ Normal |
| meloxicam | - | - | - | ◎ Normal |
| mercaptopurine | ◎ Normal | ◎ Normal | - | - |
| methoxyflurane | ⤋ Decreased | - | - | - |
| metoprolol | - | - | - | ⤋ Decreased |
| omeprazole | - | - | - | ⤊ Increased |
| pantoprazole | - | - | - | ⤊ Increased |
| peginterferon alfa-2a | - | - | ⤋ Decreased | - |
| peginterferon alfa-2b | - | - | ⤋ Decreased | - |
| phenytoin | ◎ Normal | - | - | ◎ Normal |
| piroxicam | - | - | - | ◎ Normal |
| pravastatin | - | - | ⤊ Increased | - |
| ribavirin | - | - | ⤋ Decreased | - |
| sevoflurane | ⤋ Decreased | - | - | - |
| succinylcholine | ⤋ Decreased | - | - | - |
| tacrolimus | - | ⤋ Decreased | - | ⤋ Decreased |
| tamoxifen | - | - | - | ⤋ Decreased |
| tenoxicam | - | - | - | ◎ Normal |
| voriconazole | - | - | - | ⤊ Increased |
| warfarin | ⤊ Increased | ◎ Normal | ⤋ Decreased | - |

## **Clinical Annotation**

This section lists the clinical annotations on which the phenotype predictions are based.

| Drug | Category | Gene | Variant | Diplotype | Level | Phenotype | PharmGKB ID |  |
| --- | --- | --- | --- | --- | --- | --- | --- | --- |
| azathioprine | Toxicity |  |  |  |  |  |  |  |
|  |  | TPMT |  | \*1/\*1 | 1A | ◎ Normal | 1451237240 |  |
 Dosage || TPMT |  | \*1/\*1 | 1A | ◎ Normal | 1451237326 |
| capecitabine | Toxicity |
| DPYD | rs1801265 | A/G | 1A | ◎ Normal | 1451287240 |
| celecoxib | Metabolism |
| CYP2C9 |  | \*1/\*1 | 1A | ◎ Normal | 1451236700 |
| clopidogrel | Toxicity |
| CYP2C19 |  | \*1/\*17 | 1A | ⤋ Decreased | 1451282440 |
 Efficacy || CYP2C19 |  | \*1/\*17 | 1A | ⤋ Decreased | 1451282440 |
| CYP2C19 |  | \*1/\*17 | 1A | ⤋ Decreased | 1451282340 |
 Metabolism || CYP2C19 |  | \*1/\*17 | 1A | ⤊ Increased | 1043858794 |
| codeine | Metabolism |
| CYP2D6 |  | \*17/\*29 | 1A | ⤋ Decreased | 1183616718 |
| desflurane | Toxicity |
| RYR1 | rs121918594 | G/G | 1A | ⤋ Decreased | 1183705827 |
| RYR1 | rs193922772 | G/G | 1A | ⤋ Decreased | 1445400222 |
| RYR1 | rs193922809 | G/G | 1A | ⤋ Decreased | 1447673669 |
| RYR1 | rs193922818 | G/G | 1A | ⤋ Decreased | 1447673778 |
| RYR1 | rs193922832 | G/G | 1A | ⤋ Decreased | 1449310249 |
| efavirenz | Toxicity |
| CYP2B6 |  | \*1/\*18 | 1A | ⤊ Increased | 1451243980 |
 Metabolism || CYP2B6 |  | \*1/\*18 | 1A | ⤋ Decreased | 1184133833 |
| enflurane | Toxicity |
| RYR1 | rs121918594 | G/G | 1A | ⤋ Decreased | 1183705827 |
| RYR1 | rs193922772 | G/G | 1A | ⤋ Decreased | 1445400222 |
| RYR1 | rs193922809 | G/G | 1A | ⤋ Decreased | 1447673669 |
| RYR1 | rs193922818 | G/G | 1A | ⤋ Decreased | 1447673778 |
| RYR1 | rs193922832 | G/G | 1A | ⤋ Decreased | 1449310249 |
| fluorouracil | Toxicity |
| DPYD | rs115232898 | T/T | 1A | ⤋ Decreased | 1183703784 |
| DPYD | rs1801265 | A/G | 1A | ◎ Normal | 981201981 |
 Other || DPYD | rs115232898 | T/T | 1A | ⤊ Increased | 1451274140 |
| DPYD | rs1801266 | G/G | 1A | ⤊ Increased | 1447989706 |
| flurbiprofen | Metabolism |
| CYP2C9 |  | \*1/\*1 | 1A | ◎ Normal | 1444842106 |
| fluvastatin | Toxicity |
| CYP2C9 |  | \*1/\*1 | 1A | ◎ Normal | 1451678600 |
 Metabolism || CYP2C9 |  | \*1/\*1 | 1A | ◎ Normal | 1451666740 |
| halothane | Toxicity |
| RYR1 | rs121918594 | G/G | 1A | ⤋ Decreased | 1183705827 |
| RYR1 | rs193922772 | G/G | 1A | ⤋ Decreased | 1445400222 |
| RYR1 | rs193922809 | G/G | 1A | ⤋ Decreased | 1447673669 |
| RYR1 | rs193922818 | G/G | 1A | ⤋ Decreased | 1447673778 |
| RYR1 | rs193922832 | G/G | 1A | ⤋ Decreased | 1449310249 |
| ibuprofen | Metabolism |
| CYP2C9 |  | \*1/\*1 | 1A | ◎ Normal | 1451092720 |
| irinotecan | Toxicity |
| UGT1A1 | rs10929302 | A/A | 2A | ⤊ Increased | 982030836 |
| isoflurane | Toxicity |
| RYR1 | rs121918594 | G/G | 1A | ⤋ Decreased | 1183705827 |
| RYR1 | rs193922772 | G/G | 1A | ⤋ Decreased | 1445400222 |
| RYR1 | rs193922809 | G/G | 1A | ⤋ Decreased | 1447673669 |
| RYR1 | rs193922818 | G/G | 1A | ⤋ Decreased | 1447673778 |
| RYR1 | rs193922832 | G/G | 1A | ⤋ Decreased | 1449310249 |
| lansoprazole | Metabolism |
| CYP2C19 |  | \*1/\*17 | 1A | ⤊ Increased | 1450806874 |
| lornoxicam | Metabolism |
| CYP2C9 |  | \*1/\*1 | 1A | ◎ Normal | 1183703296 |
| meloxicam | Metabolism |
| CYP2C9 |  | \*1/\*1 | 1A | ◎ Normal | 1451092677 |
| mercaptopurine | Toxicity |
| NUDT15 |  | \*1/\*1 | 1A | ◎ Normal | 1451448820 |
| TPMT |  | \*1/\*1 | 1A | ◎ Normal | 1184648909 |
 Dosage || NUDT15 |  | \*1/\*1 | 1A | ◎ Normal | 1448635217 |
| TPMT |  | \*1/\*1 | 1A | ◎ Normal | 1451237200 |
| methoxyflurane | Toxicity |
| RYR1 | rs121918594 | G/G | 1A | ⤋ Decreased | 1183705827 |
| RYR1 | rs193922772 | G/G | 1A | ⤋ Decreased | 1445400222 |
| RYR1 | rs193922809 | G/G | 1A | ⤋ Decreased | 1447673669 |
| RYR1 | rs193922818 | G/G | 1A | ⤋ Decreased | 1447673778 |
| RYR1 | rs193922832 | G/G | 1A | ⤋ Decreased | 1449310249 |
| metoprolol | Metabolism |
| CYP2D6 |  | \*17/\*29 | 1A | ⤋ Decreased | 1447681932 |
| omeprazole | Metabolism |
| CYP2C19 |  | \*1/\*17 | 1A | ⤊ Increased | 1183630065 |
| pantoprazole | Metabolism |
| CYP2C19 |  | \*1/\*17 | 1A | ⤊ Increased | 1183624491 |
| peginterferon alfa-2a | Efficacy |
| IFNL4 | rs12979860 | T/T | 1A | ⤋ Decreased | 1183888969 |
| IFNL4 | rs12979860 | T/T | 1A | ⤋ Decreased | 827862764 |
| IFNL4 | rs12979860 | T/T | 1A | ⤋ Decreased | 1183680546 |
| IFNL3 | rs11881222 | G/G | 2A | ⤋ Decreased | 1448102439 |
| peginterferon alfa-2b | Efficacy |
| IFNL4 | rs12979860 | T/T | 1A | ⤋ Decreased | 827862764 |
| IFNL4 | rs12979860 | T/T | 1A | ⤋ Decreased | 1183888969 |
| IFNL4 | rs12979860 | T/T | 1A | ⤋ Decreased | 1183680546 |
| IFNL3 | rs11881222 | G/G | 2A | ⤋ Decreased | 1448102439 |
| phenytoin | Toxicity |
| CYP2C9 |  | \*1/\*1 | 1A | ◎ Normal | 981238501 |
 Metabolism || CYP2C9 |  | \*1/\*1 | 1A | ◎ Normal | 982047500 |
| piroxicam | Metabolism |
| CYP2C9 |  | \*1/\*1 | 1A | ◎ Normal | 1451092541 |
| pravastatin | Efficacy |
| KIF6 | rs20455 | A/G | 2B | ⤊ Increased | 655384621 |
| ribavirin | Efficacy |
| IFNL4 | rs12979860 | T/T | 1A | ⤋ Decreased | 827862764 |
| IFNL4 | rs12979860 | T/T | 1A | ⤋ Decreased | 1183888969 |
| IFNL4 | rs12979860 | T/T | 1A | ⤋ Decreased | 1183680546 |
| IFNL3 | rs11881222 | G/G | 2A | ⤋ Decreased | 1448102439 |
| sevoflurane | Toxicity |
| RYR1 | rs121918594 | G/G | 1A | ⤋ Decreased | 1183705827 |
| RYR1 | rs193922772 | G/G | 1A | ⤋ Decreased | 1445400222 |
| RYR1 | rs193922809 | G/G | 1A | ⤋ Decreased | 1447673669 |
| RYR1 | rs193922818 | G/G | 1A | ⤋ Decreased | 1447673778 |
| RYR1 | rs193922832 | G/G | 1A | ⤋ Decreased | 1449310249 |
| succinylcholine | Toxicity |
| RYR1 | rs121918594 | G/G | 1A | ⤋ Decreased | 1183705827 |
| RYR1 | rs193922772 | G/G | 1A | ⤋ Decreased | 1445400222 |
| RYR1 | rs193922809 | G/G | 1A | ⤋ Decreased | 1447673669 |
| RYR1 | rs193922818 | G/G | 1A | ⤋ Decreased | 1447673778 |
| RYR1 | rs193922832 | G/G | 1A | ⤋ Decreased | 1449310249 |
| tacrolimus | Dosage |
| CYP3A5 |  | \*1/\*3 | 1A | ⤋ Decreased | 981203719 |
| CYP3A5 |  | \*1/\*3 | 2A | ⤋ Decreased | 1451243216 |
| CYP3A5 |  | \*1/\*3 | 2A | ⤋ Decreased | 1451241700 |
 Metabolism || CYP3A5 |  | \*1/\*3 | 1A | ⤋ Decreased | 1451214480 |
| CYP3A4 |  | \*1/\*36 | 1B | ⤊ Increased | 1183689931 |
| CYP3A5 |  | \*1/\*3 | 1B | ⤋ Decreased | 1451241780 |
| CYP3A5 |  | \*1/\*3 | 1B | ⤋ Decreased | 1184999911 |
| CYP3A4 | rs4646437 | G/A | 2A | ◎ Normal | 1444686843 |
| tamoxifen | Metabolism |
| CYP2D6 |  | \*17/\*29 | 1A | ⤋ Decreased | 982029857 |
| tenoxicam | Metabolism |
| CYP2C9 |  | \*1/\*1 | 1A | ◎ Normal | 1451092460 |
| voriconazole | Metabolism |
| CYP2C19 |  | \*1/\*17 | 1A | ⤊ Increased | 1183689217 |
| warfarin | Toxicity |
| CYP2C9 |  | \*1/\*1 | 1A | ◎ Normal | 1447672988 |
| CYP2C9 |  | \*1/\*1 | 1A | ◎ Normal | 1447672658 |
| VKORC1 | rs9923231 | C/T | 1B | ⤊ Increased | 1447673005 |
| VKORC1 | rs9923231 | C/T | 2A | ⤊ Increased | 1449269910 |
 Dosage || CYP2C9 |  | \*1/\*1 | 1A | ◎ Normal | 981238341 |
| VKORC1 | rs9923231 | C/T | 1A | ⤋ Decreased | 655385012 |
| VKORC1 | rs2359612 | A/G | 1B | ⤋ Decreased | 655385024 |
| VKORC1 | rs7294 | C/T | 1B | ⤊ Increased | 655384733 |
| VKORC1 | rs8050894 | C/G | 1B | ⤋ Decreased | 655385028 |
| VKORC1 | rs9934438 | G/A | 1B | ⤋ Decreased | 655385392 |
 Efficacy || CYP2C9 |  | \*1/\*1 | 2A | ◎ Normal | 1447672600 |
| VKORC1 | rs9923231 | C/T | 2A | ⤋ Decreased | 1447672998 |
| VKORC1 | rs9923231 | C/T | 2A | ⤋ Decreased | 1447673015 |

## **About**

The report incorporates analyses of peer-reviewed studies and other publicly available information identified by PAnno by State Key Laboratory of Genetic Engineering from the School of Life Sciences and Human Phenome Institute, Fudan University, Shanghai, China. These analyses and information may include associations between a molecular alteration (or lack of alteration) and one or more drugs with potential clinical benefit (or potential lack of clinical benefit), including drug candidates that are being studied in clinical research.  
*Note:* A finding of biomarker alteration does not necessarily indicate pharmacologic effectiveness (or lack thereof) of any drug or treatment regimen; a finding of no biomarker alteration does not necessarily indicate lack of pharmacologic effectiveness (or effectiveness) of any drug or treatment regimen.  
*No Guarantee of Clinical Benefit:* This Report makes no promises or guarantees that a particular drug will be effective in the treatment of disease in any patient. This report also makes no promises or guarantees that a drug with a potential lack of clinical benefit will provide no clinical benefit.  
*Treatment Decisions are Responsibility of Physician:* Drugs referenced in this report may not be suitable for a particular patient. The selection of any, all, or none of the drugs associated with potential clinical benefit (or potential lack of clinical benefit) resides entirely within the discretion of the treating physician. Indeed, the information in this report must be considered in conjunction with all other relevant information regarding a particular patient, before the patient's treating physician recommends a course of treatment. Decisions on patient care and treatment must be based on the independent medical judgment of the treating physician, taking into consideration all applicable information concerning the patient's condition, such as patient and family history, physical examinations, information from other diagnostic tests, and patient preferences, following the standard of care in a given community. A treating physician's decisions should not be based on a single test, such as this test or the information contained in this report.  
When using results obtained from PAnno, you agree to cite PAnno.

**PAnno v0.3.1**
- Written by Yaqing Liu, et al.,
available at GitHub,
PyPI, and Conda.
  
Copyright © 2021-2022 Center for Pharmacogenomics, Fudan University, China. All Rights Reserved.
