# Supplementary material for: PAnno: A pharmacogenomics annotation tool for clinical genomic testing
Source: Front Pharmacol. 2023 Jan 26;14:1008330. doi: 10.3389/fphar.2023.1008330 (PMC9909284; doi:10.3389/fphar.2023.1008330)
Supplement: Supplementary file 3 [file DataSheet1.ZIP › Supp.6 PAnno reports of four samples from different populations/NA19785.PAnno.html]

PAnno Report


# v0.3.1

- **Summary**
- **Prescribing Info**
- **Diplotype Detail**
- Multi-variant allele
- Single-variant allele
- **Phenotype Prediction**
- **Clinical Annotation**
- **About**

# 

An automated clinical pharmacogenomics annotation tool to report drug responses and prescribing recommendations by parsing the germline variants.

> Sample ID: NA19785  
> Biogeographic Group: Latino (LAT)  
> Report Time: Wed Dec 28 22:36:39 2022

**Disclaimer:** The PAnno report iterates as the release version changes. In the current release, you should only use it to evaluate whether PAnno will compile and run properly on your system. All information in the report is interpreted directly from the uploaded VCF file. Users recognize that they use it at their own risk.

## **Summary**

Drugs are classified to indicate whether the clinical guidelines recommend a prescribing change based on the given diplotypes. Original prescribing information was collected by PharmGKB, primarily from the Clinical Pharmacogenetics Implementation Consortium (CPIC), the Dutch Pharmacogenetics Working Group (DPWG), the Canadian Pharmacogenomics Network for Drug Safety (CPNDS), the French National Network of Pharmacogenetics (RNPGx).

**Avoid use**  

Avoidance of a drug is clearly stated in the prescribing recommendations for the given diplotype.

**Use with caution**  

Prescribing changes are recommended for the given diplotype, e.g., dose adjustment and alternative medication. In addition, prescribing recommendations that differ in specific populations or require consideration of multiple diplotypes are included in this category.

|  |  |  |  |  |  |  |
| --- | --- | --- | --- | --- | --- | --- |
| allopurinol | fosphenytoin | phenytoin | tacrolimus | warfarin |  |  |

**Routine use**  

There is no recommended prescribing change for the given diplotype.

|  |  |  |  |  |  |  |
| --- | --- | --- | --- | --- | --- | --- |
| amitriptyline | atomoxetine | atorvastatin | azathioprine | capecitabine | celecoxib | clomipramine |
| clopidogrel | codeine | desflurane | desipramine | dexlansoprazole | doxepin | efavirenz |
| enflurane | fluorouracil | flurbiprofen | fluvastatin | halothane | hydrocodone | ibuprofen |
| imipramine | isoflurane | lansoprazole | lornoxicam | lovastatin | meloxicam | mercaptopurine |
| methoxyflurane | nortriptyline | omeprazole | ondansetron | pantoprazole | peginterferon alfa-2a | peginterferon alfa-2b |
| piroxicam | pitavastatin | pravastatin | rasburicase | ribavirin | rosuvastatin | sevoflurane |
| simvastatin | succinylcholine | tamoxifen | tenoxicam | thioguanine | tramadol | trimipramine |
| tropisetron | voriconazole |  |  |  |  |  |

## **Prescribing Info**

### **allopurinol**

**Gene**: ABCG2    **Diplotype**: T/T    **Phenotype**: -

|  |  |
| --- | --- |
| **DPWG** | **Summary:** Patients with the GT or TT genotypes at rs2231142 should be given an increased dose of allopurinol. |
|  | **Recommendation:** Use 1.4 times the standard dose. This equates to a dose titration schedule of 100, 300, 400, 600 and 700 mg/day instead of the usual schedule of 100, 200, 300, 400 and 500 mg/day. |

### **amitriptyline**

**Gene**: CYP2C19    **Diplotype**: \*1/\*1    **Phenotype**: Normal Metabolizer

|  |  |
| --- | --- |
| **CPIC** | **Summary:** The CPIC Dosing Guideline update for amitriptyline recommends an alternative drug for CYP2D6 ultrarapid or poor metabolizers and CYP2C19 ultrarapid, rapid or poor metabolizers. If amitriptyline is warranted, consider a 50% dose reduction in CYP2D6 or CYP2C19 poor metabolizers. For CYP2D6 intermediate metabolizers, a 25% dose reduction should be considered. |
|  | **Recommendation:** Initiate therapy with recommended starting dose. |

**Gene**: CYP2D6    **Diplotype**: \*1/\*2    **Phenotype**: Normal Metabolizer

|  |  |
| --- | --- |
| **CPIC** | **Summary:** The CPIC Dosing Guideline update for amitriptyline recommends an alternative drug for CYP2D6 ultrarapid or poor metabolizers and CYP2C19 ultrarapid, rapid or poor metabolizers. If amitriptyline is warranted, consider a 50% dose reduction in CYP2D6 or CYP2C19 poor metabolizers. For CYP2D6 intermediate metabolizers, a 25% dose reduction should be considered. |
|  | **Recommendation:** Initiate therapy with recommended starting dose. |

### **atomoxetine**

**Gene**: CYP2D6    **Diplotype**: \*1/\*2    **Phenotype**: Normal Metabolizer

|  |  |
| --- | --- |
| **CPIC** | **Summary:** The CPIC Dosing Guideline for atomoxetine provides therapeutic recommendations for CYP2D6 ultrarapid, normal, intermediate, and poor metabolizer, which includes guidance for plasma drug concentration testing, as a means to estimate atomoxetine exposure, if no clinical response and in the absence of adverse events after 2 weeks of therapy. |
|  | **Recommendation:** Initiate with a dose of 0.5 mg/kg and increase to 1.2 mg/kg/day after 3 days. If no clinical response and in the absence of adverse events after 2 weeks, consider obtaining a peak plasma concentration (1 to 2 hours after dose administered). If <200 ng/ml, consider a proportional increase in dose to approach 400 ng/ml. |

### **atorvastatin**

**Gene**: SLCO1B1    **Diplotype**: \*1/\*37    **Phenotype**: Normal Function

|  |  |
| --- | --- |
| **CPIC** | **Summary:** Prescribe ≤20mg for patients with SLCO1B1 poor function phenotype and ≤40mg for patients with SLCO1B1 decreased or possible decreased phenotype as a starting dose. Adjust doses of atorvastatin based on disease-specific guidelines. Prescriber should be aware of possible increased risk for myopathy especially for 40mg dose. |
|  | **Recommendation:** Prescribe desired starting dose and adjust doses based on disease-specific guidelines. |

### **azathioprine**

**Gene**: TPMT    **Diplotype**: \*1/\*1    **Phenotype**: Normal Metabolizer

|  |  |
| --- | --- |
| **CPIC** | **Summary:** Consider an alternate agent or extreme dose reduction of azathioprine for patients who are TPMT or NUDT15 poor metabolizers. Start at 30-80% of target dose for patients who are TPMT or NUDT15 intermediate metabolizers. |
|  | **Recommendation:** Start with normal starting dose (e.g., 2-3 mg/kg/day) and adjust doses of azathioprine based on disease-specific guidelines. Allow 2 weeks to reach steady state after each dose adjustment. |

|  |  |
| --- | --- |
| **RNPGx** | **Summary:** Testing for TPMT genotype and phenotype is recommended for patients who are receiving azathioprine as an immunosuppressant. A dose reduction should be considered for patients with intermediate TPMT activity, while a greater dose reduction or selection of an alternate drug should be considered for patients with low TPMT activity. |
|  | **Recommendation:** Dose adjustment: Standard dose. Initial dose: Azathioprine 2.0-2.5 mg/kg/day. |

**Gene**: NUDT15    **Diplotype**: \*1/\*1    **Phenotype**: Normal Metabolizer

|  |  |
| --- | --- |
| **CPIC** | **Summary:** Consider an alternate agent or extreme dose reduction of azathioprine for patients who are TPMT or NUDT15 poor metabolizers. Start at 30-80% of target dose for patients who are TPMT or NUDT15 intermediate metabolizers. |
|  | **Recommendation:** Start with normal starting dose (e.g., 2-3 mg/kg/day) and adjust doses of azathioprine based on disease-specific guidelines. Allow 2 weeks to reach steady state after each dose adjustment. |

### **capecitabine**

**Gene**: DPYD    **Diplotype**: Reference/Reference    **Phenotype**: Normal Metabolizer

|  |  |
| --- | --- |
| **CPIC** | **Summary:** The CPIC Dosing Guideline for 5-fluorouracil and capecitabine recommends an alternative drug for patients who are DPYD poor metabolizers with an activity score of 0. In those who are poor metabolizers with an activity score of 0.5, an alternative drug is also recommended, but if this is not considered a suitable therapeutic option, 5-fluorouracil or capecitabine should be administered at a strongly reduced dose with early therapeutic drug monitoring. Patients who are intermediate metabolizers with an activity score of 1 or 1.5 should receive a dose reduction of 50%. Patients with the c.[2846A>T];[2846A>T] genotype may require a >50% dose reduction. |
|  | **Recommendation:** Based on genotype, there is no indication to change dose or therapy. Use label-recommended dosage and administration |

### **celecoxib**

**Gene**: CYP2C9    **Diplotype**: \*1/\*1    **Phenotype**: Normal Metabolizer

|  |  |
| --- | --- |
| **CPIC** | **Summary:** The CPIC Dosing Guideline for celecoxib, flurbiprofen, ibuprofen and lornoxicam recommends initiating therapy with 25-50% of the lowest recommended starting dose for CYP2C9 poor metabolizers and initiating therapy with lowest recommended starting dose for CYP2C9 intermediate metabolizers with activity score of 1. See full guideline for further details and supporting evidence. |
|  | **Recommendation:** Initiate therapy with recommended starting dose. In accordance with the prescribing information, use the lowest effective dosage for shortest duration consistent with individual patient treatment goals. |

### **clomipramine**

**Gene**: CYP2D6    **Diplotype**: \*1/\*2    **Phenotype**: Normal Metabolizer

|  |  |
| --- | --- |
| **CPIC** | **Summary:** Tricyclic antidepressants have comparable pharmacokinetic properties, it may be reasonable to apply the CPIC Dosing Guideline for amitriptyline and CYP2C19, CYP2D6 to other tricyclics including clomipramine. The CPIC Dosing Guideline update for amitriptyline recommends an alternative drug for CYP2D6 ultrarapid or poor metabolizers and CYP2C19 ultrarapid, rapid or poor metabolizers. If amitriptyline is warranted, consider a 50% dose reduction in CYP2D6 or CYP2C19 poor metabolizers. For CYP2D6 intermediate metabolizers, a 25% dose reduction should be considered. |
|  | **Recommendation:** Initiate therapy with recommended starting dose. |

**Gene**: CYP2C19    **Diplotype**: \*1/\*1    **Phenotype**: Normal Metabolizer

|  |  |
| --- | --- |
| **CPIC** | **Summary:** Tricyclic antidepressants have comparable pharmacokinetic properties, it may be reasonable to apply the CPIC Dosing Guideline for amitriptyline and CYP2C19, CYP2D6 to other tricyclics including clomipramine. The CPIC Dosing Guideline update for amitriptyline recommends an alternative drug for CYP2D6 ultrarapid or poor metabolizers and CYP2C19 ultrarapid, rapid or poor metabolizers. If amitriptyline is warranted, consider a 50% dose reduction in CYP2D6 or CYP2C19 poor metabolizers. For CYP2D6 intermediate metabolizers, a 25% dose reduction should be considered. |
|  | **Recommendation:** Initiate therapy with recommended starting dose. |

### **clopidogrel**

**Gene**: CYP2C19    **Diplotype**: \*1/\*1    **Phenotype**: Normal Metabolizer

|  |  |
| --- | --- |
| **CPIC** | **Summary:** The CPIC Dosing Guideline for clopidogrel recommends an alternative antiplatelet therapy for CYP2C19 poor or intermediate metabolizers (cardiovascular indications: prasugrel or ticagrelor if no contraindication; neurovascular indications: alternative P2Y12 inhibitor if clinically indicated and no contraindication.) |
|  | **Recommendation:** If considering clopidogrel, use at standard dose (75 mg/day) |

### **codeine**

**Gene**: CYP2D6    **Diplotype**: \*1/\*2    **Phenotype**: Normal Metabolizer

|  |  |
| --- | --- |
| **CPIC** | **Summary:** Alternate non-tramadol analgesics are recommended for CYP2D6 ultrarapid and poor metabolizers. A label recommended age- or weight-specific dose of codeine is warranted for CYP2D6 normal and intermediate metabolizers. |
|  | **Recommendation:** Use codeine label recommended age- or weight-specific dosing |

### **desflurane**

**Gene**: RYR1    **Diplotype**: Reference/Reference    **Phenotype**: Uncertain Susceptibility

|  |  |
| --- | --- |
| **CPIC** | **Summary:** The CPIC Dosing Guideline recommends that halogenated volatile anesthetics such as desflurane, enflurane, halothane, isoflurane, methoxyflurane, sevoflurane and the depolarizing muscle relaxants succinylcholine are relatively contraindicated in persons with malignant hyperthermia susceptibility (MHS). See full guideline for disclaimers, further details and supporting evidence. |
|  | **Recommendation:** These results do not eliminate the chance that this patient is susceptible to Malignant Hyperthermia. The genetic cause of about half of all MH survivors, with MH susceptibility confirmed by contracture test, remains unknown [Article:28902675]. |

**Gene**: CACNA1S    **Diplotype**: Reference/Reference    **Phenotype**: Uncertain Susceptibility

|  |  |
| --- | --- |
| **CPIC** | **Summary:** The CPIC Dosing Guideline recommends that halogenated volatile anesthetics such as desflurane, enflurane, halothane, isoflurane, methoxyflurane, sevoflurane and the depolarizing muscle relaxants succinylcholine are relatively contraindicated in persons with malignant hyperthermia susceptibility (MHS). See full guideline for disclaimers, further details and supporting evidence. |
|  | **Recommendation:** These results do not eliminate the chance that this patient is susceptible to Malignant Hyperthermia. The genetic cause of about half of all MH survivors, with MH susceptibility confirmed by contracture test, remains unknown [Article:28902675]. |

### **desipramine**

**Gene**: CYP2D6    **Diplotype**: \*1/\*2    **Phenotype**: Normal Metabolizer

|  |  |
| --- | --- |
| **CPIC** | **Summary:** Tricyclic antidepressants have comparable pharmacokinetic properties, it may be reasonable to apply the CPIC Dosing Guideline for amitriptyline/nortriptyline and CYP2C19, CYP2D6 to other tricyclics including desipramine. The CPIC Dosing Guideline update for nortriptyline recommends a 25% dose reduction for CYP2D6 intermediate metabolizers. For CYP2D6 ultrarapid or poor metabolizers, an alternative drug should be considered. If nortriptyline is warranted, consider a 50% dose reduction in CYP2D6 poor metabolizers. |
|  | **Recommendation:** Initiate therapy with recommended starting dose. |

### **dexlansoprazole**

**Gene**: CYP2C19    **Diplotype**: \*1/\*1    **Phenotype**: Normal Metabolizer

|  |  |
| --- | --- |
| **CPIC** | **Summary:** The CPIC Dosing Guideline recommendations for dexlansoprazole are based on the similarity in its metabolism and lansoprazole and extrapolated from the first-generation PPIs. The guideline recommends to increase the starting daily dose and to monitor efficacy in CYP2C19 ultrarapid metabolizer. For CYP2C19 rapid and normal metabolizers in the treatment of H. pylori infection and erosive esophagitis increasing the dose might be considered after initiation with the standard starting daily dose. The recommendations for intermediate and poor metabolizers for chronic therapy (>12 weeks) and efficacy achieved is to consider 50% reduction in daily dose. See full guideline for further details and supporting evidence. |
|  | **Recommendation:** Initiate standard starting daily dose. Consider increasing dose by 50-100% for the treatment of H. pylori infection and erosive esophagitis. Daily dose may be given in divided doses. Monitor for efficacy. |

### **doxepin**

**Gene**: CYP2C19    **Diplotype**: \*1/\*1    **Phenotype**: Normal Metabolizer

|  |  |
| --- | --- |
| **CPIC** | **Summary:** Tricyclic antidepressants have comparable pharmacokinetic properties, it may be reasonable to apply the CPIC Dosing Guideline for amitriptyline and CYP2C19, CYP2D6 to other tricyclics including doxepin. The CPIC Dosing Guideline update for amitriptyline recommends an alternative drug for CYP2D6 ultrarapid or poor metabolizers and CYP2C19 ultrarapid, rapid or poor metabolizers. If amitriptyline is warranted, consider a 50% dose reduction in CYP2D6 or CYP2C19 poor metabolizers. For CYP2D6 intermediate metabolizers, a 25% dose reduction should be considered. |
|  | **Recommendation:** Initiate therapy with recommended starting dose. |

**Gene**: CYP2D6    **Diplotype**: \*1/\*2    **Phenotype**: Normal Metabolizer

|  |  |
| --- | --- |
| **CPIC** | **Summary:** Tricyclic antidepressants have comparable pharmacokinetic properties, it may be reasonable to apply the CPIC Dosing Guideline for amitriptyline and CYP2C19, CYP2D6 to other tricyclics including doxepin. The CPIC Dosing Guideline update for amitriptyline recommends an alternative drug for CYP2D6 ultrarapid or poor metabolizers and CYP2C19 ultrarapid, rapid or poor metabolizers. If amitriptyline is warranted, consider a 50% dose reduction in CYP2D6 or CYP2C19 poor metabolizers. For CYP2D6 intermediate metabolizers, a 25% dose reduction should be considered. |
|  | **Recommendation:** Initiate therapy with recommended starting dose. |

### **efavirenz**

**Gene**: CYP2B6    **Diplotype**: \*1/\*5    **Phenotype**: Normal Metabolizer

|  |  |
| --- | --- |
| **CPIC** | **Summary:** Consider initiating efavirenz with a decreased dose of either 400 or 200 mg/day for patients who are CYP2B6 poor metabolizers. Consider initiating efavirenz with a decreased dose of 400 mg/day for patients who are CYP2B6 intermediate metabolizers. |
|  | **Recommendation:** Initiate efavirenz with standard dosing (600 mg/day) |

|  |  |
| --- | --- |
| **DPWG** | **Summary:** Adjust the initial efavirenz dose for patients with the CYP2B6 PM phenotype along with the consideration of age, weight and BMI and titrate the dose to plasma concentration if needed. For patients with the \*5/\*6, \*5/\*18 or other CYP2B6 IM genotypes, determine the efavirenz plasma concentration if side effects occur and reduce the dose if needed. |
|  | **Recommendation:** NO action is required for this gene-drug interaction |

### **enflurane**

**Gene**: CACNA1S    **Diplotype**: Reference/Reference    **Phenotype**: Uncertain Susceptibility

|  |  |
| --- | --- |
| **CPIC** | **Summary:** The CPIC Dosing Guideline recommends that halogenated volatile anesthetics such as desflurane, enflurane, halothane, isoflurane, methoxyflurane, sevoflurane and the depolarizing muscle relaxants succinylcholine are relatively contraindicated in persons with malignant hyperthermia susceptibility (MHS). See full guideline for disclaimers, further details and supporting evidence. |
|  | **Recommendation:** These results do not eliminate the chance that this patient is susceptible to Malignant Hyperthermia. The genetic cause of about half of all MH survivors, with MH susceptibility confirmed by contracture test, remains unknown [Article:28902675]. |

**Gene**: RYR1    **Diplotype**: Reference/Reference    **Phenotype**: Uncertain Susceptibility

|  |  |
| --- | --- |
| **CPIC** | **Summary:** The CPIC Dosing Guideline recommends that halogenated volatile anesthetics such as desflurane, enflurane, halothane, isoflurane, methoxyflurane, sevoflurane and the depolarizing muscle relaxants succinylcholine are relatively contraindicated in persons with malignant hyperthermia susceptibility (MHS). See full guideline for disclaimers, further details and supporting evidence. |
|  | **Recommendation:** These results do not eliminate the chance that this patient is susceptible to Malignant Hyperthermia. The genetic cause of about half of all MH survivors, with MH susceptibility confirmed by contracture test, remains unknown [Article:28902675]. |

### **fluorouracil**

**Gene**: DPYD    **Diplotype**: Reference/Reference    **Phenotype**: Normal Metabolizer

|  |  |
| --- | --- |
| **CPIC** | **Summary:** The CPIC Dosing Guideline for 5-fluorouracil and capecitabine recommends an alternative drug for patients who are DPYD poor metabolizers with an activity score of 0. In those who are poor metabolizers with an activity score of 0.5, an alternative drug is also recommended, but if this is not considered a suitable therapeutic option, 5-fluorouracil or capecitabine should be administered at a strongly reduced dose with early therapeutic drug monitoring. Patients who are intermediate metabolizers with an activity score of 1 or 1.5 should receive a dose reduction of 50%. Patients with the c.[2846A>T];[2846A>T] genotype may require a >50% dose reduction. |
|  | **Recommendation:** Based on genotype, there is no indication to change dose or therapy. Use label-recommended dosage and administration |

### **flurbiprofen**

**Gene**: CYP2C9    **Diplotype**: \*1/\*1    **Phenotype**: Normal Metabolizer

|  |  |
| --- | --- |
| **CPIC** | **Summary:** The CPIC Dosing Guideline for celecoxib, flurbiprofen, ibuprofen and lornoxicam recommends initiating therapy with 25-50% of the lowest recommended starting dose for CYP2C9 poor metabolizers and initiating therapy with lowest recommended starting dose for CYP2C9 intermediate metabolizers with activity score of 1. See full guideline for further details and supporting evidence. |
|  | **Recommendation:** Initiate therapy with recommended starting dose. In accordance with the prescribing information, use the lowest effective dosage for shortest duration consistent with individual patient treatment goals. |

### **fluvastatin**

**Gene**: SLCO1B1    **Diplotype**: \*1/\*37    **Phenotype**: Normal Function

|  |  |
| --- | --- |
| **CPIC** | **Summary:** CYP2C9 IMs should avoid fluvastatin doses greater than 40mg while CYP2C9 PMs should avoid doses greater than 20mg. If higher doses are required for desired efficacy, an alternative statin should be considered. Patients with SLCO1B1 poor function should also avoid fluvastatin doses greater than 40mg and and adjust doses of fluvastatin based on disease-specific guidelines. Patients with both SLCO1B1 poor function and CYP2C9 IM/PM should be prescribed an alternative statin depending on the desired potency. |
|  | **Recommendation:** Prescribe desired starting dose and adjust doses based on disease-specific guidelines. |

### **fosphenytoin**

**Gene**: CYP2C9    **Diplotype**: \*1/\*1    **Phenotype**: Normal Metabolizer

|  |  |
| --- | --- |
| **CPIC** | **Summary:** Phenytoin/fosphenytoin is contraindicated in individuals with the HLA-B\*15:02 variant allele ("HLA-B\*15:02-positive") due to significantly increased risk of phenytoin-induced cutaneous adverse reactions of Stevens-Johnson syndrome (SJS) and toxic epidermal necrolysis (TEN). Additionally, patients with the CYP2C9 poor metabolizer phenotype or with a CYP2C9 activity score of 1 may require reduced doses of phenytoin/fosphenytoin. |
|  | **Recommendation:** No adjustments needed from typical dosing strategies. Subsequent doses should be adjusted according to therapeutic drug monitoring, response, and side effects. An HLA-B\*15:02 negative test does not eliminate the risk of phenytoin-induced SJS/TEN, and patients should be carefully monitored according to standard practice. |

### **halothane**

**Gene**: CACNA1S    **Diplotype**: Reference/Reference    **Phenotype**: Uncertain Susceptibility

|  |  |
| --- | --- |
| **CPIC** | **Summary:** The CPIC Dosing Guideline recommends that halogenated volatile anesthetics such as desflurane, enflurane, halothane, isoflurane, methoxyflurane, sevoflurane and the depolarizing muscle relaxants succinylcholine are relatively contraindicated in persons with malignant hyperthermia susceptibility (MHS). See full guideline for disclaimers, further details and supporting evidence. |
|  | **Recommendation:** These results do not eliminate the chance that this patient is susceptible to Malignant Hyperthermia. The genetic cause of about half of all MH survivors, with MH susceptibility confirmed by contracture test, remains unknown [Article:28902675]. |

**Gene**: RYR1    **Diplotype**: Reference/Reference    **Phenotype**: Uncertain Susceptibility

|  |  |
| --- | --- |
| **CPIC** | **Summary:** The CPIC Dosing Guideline recommends that halogenated volatile anesthetics such as desflurane, enflurane, halothane, isoflurane, methoxyflurane, sevoflurane and the depolarizing muscle relaxants succinylcholine are relatively contraindicated in persons with malignant hyperthermia susceptibility (MHS). See full guideline for disclaimers, further details and supporting evidence. |
|  | **Recommendation:** These results do not eliminate the chance that this patient is susceptible to Malignant Hyperthermia. The genetic cause of about half of all MH survivors, with MH susceptibility confirmed by contracture test, remains unknown [Article:28902675]. |

### **hydrocodone**

**Gene**: CYP2D6    **Diplotype**: \*1/\*2    **Phenotype**: Normal Metabolizer

|  |  |
| --- | --- |
| **CPIC** | **Summary:** CYP2D6 intermediate and poor metabolizers should initiate hydrocodone therapy using the label recommended age- or weight-specific dosing. However, if there is no response to hydrocodone in these patients, an alternative analgesic should be considered. |
|  | **Recommendation:** Use hydrocodone label recommended age- or weight-specific dosing |

### **ibuprofen**

**Gene**: CYP2C9    **Diplotype**: \*1/\*1    **Phenotype**: Normal Metabolizer

|  |  |
| --- | --- |
| **CPIC** | **Summary:** The CPIC Dosing Guideline for celecoxib, flurbiprofen, ibuprofen and lornoxicam recommends initiating therapy with 25-50% of the lowest recommended starting dose for CYP2C9 poor metabolizers and initiating therapy with lowest recommended starting dose for CYP2C9 intermediate metabolizers with activity score of 1. See full guideline for further details and supporting evidence. |
|  | **Recommendation:** Initiate therapy with recommended starting dose. In accordance with the prescribing information, use the lowest effective dosage for shortest duration consistent with individual patient treatment goals. |

### **imipramine**

**Gene**: CYP2C19    **Diplotype**: \*1/\*1    **Phenotype**: Normal Metabolizer

|  |  |
| --- | --- |
| **CPIC** | **Summary:** Tricyclic antidepressants have comparable pharmacokinetic properties, it may be reasonable to apply the CPIC Dosing Guideline for amitriptyline and CYP2C19, CYP2D6 to other tricyclics including imipramine. The CPIC Dosing Guideline update for amitriptyline recommends an alternative drug for CYP2D6 ultrarapid or poor metabolizers and CYP2C19 ultrarapid, rapid or poor metabolizers. If amitriptyline is warranted, consider a 50% dose reduction in CYP2D6 or CYP2C19 poor metabolizers. For CYP2D6 intermediate metabolizers, a 25% dose reduction should be considered. |
|  | **Recommendation:** Initiate therapy with recommended starting dose. |

**Gene**: CYP2D6    **Diplotype**: \*1/\*2    **Phenotype**: Normal Metabolizer

|  |  |
| --- | --- |
| **CPIC** | **Summary:** Tricyclic antidepressants have comparable pharmacokinetic properties, it may be reasonable to apply the CPIC Dosing Guideline for amitriptyline and CYP2C19, CYP2D6 to other tricyclics including imipramine. The CPIC Dosing Guideline update for amitriptyline recommends an alternative drug for CYP2D6 ultrarapid or poor metabolizers and CYP2C19 ultrarapid, rapid or poor metabolizers. If amitriptyline is warranted, consider a 50% dose reduction in CYP2D6 or CYP2C19 poor metabolizers. For CYP2D6 intermediate metabolizers, a 25% dose reduction should be considered. |
|  | **Recommendation:** Initiate therapy with recommended starting dose. |

### **isoflurane**

**Gene**: CACNA1S    **Diplotype**: Reference/Reference    **Phenotype**: Uncertain Susceptibility

|  |  |
| --- | --- |
| **CPIC** | **Summary:** The CPIC Dosing Guideline recommends that halogenated volatile anesthetics such as desflurane, enflurane, halothane, isoflurane, methoxyflurane, sevoflurane and the depolarizing muscle relaxants succinylcholine are relatively contraindicated in persons with malignant hyperthermia susceptibility (MHS). See full guideline for disclaimers, further details and supporting evidence. |
|  | **Recommendation:** These results do not eliminate the chance that this patient is susceptible to Malignant Hyperthermia. The genetic cause of about half of all MH survivors, with MH susceptibility confirmed by contracture test, remains unknown [Article:28902675]. |

**Gene**: RYR1    **Diplotype**: Reference/Reference    **Phenotype**: Uncertain Susceptibility

|  |  |
| --- | --- |
| **CPIC** | **Summary:** The CPIC Dosing Guideline recommends that halogenated volatile anesthetics such as desflurane, enflurane, halothane, isoflurane, methoxyflurane, sevoflurane and the depolarizing muscle relaxants succinylcholine are relatively contraindicated in persons with malignant hyperthermia susceptibility (MHS). See full guideline for disclaimers, further details and supporting evidence. |
|  | **Recommendation:** These results do not eliminate the chance that this patient is susceptible to Malignant Hyperthermia. The genetic cause of about half of all MH survivors, with MH susceptibility confirmed by contracture test, remains unknown [Article:28902675]. |

### **lansoprazole**

**Gene**: CYP2C19    **Diplotype**: \*1/\*1    **Phenotype**: Normal Metabolizer

|  |  |
| --- | --- |
| **CPIC** | **Summary:** The CPIC Dosing Guideline for omeprazole, lansoprazole, pantoprazole recommends to increase the starting daily dose and to monitor efficacy in CYP2C19 ultrarapid metabolizer. For CYP2C19 rapid and normal metabolizers in the treatment of H. pylori infection and erosive esophagitis increasing the dose might be considered after initiation with the standard starting daily dose. The recommendations for intermediate and poor metabolizer for chronic therapy (>12 weeks) and efficacy achieved is to consider 50% reduction in daily dose. See full guideline for further details and supporting evidence. |
|  | **Recommendation:** Initiate standard starting daily dose. Consider increasing dose by 50-100% for the treatment of H. pylori infection and erosive esophagitis. Daily dose may be given in divided doses. Monitor for efficacy. |

### **lornoxicam**

**Gene**: CYP2C9    **Diplotype**: \*1/\*1    **Phenotype**: Normal Metabolizer

|  |  |
| --- | --- |
| **CPIC** | **Summary:** The CPIC Dosing Guideline for celecoxib, flurbiprofen, ibuprofen and lornoxicam recommends initiating therapy with 25-50% of the lowest recommended starting dose for CYP2C9 poor metabolizers and initiating therapy with lowest recommended starting dose for CYP2C9 intermediate metabolizers with activity score of 1. See full guideline for further details and supporting evidence. |
|  | **Recommendation:** Initiate therapy with recommended starting dose. In accordance with the prescribing information, use the lowest effective dosage for shortest duration consistent with individual patient treatment goals. |

### **lovastatin**

**Gene**: SLCO1B1    **Diplotype**: \*1/\*37    **Phenotype**: Normal Function

|  |  |
| --- | --- |
| **CPIC** | **Summary:** Prescribe an alternative statin depending on the desired potency for patients with SLCO1B1 decreased function, possible decreased function or poor function phenotype. If lovastatin therapy is warranted in patients with SLCO1B1 decreased or possible decreased phenotype, limit dose to <20mg/day. |
|  | **Recommendation:** Prescribe desired starting dose and adjust doses based on disease-specific guidelines. |

### **meloxicam**

**Gene**: CYP2C9    **Diplotype**: \*1/\*1    **Phenotype**: Normal Metabolizer

|  |  |
| --- | --- |
| **CPIC** | **Summary:** The CPIC Dosing Guideline for meloxicam recommends alternative therapy for CYP2C9 poor metabolizers due to markedly prolonged half-life, and initiating therapy with 50% of the lowest recommended starting dose or choose an alternative therapy for CYP2C9 intermediate metabolizers with activity score of 1. See full guideline for further details and supporting evidence. |
|  | **Recommendation:** Initiate therapy with recommended starting dose. In accordance with the prescribing information, use the lowest effective dosage for shortest duration consistent with individual patient treatment goals. |

### **mercaptopurine**

**Gene**: TPMT    **Diplotype**: \*1/\*1    **Phenotype**: Normal Metabolizer

|  |  |
| --- | --- |
| **CPIC** | **Summary:** Consider an alternate agent or extreme dose reduction of mercaptopurine for patients who are TPMT or NUDT15 poor metabolizers. Start at 30-80% of target dose for patients who are TPMT or NUDT15 intermediate metabolizers. |
|  | **Recommendation:** Start with normal starting dose (e.g., 75 mg/m2/day or 1.5 mg/kg/day) and adjust doses of mercaptopurine (and of any other myelosuppressive therapy) without any special emphasis on mercaptopurine compared to other agents. Allow at least 2 weeks to reach steady-state after each dose adjustment. |

**Gene**: NUDT15    **Diplotype**: \*1/\*1    **Phenotype**: Normal Metabolizer

|  |  |
| --- | --- |
| **CPIC** | **Summary:** Consider an alternate agent or extreme dose reduction of mercaptopurine for patients who are TPMT or NUDT15 poor metabolizers. Start at 30-80% of target dose for patients who are TPMT or NUDT15 intermediate metabolizers. |
|  | **Recommendation:** Start with normal starting dose (e.g., 75 mg/m2/day or 1.5 mg/kg/day) and adjust doses of mercaptopurine (and of any other myelosuppressive therapy) without any special emphasis on mercaptopurine compared to other agents. Allow at least 2 weeks to reach steady-state after each dose adjustment. |

### **methoxyflurane**

**Gene**: CACNA1S    **Diplotype**: Reference/Reference    **Phenotype**: Uncertain Susceptibility

|  |  |
| --- | --- |
| **CPIC** | **Summary:** The CPIC Dosing Guideline recommends that halogenated volatile anesthetics such as desflurane, enflurane, halothane, isoflurane, methoxyflurane, sevoflurane and the depolarizing muscle relaxants succinylcholine are relatively contraindicated in persons with malignant hyperthermia susceptibility (MHS). See full guideline for disclaimers, further details and supporting evidence. |
|  | **Recommendation:** These results do not eliminate the chance that this patient is susceptible to Malignant Hyperthermia. The genetic cause of about half of all MH survivors, with MH susceptibility confirmed by contracture test, remains unknown [Article:28902675]. |

**Gene**: RYR1    **Diplotype**: Reference/Reference    **Phenotype**: Uncertain Susceptibility

|  |  |
| --- | --- |
| **CPIC** | **Summary:** The CPIC Dosing Guideline recommends that halogenated volatile anesthetics such as desflurane, enflurane, halothane, isoflurane, methoxyflurane, sevoflurane and the depolarizing muscle relaxants succinylcholine are relatively contraindicated in persons with malignant hyperthermia susceptibility (MHS). See full guideline for disclaimers, further details and supporting evidence. |
|  | **Recommendation:** These results do not eliminate the chance that this patient is susceptible to Malignant Hyperthermia. The genetic cause of about half of all MH survivors, with MH susceptibility confirmed by contracture test, remains unknown [Article:28902675]. |

### **nortriptyline**

**Gene**: CYP2D6    **Diplotype**: \*1/\*2    **Phenotype**: Normal Metabolizer

|  |  |
| --- | --- |
| **CPIC** | **Summary:** The CPIC Dosing Guideline update for nortriptyline recommends a 25% dose reduction for CYP2D6 intermediate metabolizers. For CYP2D6 ultrarapid or poor metabolizers, an alternative drug should be considered. If nortriptyline is warranted, consider a 50% dose reduction in CYP2D6 poor metabolizers. |
|  | **Recommendation:** Initiate therapy with recommended starting dose. |

### **omeprazole**

**Gene**: CYP2C19    **Diplotype**: \*1/\*1    **Phenotype**: Normal Metabolizer

|  |  |
| --- | --- |
| **CPIC** | **Summary:** The CPIC Dosing Guideline for omeprazole, lansoprazole, pantoprazole recommends to increase the starting daily dose and to monitor efficacy in CYP2C19 ultrarapid metabolizer. For CYP2C19 rapid and normal metabolizers in the treatment of H. pylori infection and erosive esophagitis increasing the dose might be considered after initiation with the standard starting daily dose. The recommendations for intermediate and poor metabolizer for chronic therapy (>12 weeks) and efficacy achieved is to consider 50% reduction in daily dose. See full guideline for further details and supporting evidence. |
|  | **Recommendation:** Initiate standard starting daily dose. Consider increasing dose by 50-100% for the treatment of H. pylori infection and erosive esophagitis. Daily dose may be given in divided doses. Monitor for efficacy. |

### **ondansetron**

**Gene**: CYP2D6    **Diplotype**: \*1/\*2    **Phenotype**: Normal Metabolizer

|  |  |
| --- | --- |
| **CPIC** | **Summary:** The CPIC dosing guideline for ondansetron recommends selecting an alternate drug for CYP2D6 ultrarapid metabolizers. It is recommended that the alternate drug not be predominantly metabolized by CYP2D6 (eg. granisetron). |
|  | **Recommendation:** Initiate therapy with recommended starting dose. |

### **pantoprazole**

**Gene**: CYP2C19    **Diplotype**: \*1/\*1    **Phenotype**: Normal Metabolizer

|  |  |
| --- | --- |
| **CPIC** | **Summary:** The CPIC Dosing Guideline for omeprazole, lansoprazole, pantoprazole recommends to increase the starting daily dose and to monitor efficacy in CYP2C19 ultrarapid metabolizer. For CYP2C19 rapid and normal metabolizers in the treatment of H. pylori infection and erosive esophagitis increasing the dose might be considered after initiation with the standard starting daily dose. The recommendations for intermediate and poor metabolizer for chronic therapy (>12 weeks) and efficacy achieved is to consider 50% reduction in daily dose. See full guideline for further details and supporting evidence. |
|  | **Recommendation:** Initiate standard starting daily dose. Consider increasing dose by 50-100% for the treatment of H. pylori infection and erosive esophagitis. Daily dose may be given in divided doses. Monitor for efficacy. |

### **peginterferon alfa-2a**

**Gene**: IFNL3    **Diplotype**: C/C    **Phenotype**: -

|  |  |
| --- | --- |
| **CPIC** | **Summary:** IFNL3 (IL28B) variation (rs12979860) is the strongest baseline predictor of response to PEG-interferon-alpha-containing regimens in HCV genotype 1 patients. Patients with the favorable response genotype (rs12979860 CC) have increased likelihood of response (higher SVR rate) to PEG-interferon-alpha-containing regimens as compared to patients with unfavorable response genotype (rs12979860 CT or TT). Consider implications before initiating PEG-IFN alpha and RBV containing regimens. |
|  | **Recommendation:** Implications for PEG-IFN alpha and RBV: Approximately 70% chance for SVR after 48 weeks of treatment. Consider implications before initiating PEG-IFN alpha and RBV containing regimens. Implications for protease inhibitor combinations with PEG-IFN alpha and RBV therapy: Approximately 90% chance for SVR after 24-48 weeks of treatment. Approximately 80-90% of patients are eligible for shortened therapy (24-28 weeks vs. 48 weeks). Weighs in favor of using PEG-IFN alpha and RBV containing regimens. |

### **peginterferon alfa-2b**

**Gene**: IFNL3    **Diplotype**: C/C    **Phenotype**: -

|  |  |
| --- | --- |
| **CPIC** | **Summary:** IFNL3 (IL28B) variation (rs12979860) is the strongest baseline predictor of response to PEG-interferon-alpha-containing regimens in HCV genotype 1 patients. Patients with the favorable response genotype (rs12979860 CC) have increased likelihood of response (higher SVR rate) to PEG-interferon-alpha-containing regimens as compared to patients with unfavorable response genotype (rs12979860 CT or TT). Consider implications before initiating PEG-IFN alpha and RBV containing regimens. |
|  | **Recommendation:** Implications for PEG-IFN alpha and RBV: Approximately 70% chance for SVR after 48 weeks of treatment. Consider implications before initiating PEG-IFN alpha and RBV containing regimens. Implications for protease inhibitor combinations with PEG-IFN alpha and RBV therapy: Approximately 90% chance for SVR after 24-48 weeks of treatment. Approximately 80-90% of patients are eligible for shortened therapy (24-28 weeks vs. 48 weeks). Weighs in favor of using PEG-IFN alpha and RBV containing regimens. |

### **phenytoin**

**Gene**: CYP2C9    **Diplotype**: \*1/\*1    **Phenotype**: Normal Metabolizer

|  |  |
| --- | --- |
| **CPIC** | **Summary:** Phenytoin/fosphenytoin is contraindicated in individuals with the HLA-B\*15:02 variant allele ("HLA-B\*15:02-positive") due to significantly increased risk of phenytoin-induced cutaneous adverse reactions of Stevens-Johnson syndrome (SJS) and toxic epidermal necrolysis (TEN). Additionally, patients with the CYP2C9 poor metabolizer phenotype or with a CYP2C9 activity score of 1 may require reduced doses of phenytoin/fosphenytoin. |
|  | **Recommendation:** No adjustments needed from typical dosing strategies. Subsequent doses should be adjusted according to therapeutic drug monitoring, response, and side effects. An HLA-B\*15:02 negative test does not eliminate the risk of phenytoin-induced SJS/TEN, and patients should be carefully monitored according to standard practice. |

### **piroxicam**

**Gene**: CYP2C9    **Diplotype**: \*1/\*1    **Phenotype**: Normal Metabolizer

|  |  |
| --- | --- |
| **CPIC** | **Summary:** The CPIC Dosing Guideline for piroxicam recommends that CYP2C9 poor metabolizers and intermediate metabolizers with activity score of 1 should choose an alternative therapy not metabolized by CYP2C9 or not significantly impacted by CYP2C9 genetic variants in vivo or choose an NSAID metabolized by CYP2C9 but with a shorter half-life. See full guideline for further details and supporting evidence. |
|  | **Recommendation:** Initiate therapy with recommended starting dose. In accordance with the prescribing information, use the lowest effective dosage for shortest duration consistent with individual patient treatment goals. |

### **pitavastatin**

**Gene**: SLCO1B1    **Diplotype**: \*1/\*37    **Phenotype**: Normal Function

|  |  |
| --- | --- |
| **CPIC** | **Summary:** Prescribe ≤1mg as a starting dose for patients with SLCO1B1 poor function phenotype. Prescribe ≤2mg as a starting dose for patients with SLCO1B1 decreased or possible decreased phenotype. Adjust doses of pitavastatin based on disease-specific guidelines. Consider an alternative statin or combination therapy if higher doses are needed. |
|  | **Recommendation:** Prescribe desired starting dose and adjust doses based on disease-specific guidelines. |

### **pravastatin**

**Gene**: SLCO1B1    **Diplotype**: \*1/\*37    **Phenotype**: Normal Function

|  |  |
| --- | --- |
| **CPIC** | **Summary:** Prescribe ≤40mg as a starting dose and adjust doses of pravastatin based on disease-specific guidelines for patients with SLCO1B1 poor function phenotype. Prescribe desired starting dose and adjust doses of pravastatin based on disease-specific guidelines for patients with SLCO1B1 decreased or possible decreased phenotype. Prescriber should be aware of possible increased risk for myopathy with pravastatin especially with doses >40mg per day. |
|  | **Recommendation:** Prescribe desired starting dose and adjust doses based on disease-specific guidelines. |

### **rasburicase**

**Gene**: G6PD    **Diplotype**: B (wildtype)/B (wildtype)    **Phenotype**: Normal

|  |  |
| --- | --- |
| **CPIC** | **Summary:** Rasburicase is contraindicated in G6PD deficient patients with or without chronic non-spherocytic hemolytic anemia (CNSHA). In patients with a negative or inconclusive genetic test result an enzyme activity test is recommended prior to rasburicase treatment to determine whether a patient is G6PD deficient. The G6PD gene is X-linked and therefore males only have one copy, whereas females have two copies. See full guideline for disclaimers, further details and supporting evidence. |
|  | **Recommendation:** No reason to withhold rasburicase based on G6PD status. |

### **ribavirin**

**Gene**: IFNL3    **Diplotype**: C/C    **Phenotype**: -

|  |  |
| --- | --- |
| **CPIC** | **Summary:** IFNL3 (IL28B) variation (rs12979860) is the strongest baseline predictor of response to PEG-interferon-alpha-containing regimens in HCV genotype 1 patients. Patients with the favorable response genotype (rs12979860 CC) have increased likelihood of response (higher SVR rate) to PEG-interferon-alpha-containing regimens as compared to patients with unfavorable response genotype (rs12979860 CT or TT). Consider implications before initiating PEG-IFN alpha and RBV containing regimens. |
|  | **Recommendation:** Implications for PEG-IFN alpha and RBV: Approximately 70% chance for SVR after 48 weeks of treatment. Consider implications before initiating PEG-IFN alpha and RBV containing regimens. Implications for protease inhibitor combinations with PEG-IFN alpha and RBV therapy: Approximately 90% chance for SVR after 24-48 weeks of treatment. Approximately 80-90% of patients are eligible for shortened therapy (24-28 weeks vs. 48 weeks). Weighs in favor of using PEG-IFN alpha and RBV containing regimens. |

### **rosuvastatin**

**Gene**: SLCO1B1    **Diplotype**: \*1/\*37    **Phenotype**: Normal Function

|  |  |
| --- | --- |
| **CPIC** | **Summary:** Prescribe ≤20mg as a starting dose and adjust doses of rosuvastatin based on disease-specific and specific population guidelines for patients who are SLCO1B1 or ABCG2 poor function phenotype. If dose >20mg needed for desired efficacy, consider combination therapy (i.e. rosuvastatin plus non-statin guideline directed medical therapy). Patients with both ABCG2 poor function and SLCO1B1 poor/decreased function should be prescribed ≤10mg as a starting dose. |
|  | **Recommendation:** Prescribe desired starting dose and adjust doses based on disease-specific guidelines. |

### **sevoflurane**

**Gene**: RYR1    **Diplotype**: Reference/Reference    **Phenotype**: Uncertain Susceptibility

|  |  |
| --- | --- |
| **CPIC** | **Summary:** The CPIC Dosing Guideline recommends that halogenated volatile anesthetics such as desflurane, enflurane, halothane, isoflurane, methoxyflurane, sevoflurane and the depolarizing muscle relaxants succinylcholine are relatively contraindicated in persons with malignant hyperthermia susceptibility (MHS). See full guideline for disclaimers, further details and supporting evidence. |
|  | **Recommendation:** These results do not eliminate the chance that this patient is susceptible to Malignant Hyperthermia. The genetic cause of about half of all MH survivors, with MH susceptibility confirmed by contracture test, remains unknown [Article:28902675]. |

**Gene**: CACNA1S    **Diplotype**: Reference/Reference    **Phenotype**: Uncertain Susceptibility

|  |  |
| --- | --- |
| **CPIC** | **Summary:** The CPIC Dosing Guideline recommends that halogenated volatile anesthetics such as desflurane, enflurane, halothane, isoflurane, methoxyflurane, sevoflurane and the depolarizing muscle relaxants succinylcholine are relatively contraindicated in persons with malignant hyperthermia susceptibility (MHS). See full guideline for disclaimers, further details and supporting evidence. |
|  | **Recommendation:** These results do not eliminate the chance that this patient is susceptible to Malignant Hyperthermia. The genetic cause of about half of all MH survivors, with MH susceptibility confirmed by contracture test, remains unknown [Article:28902675]. |

### **simvastatin**

**Gene**: SLCO1B1    **Diplotype**: \*1/\*37    **Phenotype**: Normal Function

|  |  |
| --- | --- |
| **CPIC** | **Summary:** Prescribe an alternative statin depending on the desired potency for patients with SLCO1B1 decreased function, possible decreased function or poor function phenotype. If simvastatin therapy is warranted in patients with SLCO1B1 decreased or possible decreased phenotype, limit dose to <20mg/day. |
|  | **Recommendation:** Prescribe desired starting dose and adjust doses based on disease-specific guidelines. |

### **succinylcholine**

**Gene**: RYR1    **Diplotype**: Reference/Reference    **Phenotype**: Uncertain Susceptibility

|  |  |
| --- | --- |
| **CPIC** | **Summary:** The CPIC Dosing Guideline recommends that halogenated volatile anesthetics such as desflurane, enflurane, halothane, isoflurane, methoxyflurane, sevoflurane and the depolarizing muscle relaxants succinylcholine are relatively contraindicated in persons with malignant hyperthermia susceptibility (MHS). See full guideline for disclaimers, further details and supporting evidence. |
|  | **Recommendation:** These results do not eliminate the chance that this patient is susceptible to Malignant Hyperthermia. The genetic cause of about half of all MH survivors, with MH susceptibility confirmed by contracture test, remains unknown [Article:28902675]. |

**Gene**: CACNA1S    **Diplotype**: Reference/Reference    **Phenotype**: Uncertain Susceptibility

|  |  |
| --- | --- |
| **CPIC** | **Summary:** The CPIC Dosing Guideline recommends that halogenated volatile anesthetics such as desflurane, enflurane, halothane, isoflurane, methoxyflurane, sevoflurane and the depolarizing muscle relaxants succinylcholine are relatively contraindicated in persons with malignant hyperthermia susceptibility (MHS). See full guideline for disclaimers, further details and supporting evidence. |
|  | **Recommendation:** These results do not eliminate the chance that this patient is susceptible to Malignant Hyperthermia. The genetic cause of about half of all MH survivors, with MH susceptibility confirmed by contracture test, remains unknown [Article:28902675]. |

### **tacrolimus**

**Gene**: CYP3A5    **Diplotype**: \*1/\*3    **Phenotype**: Intermediate Metabolizer

|  |  |
| --- | --- |
| **CPIC** | **Summary:** The CPIC dosing guideline for tacrolimus recommends increasing the starting dose by 1.5 to 2 times the recommended starting dose in patients who are CYP3A5 intermediate or extensive metabolizers, though total starting dose should not exceed 0.3 mg/kg/day. Therapeutic drug monitoring should also be used to guide dose adjustments. |
|  | **Recommendation:** Increase starting dose 1.5 to 2 times recommended starting dose. Total starting dose should not exceed 0.3mg/kg/day. Use therapeutic drug monitoring to guide dose adjustments |

|  |  |
| --- | --- |
| **RNPGx** | **Summary:** Testing for the CYP3A5\*3 and CYP3A4\*22 alleles is recommended in patients receiving a kidney, heart or lung transplant. CYP3A extensive or intermediate meatbolizers (EMs or IMs) should be given an initial dose of tacrolimus 1.5-2 times higher than that recommended for CYP3A poor metabolizers (PMs), up to a maximum dose of 0.30 mg/kg/day. |
|  | **Recommendation:** Based on TDM, 1.5 to 2 times the dose recommended to non-expressors; maximum dose 0.30mg/kg/day. |

### **tamoxifen**

**Gene**: CYP2D6    **Diplotype**: \*1/\*2    **Phenotype**: Normal Metabolizer

|  |  |
| --- | --- |
| **CPNDS** | **Summary:** The Canadian Pharmacogenomics Network for Drug Safety (CPNDS) clinical recommendation group has published clinical practice guidelines for CYP2D6 as a treatment decision aid for ER-positive non-metastatic breast cancer patients. The guidelines recommend that alternatives to standard tamoxifen treatments may be considered in CYP2D6 poor or intermediate metabolizers with an emphasis on the use of comprehensive CYP2D6 genotyping panels in guiding treatment decisions. |
|  | **Recommendation:** aromatase inhibitor (AI) (with ovarian suppressor in premenopausal women) or tamoxifen (20 mg/day) or Tamoxifen (20 mg/day) when AIs are contraindicated |

|  |  |
| --- | --- |
| **CPIC** | **Summary:** The CPIC Dosing Guideline for tamoxifen recommends the use of alternative hormonal therapy such as an aromatase inhibitor for postmenopausal women or aromatase inhibitor along with ovarian function suppression in premenopausal women for CYP2D6 poor metabolizer, if aromatase inhibitor use is not contraindicated. For CYP2D6 intermediate metabolizers and CYP2D6 allele combinations resulting in an activity score (AS) of 1 the recommendation is to consider the recommendations stated for the CYP2D6 poor metabolizer. If aromatase inhibitor use is contraindicated, consideration should be given to use a higher but FDA approved tamoxifen dose for CYP2D6 intermediate metabolizers and CYP2D6 allele combinations resulting in an AS of 1. For poor metabolizer, higher dose tamoxifen (40 mg/day) increases but does not normalize endoxifen concentrations and can be considered if there are contraindications to aromatase inhibitor therapy. |
|  | **Recommendation:** Avoid moderate and strong CYP2D6 inhibitors. Initiate therapy with recommended standard of care dosing (tamoxifen 20 mg/day). |

### **tenoxicam**

**Gene**: CYP2C9    **Diplotype**: \*1/\*1    **Phenotype**: Normal Metabolizer

|  |  |
| --- | --- |
| **CPIC** | **Summary:** The CPIC Dosing Guideline for tenoxicam recommends that CYP2C9 poor metabolizers and intermediate metabolizers with activity score of 1 should choose an alternative therapy not metabolized by CYP2C9 or not significantly impacted by CYP2C9 genetic variants in vivo or choose an NSAID metabolized by CYP2C9 but with a shorter half-life. See full guideline for further details and supporting evidence. |
|  | **Recommendation:** Initiate therapy with recommended starting dose. In accordance with the prescribing information, use the lowest effective dosage for shortest duration consistent with individual patient treatment goals. |

### **thioguanine**

**Gene**: TPMT    **Diplotype**: \*1/\*1    **Phenotype**: Normal Metabolizer

|  |  |
| --- | --- |
| **CPIC** | **Summary:** Consider an alternate agent or extreme dose reduction of thioguanine for patients who are TPMT or NUDT15 poor metabolizers. Start at 50-80% of target dose for patients who are TPMT or NUDT15 intermediate metabolizers. |
|  | **Recommendation:** Start with normal starting dose (e.g. 40-60 mg/m2/day) and adjust doses of thioguanine and of other myelosuppressive therapy without any special emphasis on thioguanine. Allow 2 weeks to reach steady-state after each dose adjustment. |

**Gene**: NUDT15    **Diplotype**: \*1/\*1    **Phenotype**: Normal Metabolizer

|  |  |
| --- | --- |
| **CPIC** | **Summary:** Consider an alternate agent or extreme dose reduction of thioguanine for patients who are TPMT or NUDT15 poor metabolizers. Start at 50-80% of target dose for patients who are TPMT or NUDT15 intermediate metabolizers. |
|  | **Recommendation:** Start with normal starting dose (40-60 mg/day). Adjust doses of thioguanine and of other myelosuppressive therapy without any special emphasis on thioguanine. Allow 2 weeks to reach steady-state after each dose adjustment. |

### **tramadol**

**Gene**: CYP2D6    **Diplotype**: \*1/\*2    **Phenotype**: Normal Metabolizer

|  |  |
| --- | --- |
| **CPIC** | **Summary:** Alternate non-codeine analgesics are recommended for CYP2D6 ultrarapid and poor metabolizers. A label recommended age- or weight-specific dose of tramadol is warranted for CYP2D6 normal and intermediate metabolizers. |
|  | **Recommendation:** Use tramadol label recommended age- or weight-specific dosing |

### **trimipramine**

**Gene**: CYP2D6    **Diplotype**: \*1/\*2    **Phenotype**: Normal Metabolizer

|  |  |
| --- | --- |
| **CPIC** | **Summary:** Tricyclic antidepressants have comparable pharmacokinetic properties, it may be reasonable to apply the CPIC Dosing Guideline for amitriptyline and CYP2C19, CYP2D6 to other tricyclics including trimipramine. The CPIC Dosing Guideline update for amitriptyline recommends an alternative drug for CYP2D6 ultrarapid or poor metabolizers and CYP2C19 ultrarapid, rapid or poor metabolizers. If amitriptyline is warranted, consider a 50% dose reduction in CYP2D6 or CYP2C19 poor metabolizers. For CYP2D6 intermediate metabolizers, a 25% dose reduction should be considered. |
|  | **Recommendation:** Initiate therapy with recommended starting dose. |

**Gene**: CYP2C19    **Diplotype**: \*1/\*1    **Phenotype**: Normal Metabolizer

|  |  |
| --- | --- |
| **CPIC** | **Summary:** Tricyclic antidepressants have comparable pharmacokinetic properties, it may be reasonable to apply the CPIC Dosing Guideline for amitriptyline and CYP2C19, CYP2D6 to other tricyclics including trimipramine. The CPIC Dosing Guideline update for amitriptyline recommends an alternative drug for CYP2D6 ultrarapid or poor metabolizers and CYP2C19 ultrarapid, rapid or poor metabolizers. If amitriptyline is warranted, consider a 50% dose reduction in CYP2D6 or CYP2C19 poor metabolizers. For CYP2D6 intermediate metabolizers, a 25% dose reduction should be considered. |
|  | **Recommendation:** Initiate therapy with recommended starting dose. |

### **tropisetron**

**Gene**: CYP2D6    **Diplotype**: \*1/\*2    **Phenotype**: Normal Metabolizer

|  |  |
| --- | --- |
| **CPIC** | **Summary:** The CPIC dosing guideline for tropisetron recommends selecting an alternate drug for CYP2D6 ultrarapid metabolizers. It is recommended that the alternate drug not be predominantly metabolized by CYP2D6 (eg. granisetron). |
|  | **Recommendation:** Initiate therapy with recommended starting dose. |

### **voriconazole**

**Gene**: CYP2C19    **Diplotype**: \*1/\*1    **Phenotype**: Normal Metabolizer

|  |  |
| --- | --- |
| **CPIC** | **Summary:** The CPIC dosing guideline for voriconazole recommends selecting an alternative agent that is not dependent on CYP2C19 metabolism in adults who are CYP2C19 ultrarapid metabolizers, rapid metabolizers or poor metabolizers. In pediatric patients, an alternative agent should be used in patients who are ultrarapid metabolizers or poor metabolizers. In pediatric rapid metabolizers, therapy should be initiated at recommended standard case dosing, then therapeutic dosing monitoring should be used to titrate dose to therapeutic trough concentrations. |
|  | **Recommendation:** Initiate therapy with recommended standard of care dosing. |

### **warfarin**

**Gene**: CYP4F2    **Diplotype**: \*3/\*3    **Phenotype**: -

|  |  |
| --- | --- |
| **CPIC** | **Summary:** The updated guideline for pharmacogenetics-guided warfarin dosing is published by the Clinical Pharmacogenetics Implementation Consortium. The recommendations for dosing are for adult and pediatric patients that are specific to continental ancestry, and are based on genotypes from CYP2C9, VKORC1, CYP4F2, and rs12777823. |
|  | **Recommendation:** For non-African ancestry, if the CYP4F2\*3 (i.e., rs2108622, c.1297A, p.433Met) allele is also detected, increase the dose by 5-10%. |

**Gene**: VKORC1    **Diplotype**: T/T    **Phenotype**: -

|  |  |
| --- | --- |
| **CPIC** | **Summary:** The updated guideline for pharmacogenetics-guided warfarin dosing is published by the Clinical Pharmacogenetics Implementation Consortium. The recommendations for dosing are for adult and pediatric patients that are specific to continental ancestry, and are based on genotypes from CYP2C9, VKORC1, CYP4F2, and rs12777823. |
|  | **Recommendation:** A decreased dose of warfarin. Calculate warfarin dose using a validated pharmacogenetic algorithm [Articles:18305455, 19228618]. |

## **Diplotype Detail**

### **Multi-variant allele**

PAnno ranking model is applied to predict diplotypes consisting of multiple variants. The diplotypes are inferred by integrating allele definition consistency as well as the population allele frequency. PGx genes include CYP2B6, CYP2C19, CYP2C8, CYP2C9, CYP2D6, CYP3A4, CYP3A5, CYP4F2, DPYD, NUDT15, SLCO1B1, TPMT, and UGT1A1. Note that PAnno assumes that no variation occurs for the missing positions in the submitted VCF file.

### **CYP2B6: \*1/\*5**

Please notice that CYP2B6\*29, CYP2B6\*30 are not considered in the current version, which could potentially have an impact on the results.

| Position | Variant | Effect on Protein | Definition of \*1 | Definition of \*5 | Variant Call |
| --- | --- | --- | --- | --- | --- |
| chr19:40991224 | rs34223104 |  | T | T | Missing |
| chr19:40991367 | rs34883432 | p.Q21L | A | A | Missing |
| chr19:40991369 | rs8192709 | p.R22C | C | C | Missing |
| chr19:40991381 | rs33973337 | p.T26S | A | A | Missing |
| chr19:40991388 | rs33980385 | p.D28G | A | A | Missing |
| chr19:40991390 | rs33926104 | p.R29S | C | C | Missing |
| chr19:40991391 | rs34284776 | p.R29P | G | G | Missing |
| chr19:40991441 | rs35303484 | p.M46V | A | A | Missing |
| chr19:41004015 | rs281864907 | p.Y62X | T | T | Missing |
| chr19:41004125 | rs36060847 | p.G99E | G | G | Missing |
| chr19:41004158 | rs186335453 | p.G110V | G | G | Missing |
| chr19:41004303 | rs139801276 | p.I114T | T | T | Missing |
| chr19:41004377 | rs12721655 | p.K139E | A | A | Missing |
| chr19:41004381 | rs35773040 | p.R140Q | G | G | Missing |
| chr19:41004406 | rs145884402 | p.E148D | G | G | G/G |
| chr19:41006919 | rs3826711 | p.P167A | C | C | Missing |
| chr19:41006923 | rs36056539 | p.T168I | C | C | Missing |
| chr19:41006936 | rs3745274 | p.Q172H | G | G | Missing |
| chr19:41006968 | rs373489637 | p.V183G | T | T | Missing |
| chr19:41007013 | rs36079186 | p.M198T | T | T | T/T |
| chr19:41009350 | rs45482602 | p.S259R | C | C | Missing |
| chr19:41009358 | rs2279343 | p.K262R | A | A | A/A |
| chr19:41010006 | rs139029625 | p.A279P | G | G | Missing |
| chr19:41010088 | rs34698757 | p.T306S | C | C | Missing |
| chr19:41010108 | rs193922917 | p.L313I | C | C | Missing |
| chr19:41012316 | rs28399499 | p.I328T | T | T | Missing |
| chr19:41012339 | rs34826503 | p.R336C | C | C | Missing |
| chr19:41012465 | rs34097093 | p.R378X | C | C | Missing |
| chr19:41012693 | rs35979566 | p.I391N | T | T | Missing |
| chr19:41012740 | rs193922918 | p.A407T | G | G | Missing |
| chr19:41012803 | rs35010098 | p.P428T | C | C | Missing |
| chr19:41016726 | rs3211369 | p.M459V | A | A | Missing |
| chr19:41016778 | rs564083989 | p.G476D | G | G | Missing |
| chr19:41016805 |  | p.Q485L | A | A | Missing |
| chr19:41016810 | rs3211371 | p.R487S; p.R487C | C | T | C/T |

### **CYP2C8: \*1/\*1**

| Position | Variant | Effect on Protein | Definition of \*1 | Variant Call |
| --- | --- | --- | --- | --- |
| chr10:95067273 | rs11572080 | p.R139K | C | Missing |
| chr10:95067218 | rs72558196 | p.T159fs | T | Missing |
| chr10:95064931 | rs142886225 | p.G171S | C | C/C |
| chr10:95064901 | rs41286886 | p.V181I | C | Missing |
| chr10:95064886 | rs72558195 | p.R186G; p.R186X | G | Missing |
| chr10:95058485 |  | p.I223M | A | A/A |
| chr10:95058442 | rs188934928 | p.A238P | C | Missing |
| chr10:95058424 | rs11572102 | p.I244V | T | Missing |
| chr10:95058414 | rs769460274 | p.K247R | T | Missing |
| chr10:95058362 | rs1058930 | p.I264M | G | Missing |
| chr10:95058349 | rs11572103 | p.I269F | T | Missing |
| chr10:95045951 | rs78637571 | p.E274X | C | Missing |
| chr10:95043047 | rs146806199 | p.I331T | A | A/A |
| chr10:95042958 | rs45438799 | p.L361F | G | G/G |
| chr10:95042890 |  | p.K383N | C | Missing |
| chr10:95038992 | rs10509681 | p.K399R | T | Missing |
| chr10:95037219 | rs3832694 | p.461delV | ACA | Missing |

### **CYP2C9: \*1/\*1**

| Position | Variant | Effect on Protein | Definition of \*1 | Variant Call |
| --- | --- | --- | --- | --- |
| chr10:94942306 | rs1289704600 | p.A149V | C | Missing |
| chr10:94942308 | rs17847037 | p.R150C | C | Missing |
| chr10:94947439 |  | p.Q214H | G | Missing |
| chr10:94986136 | rs1254213342 | p.N418T | A | Missing |
| chr10:94942213 | rs1304490498 | p.K118fs | AGAAATGGAA | AGAAATGGAA/AGAAATGGAA |
| chr10:94949283 | rs9332131 | p.K273fs | A | A/A |
| chr10:94986042 | rs764211126 | p.I387V | A | Missing |
| chr10:94986073 | rs72558193 | p.D397A | A | Missing |
| chr10:94988852 | rs776908257 | p.R433W | C | C/C |
| chr10:94941976 |  | p.G96A | G | G/G |
| chr10:94942309 | rs7900194 | p.R150H; p.R150L | G | Missing |
| chr10:94947869 |  | p.D191G | A | Missing |
| chr10:94981302 | rs1250577724 | p.L361I | C | Missing |
| chr10:94938683 | rs114071557 | p.M1V | A | Missing |
| chr10:94938737 | rs67807361 | p.L19I | C | C/C |
| chr10:94938771 | rs142240658 | p.P30L | C | C/C |
| chr10:94938803 | rs2031308986 | p.N41D | A | Missing |
| chr10:94938828 | rs564813580 | p.D49G | A | Missing |
| chr10:94941897 | rs371055887 | p.G70R | G | Missing |
| chr10:94941915 |  | p.V76M | G | Missing |
| chr10:94941958 | rs72558187 | p.L90P | T | Missing |
| chr10:94941982 | rs762239445 | p.G98V | G | Missing |
| chr10:94942018 |  | p.F110S | T | Missing |
| chr10:94942216 | rs774607211 | p.K119R | A | Missing |
| chr10:94942230 | rs767576260 | p.R124W | C | Missing |
| chr10:94942231 | rs12414460 | p.R124Q | G | Missing |
| chr10:94942233 | rs375805362 | p.R125C | C | Missing |
| chr10:94942234 | rs72558189 | p.R125H; p.R125L | G | Missing |
| chr10:94942249 | rs200965026 | p.T130R; p.T130M | C | Missing |
| chr10:94942254 | rs199523631 | p.R132W | C | Missing |
| chr10:94942255 | rs200183364 | p.R132Q | G | Missing |
| chr10:94942290 | rs1799853 | p.R144C | C | Missing |
| chr10:94942291 | rs141489852 | p.R144H | G | Missing |
| chr10:94942305 | rs754487195 | p.A149T | G | G/G |
| chr10:94947782 | rs72558190 | p.S162X | C | Missing |
| chr10:94947785 | rs774550549 | p.P163L | C | Missing |
| chr10:94988855 |  | p.I434F | A | Missing |
| chr10:94981296 | rs1057910 | p.I359L | A | Missing |
| chr10:94988917 | rs769942899 | p.Q454H | G | Missing |
| chr10:94981225 | rs367826293 | p.R335Q | G | Missing |
| chr10:94981250 | rs750820937 | p.S343R | C | Missing |
| chr10:94988955 | rs767284820 | p.L467P | T | Missing |
| chr10:94981301 | rs28371686 | p.D360E | C | Missing |
| chr10:94989020 | rs9332239 | p.P489S | C | Missing |
| chr10:94981305 | rs578144976 | p.L362V | C | Missing |
| chr10:94988925 | rs202201137 | p.N457S | A | Missing |
| chr10:94988984 | rs781583846 | p.A477T | G | Missing |
| chr10:94989023 | rs868182778 | p.V490F | G | Missing |
| chr10:94981365 |  | p.P382S | C | Missing |
| chr10:94988880 |  | p.G442V | G | Missing |
| chr10:94947907 |  | p.N204H | A | Missing |
| chr10:94947917 | rs1326630788 | p.I207T | T | T/T |
| chr10:94947938 | rs2031531005 | p.Q214L | A | Missing |
| chr10:94949129 |  | p.I222V | A | Missing |
| chr10:94949144 |  | p.P227S | C | Missing |
| chr10:94949217 | rs2256871 | p.H251R | A | Missing |
| chr10:94949280 | rs9332130 | p.E272G | A | Missing |
| chr10:94972119 | rs182132442 | p.P279T | C | Missing |
| chr10:94972123 |  | p.S280C | C | Missing |
| chr10:94972134 |  | p.I284V | A | Missing |
| chr10:94972179 | rs72558192 | p.T299A | A | Missing |
| chr10:94972180 | rs988617574 | p.T299R | C | C/C |
| chr10:94972233 | rs1237225311 | p.P317S | C | Missing |
| chr10:94981199 |  | p.E326D | G | Missing |
| chr10:94981201 | rs57505750 | p.I327T | T | Missing |
| chr10:94981224 | rs28371685 | p.R335W | C | Missing |
| chr10:94981230 | rs1274535931 | p.P337T | C | Missing |
| chr10:94981281 | rs749060448 | p.E354K | G | Missing |
| chr10:94981297 | rs56165452 | p.I359T | T | Missing |
| chr10:94981371 | rs542577750 | splicing defect | G | Missing |

### **CYP2C19: \*1/\*1**

Please notice that CYP2C19\*36, CYP2C19\*37 are not considered in the current version, which could potentially have an impact on the results.

| Position | Variant | Effect on Protein | Definition of \*1 | Variant Call |
| --- | --- | --- | --- | --- |
| chr10:94761900 | rs12248560 | p.expression | C | Missing |
| chr10:94762706 | rs28399504 | p.M1V | A | Missing |
| chr10:94762712 | rs367543002 | p.P3S | C | C/C |
| chr10:94762715 | rs367543003 | p.F4L | T | Missing |
| chr10:94762755 | rs55752064 | p.L17P | T | Missing |
| chr10:94762760 | rs17882687 | p.I19L | A | Missing |
| chr10:94762788 | rs1564656981 | p.K28I | A | Missing |
| chr10:94762856 | rs1564657013 | p.S51G | A | Missing |
| chr10:94775106 | rs145328984 | p.R73C | C | Missing |
| chr10:94775121 | rs1564660997 | p.H78Y | C | Missing |
| chr10:94775160 | rs118203756 | p.G91R | G | Missing |
| chr10:94775185 | rs1288601658 | p.H99R | A | Missing |
| chr10:94775367 | rs12769205 | splicing defect | A | Missing |
| chr10:94775416 | rs41291556 | p.W120R | T | Missing |
| chr10:94775423 | rs17885179 | p.E122A | A | Missing |
| chr10:94775453 | rs72552267 | p.R132Q | G | Missing |
| chr10:94775489 | rs17884712 | p.R144H | G | Missing |
| chr10:94775507 | rs58973490 | p.R150H | G | Missing |
| chr10:94780574 | rs140278421 | p.R186P | G | G/G |
| chr10:94780579 | rs370803989 | p.D188N | G | Missing |
| chr10:94780653 | rs4986893 | p.W212X | G | Missing |
| chr10:94781858 | rs6413438 | p.P227L | C | Missing |
| chr10:94781859 | rs4244285 | splicing defect | G | G/G |
| chr10:94781944 | rs375781227 | p.D256N | G | G/G |
| chr10:94781999 | rs72558186 | splicing defect | T | Missing |
| chr10:94842861 | rs138142612 | p.R329H | G | Missing |
| chr10:94842866 | rs3758581 | p.I331V | G | G/G |
| chr10:94842879 | rs118203757 | p.R335Q | G | Missing |
| chr10:94842995 | rs113934938 | p.V374I | G | Missing |
| chr10:94849995 | rs17879685 | p.R410C | C | Missing |
| chr10:94852738 | rs56337013 | p.R433W | C | Missing |
| chr10:94852765 | rs192154563 | p.R442C | C | Missing |
| chr10:94852785 | rs118203759 | p.F448L | C | Missing |
| chr10:94852914 | rs55640102 | p.X491C | A | Missing |

### **CYP2D6: \*1/\*2**

Please notice that CYP2D6\*5, CYP2D6\*13, CYP2D6\*61, CYP2D6\*63, CYP2D6\*68 and CYP2D6 CNVs are not considered in the current version, which could potentially have an impact on the results.

| Position | Variant | Effect on Protein | Definition of \*1 | Definition of \*2 | Variant Call |
| --- | --- | --- | --- | --- | --- |
| chr22:42129042 | rs1135824 | p.N166D | T | T | Missing |
| chr22:42129033 | rs5030865 | p.G169R; p.G169X | C | C | Missing |
| chr22:42128945 | rs3892097 | splicing defect | C | C | Missing |
| chr22:42128934 | rs72549356 | p.174\_175insFRPx2; p.174\_175insFRP | AAAGGGGCG | AAAGGGGCG | Missing |
| chr22:42128878 |  | p.C191F | C | C | Missing |
| chr22:42128813 | rs150163869 |  | R | R | Missing |
| chr22:42128351 | rs377725912 | splicing defect | C | C | Missing |
| chr22:42130729 |  | p.L22X | G | G | G/G |
| chr22:42130655 | rs774671100 | p.L47fs | A | A | Missing |
| chr22:42129799 | rs76802407 | p.D97E | G | G | Missing |
| chr22:42129084 | rs5030655 | p.W152fs | A | A | Missing |
| chr22:42128817 | rs72549354 | p.L213fs | C | C | Missing |
| chr22:42128796 |  | p.L220fs | G | G | Missing |
| chr22:42128251 | rs72549353 | p.T256fs | TTAG | TTAG | Missing |
| chr22:42128242 | rs35742686 | p.R259fs | T | T | Missing |
| chr22:42128218 | rs72549352 | p.R269fs | G | G | Missing |
| chr22:42128199 | rs72549351 | p.T272fs | TCAG(2) | TCAG(2) | Missing |
| chr22:42128176 | rs5030656 | p.K281del | TCT | TCT | Missing |
| chr22:42127963 | rs267608279 | p.S288fs | G | G | Missing |
| chr22:42127846 | rs730882170 | p.M321fs | CACATCCGGATGTAGGATC | CACATCCGGATGTAGGATC | Missing |
| chr22:42126982 | rs757396767 | p.L395fs | AG | AG | Missing |
| chr22:42126658 | rs765776661 | p.468\_470dupVPT | AGTGGGCAC | AGTGGGCAC | Missing |
| chr22:42127973 | rs1135829 | p.N285S | T | T | Missing |
| chr22:42129809 | rs28371704 | p.H94R | T | T | Missing |
| chr22:42129770 | rs28371706 | p.Y107I; p.T107N | G | G | Missing |
| chr22:42129071 | rs267608302 | p.E156A; p.E156V | T | T | Missing |
| chr22:42128903 |  | p.S183X | del | del | Missing |
| chr22:42127922 | rs1406719554 | p.L302P | A | A | Missing |
| chr22:42129183 | rs374616348 | p.V119M | C | C | Missing |
| chr22:42130778 | rs773790593 | p.A5V | G | G | Missing |
| chr22:42130773 | rs72549358 | p.V7M | C | C | C/C |
| chr22:42130719 | rs267608313 | p.R25W | G | G | Missing |
| chr22:42130715 | rs28371696 | p.R26H | C | C | Missing |
| chr22:42130710 | rs138100349 | p.R28C | G | G | G/G |
| chr22:42130692 | rs1065852 | p.P34S | G | G | Missing |
| chr22:42130668 | rs5030862 | p.G42R | C | C | C/C |
| chr22:42130667 | rs118203758 | p.G42E | C | C | C/C |
| chr22:42129910 | rs201377835 | splicing defect | C | C | Missing |
| chr22:42129906 | rs267608311 | p.R62W | G | G | Missing |
| chr22:42129887 | rs1456026511 | p.V68G | A | A | Missing |
| chr22:42129836 | rs267608310 | p.A85V | G | G | Missing |
| chr22:42129827 | rs267608276 | p.R88P | C | C | Missing |
| chr22:42129821 | rs267608309 | p.A90V | G | G | Missing |
| chr22:42129819 | rs28371703 | p.L91M | G | G | Missing |
| chr22:42129780 | rs267608308 | p.V104M | C | C | Missing |
| chr22:42129779 | rs76187628 | p.V104A | A | A | Missing |
| chr22:42129771 | rs74802369 | p.T107S | T | T | Missing |
| chr22:42129765 | rs78459009 | p.I109V | T | T | Missing |
| chr22:42129759 | rs535642512 | p.G111S | C | C | Missing |
| chr22:42129180 | rs1135822 | p.F120I | A | A | Missing |
| chr22:42129174 | rs1135823 | p.A122S | C | C | Missing |
| chr22:42129166 | rs766391487 | p.Y124X | A | A | Missing |
| chr22:42129155 | rs1180015037 | p.W128X | C | C | Missing |
| chr22:42129134 | rs781457579 | p.S135F | G | G | Missing |
| chr22:42129132 | rs61736512 | p.V136M | C | C | Missing |
| chr22:42129130 | rs1058164 |  | S | S | S/G |
| chr22:42129113 | rs375135093 | p.L142S | A | A | Missing |
| chr22:42129098 | rs569229126 | p.K147R | T | T | Missing |
| chr22:42129087 | rs78482768 | p.Q151E | G | G | Missing |
| chr22:42129075 | rs28371710 | p.E155K | C | C | Missing |
| chr22:42129056 |  | p.C161S | C | C | Missing |
| chr22:42129037 | rs1135825 | p.H167Q | G | G | Missing |
| chr22:42129036 | rs1135826 | p.S168A | A | A | Missing |
| chr22:42126914 | rs28371733 | p.E418K | C | C | Missing |
| chr22:42127457 | rs77312092 | p.R388H | C | C | Missing |
| chr22:42126956 | rs1931013246 | p.K404Q | T | T | Missing |
| chr22:42127523 | rs1555888910 | p.F366S | A | A | Missing |
| chr22:42126896 | rs763964554 | p.Q424X | G | G | Missing |
| chr22:42127514 |  | p.I369T | A | A | Missing |
| chr22:42126877 | rs3021084 | p.P430L | G | G | Missing |
| chr22:42126752 | rs569439709 | p.G439D | C | C | Missing |
| chr22:42126749 | rs267608319 | p.R440H | C | C | Missing |
| chr22:42126747 | rs730882251 | p.R441C | G | G | Missing |
| chr22:42126746 | rs532668079 | p.R441H | C | C | Missing |
| chr22:42127512 | rs61745683 | p.V370I | C | C | Missing |
| chr22:42127473 | rs75386357 | p.E383K | C | C | Missing |
| chr22:42126735 | rs751092905 | p.G445R | C | C | Missing |
| chr22:42126719 | rs369177208 | p.R450H | C | C | Missing |
| chr22:42126697 |  | p.F457L | G | G | Missing |
| chr22:42126681 |  | p.H463D | G | G | Missing |
| chr22:42126663 | rs1135833 | p.P469A | G | G | Missing |
| chr22:42126660 | rs1135835 | p.T470A | T | T | Missing |
| chr22:42126647 | rs141756339 | p.R474Q | C | C | Missing |
| chr22:42126636 | rs28371735 | p.H478Y | G | G | Missing |
| chr22:42126635 | rs766507177 | p.H478P | T | T | Missing |
| chr22:42126634 |  | p.H478Q | A | A | Missing |
| chr22:42126633 |  | p.G479R | C | C | Missing |
| chr22:42126627 |  | p.F481V | A | A | Missing |
| chr22:42126624 | rs74478221 | p.A482T | C | C | Missing |
| chr22:42126623 | rs75467367 | p.A482G | G | G | Missing |
| chr22:42126611 | rs1135840 | p.S486T | C | G | C/G |
| chr22:42126605 | rs568495591 | p.S488F | G | G | Missing |
| chr22:42126578 | rs1440526469 | p.R497H | C | C | Missing |
| chr22:42128879 |  | p.C191R | A | A | Missing |
| chr22:42128848 | rs745365204 | p.R201H | C | C | Missing |
| chr22:42128812 | rs199535154 | p.L213P | A | A | Missing |
| chr22:42128329 | rs373813287 | p.L230F | G | G | Missing |
| chr22:42128325 | rs17002853 | p.L231P | A | A | Missing |
| chr22:42128308 | rs28371717 | p.A237S | C | C | Missing |
| chr22:42128272 |  | p.T249P | T | T | Missing |
| chr22:42128235 | rs267608297 | p.T261I | G | G | Missing |
| chr22:42128217 | rs148769737 | p.P267H | G | G | Missing |
| chr22:42128212 | rs367543000 | p.R269X | G | G | Missing |
| chr22:42128185 | rs77913725 | p.E278K | C | C | Missing |
| chr22:42128181 | rs1135828 | p.M279K | A | A | Missing |
| chr22:42127941 | rs16947 | p.R296C | G | A | G/A |
| chr22:42127938 | rs949717872 | p.I297L | T | T | Missing |
| chr22:42127899 |  | p.T310A | T | T | Missing |
| chr22:42127856 | rs5030867 | p.H324P | T | T | Missing |
| chr22:42127852 | rs79292917 | splicing defect | C | C | Missing |
| chr22:42127841 | rs72549349 | splicing defect | C | C | Missing |
| chr22:42127803 | rs28371725 | splicing defect | C | C | Missing |
| chr22:42127631 | rs141009491 | p.R330P | C | C | Missing |
| chr22:42127619 | rs72549348 | p.E334A | T | T | Missing |
| chr22:42127611 | rs78209835 | p.D337N | C | C | Missing |
| chr22:42127610 | rs748712690 | p.D337G | T | T | Missing |
| chr22:42127608 | rs59421388 | p.V338M | C | C | Missing |
| chr22:42127602 |  | p.G340R | C | C | Missing |
| chr22:42127593 | rs267608295 | p.R343G | G | G | Missing |
| chr22:42127590 | rs72549347 | p.R344X | G | G | Missing |
| chr22:42127589 | rs76088846 | p.R344Q | C | C | Missing |
| chr22:42127565 | rs61736517 | p.H352R | T | T | Missing |
| chr22:42127556 | rs202102799 | p.Y355C | T | T | Missing |
| chr22:42127530 | rs72549346 | p.Q364fs | del | del | Missing |
| chr22:42127526 | rs1058172 | p.R365H | C | C | Missing |
| chr22:42126938 | rs769157652 | p.E410K | C | C | Missing |
| chr22:42126926 | rs747089665 | p.R414C | G | G | Missing |
| chr22:42130761 | rs769258 | p.V11M | C | C | C/C |

### **CYP3A4: \*1/\*36**

| Position | Variant | Effect on Protein | Definition of \*1 | Definition of \*36 | Variant Call |
| --- | --- | --- | --- | --- | --- |
| chr7:99784075 | rs188389063 | p.L3V | G | G | Missing |
| chr7:99784038 | rs12721634 | p.L15P | A | A | Missing |
| chr7:99784018 | rs570051168 | p.L22V | G | G | Missing |
| chr7:99778079 | rs56324128 | p.G56D | C | C | Missing |
| chr7:99770217 | rs1449865051 | p.F113I | A | A | Missing |
| chr7:99770202 | rs55951658 | p.I118V | T | T | Missing |
| chr7:99770166 | rs778013004 | p.R130X | G | G | Missing |
| chr7:99770165 | rs72552799 | p.R130Q | C | C | Missing |
| chr7:99769805 | rs57409622 | p.R162W | G | G | Missing |
| chr7:99769804 | rs4986907 | p.R162Q | C | C | Missing |
| chr7:99769781 | rs72552798 | p.V170I | C | C | Missing |
| chr7:99769769 | rs4986908 | p.D174H | C | C | Missing |
| chr7:99768693 | rs35599367 | splicing defect | G | G | Missing |
| chr7:99768470 | rs12721627 | p.T185S | G | G | Missing |
| chr7:99768458 | rs4987161 | p.F189S | A | A | Missing |
| chr7:99768424 | rs113667357 | p.Q200H | T | T | T/T |
| chr7:99768371 | rs55901263 | p.P218R | G | G | Missing |
| chr7:99768360 | rs55785340 | p.S222P | A | A | Missing |
| chr7:99766440 | rs138105638 | p.R268X | G | G | Missing |
| chr7:99766412 | rs4646438 | p.D277fs | T | T | Missing |
| chr7:99764003 | rs28371759 | p.L293P | A | A | Missing |
| chr7:99763925 | rs201821708 | p.Y319C | T | T | Missing |
| chr7:99763909 | rs1303250043 | p.H324Q | G | G | Missing |
| chr7:99763877 | rs368296206 | p.I335T | A | A | Missing |
| chr7:99763843 | rs2242480 | p.expression | C | T | C/T |
| chr7:99762206 | rs67784355 | p.T363M | G | G | G/G |
| chr7:99762186 | rs756833413 | p.A370S | C | C | C/C |
| chr7:99762177 | rs12721629 | p.L373F | G | G | Missing |
| chr7:99762047 | rs4986909 | p.P416L | G | G | Missing |
| chr7:99760956 | rs774109750 | p.I427V | T | T | T/T |
| chr7:99760901 | rs4986910 | p.M445T | A | A | Missing |
| chr7:99760836 | rs4986913 | p.P467S | G | G | Missing |
| chr7:99758188 | rs67666821 | p.P488fs | T | T | Missing |

### **CYP3A5: \*1/\*3**

| Position | Variant | Effect on Protein | Definition of \*1 | Definition of \*3 | Variant Call |
| --- | --- | --- | --- | --- | --- |
| chr7:99652771 | rs41303343 | p.T346fs | A | A | Missing |
| chr7:99676198 | rs55817950 | p.R28C | G | G | Missing |
| chr7:99665212 | rs10264272 | splicing defect | C | C | Missing |
| chr7:99672916 | rs776746 | splicing defect | T | C | T/C |
| chr7:99660516 | rs28383479 | p.A337T | C | C | Missing |

### **CYP4F2: \*3/\*3**

| Position | Variant | Effect on Protein | Definition of \*3 | Variant Call |
| --- | --- | --- | --- | --- |
| chr19:15897578 | rs3093105 | p.W12G | A | Missing |
| chr19:15879621 | rs2108622 | p.V433M | T | T/T |

### **DPYD: Reference/Reference**

| Position | Variant | Effect on Protein | Definition of Reference | Variant Call |
| --- | --- | --- | --- | --- |
| chr1:97740411 | rs72549309 |  | ATGA(2) | Missing |
| chr1:97450067 | rs72549303 | p.P633Qfs | G | Missing |
| chr1:97699399 | rs72549307 | p.Y211C | T | Missing |
| chr1:97691776 | rs1801266 | p.R235W | G | Missing |
| chr1:97679170 | rs45589337 | p.K259E | T | Missing |
| chr1:97595149 | rs146356975 | p.K290E | T | Missing |
| chr1:97595088 | rs150437414 | p.L310S | A | Missing |
| chr1:97595083 | rs145112791 | p.L312F | G | Missing |
| chr1:97593379 | rs201018345 | p.A323T | C | C/C |
| chr1:97593343 | rs72549306 | p.V335L | C | Missing |
| chr1:97573839 | rs200064537 | p.N420K | A | Missing |
| chr1:97373629 | rs138545885 | p.A664S | C | Missing |
| chr1:97593289 | rs143154602 | p.R353C | G | Missing |
| chr1:97593238 | rs72549305 | p.I370V | T | Missing |
| chr1:97699533 | rs139834141 | p.M166I | C | Missing |
| chr1:97699506 | rs6670886 | p.S175S | C | Missing |
| chr1:97579893 | rs75017182 |  | G | Missing |
| chr1:97699474 | rs115232898 | p.Y186C | T | Missing |
| chr1:97573943 | rs78060119 | p.E386X | C | Missing |
| chr1:97573918 | rs143815742 | p.R394L | C | Missing |
| chr1:97573881 | rs61622928 | p.M406I | C | Missing |
| chr1:97573863 | rs56038477 |  | C | Missing |
| chr1:97883329 | rs1801265 | p.C29R | A | Missing |
| chr1:97573821 | rs764666241 | p.M426I | C | Missing |
| chr1:97573805 | rs142512579 | p.D432N | C | C/C |
| chr1:97573785 | rs186169810 | p.F438L | A | Missing |
| chr1:97549735 | rs72975710 | p.A450V | G | Missing |
| chr1:97549726 | rs144395748 | p.P453R | G | Missing |
| chr1:97549713 | rs57918000 | p.N457N | G | Missing |
| chr1:97549681 | rs199549923 | p.T468N | G | Missing |
| chr1:97515923 | rs148994843 | p.V515I | C | Missing |
| chr1:97549609 | rs72549304 | p.S492L | G | Missing |
| chr1:97549600 | rs111858276 | p.D495G | T | Missing |
| chr1:97549565 | rs138391898 | p.V507I | C | Missing |
| chr1:97515889 | rs190951787 | p.T526S | G | Missing |
| chr1:97515865 | rs1801158 | p.S534N | C | Missing |
| chr1:97515851 | rs142619737 | p.G539R | C | Missing |
| chr1:97515839 | rs1801159 | p.I543V | T | Missing |
| chr1:97515787 | rs55886062 | p.I560S | A | Missing |
| chr1:97515784 | rs201615754 | p.R561L | C | Missing |
| chr1:97450190 | rs59086055 | p.R592W | G | Missing |
| chr1:97450189 | rs138616379 | p.R592Q | C | Missing |
| chr1:97450187 | rs145773863 | p.G593R | C | Missing |
| chr1:97450168 | rs147601618 | p.M599T | A | A/A |
| chr1:97450059 | rs3918289 | p.N635K | G | Missing |
| chr1:97450058 | rs3918290 | splicing defect | C | Missing |
| chr1:97382461 | rs55971861 | p.I636L | T | T/T |
| chr1:97373598 | rs137999090 | p.G674D | C | Missing |
| chr1:97306195 | rs145548112 | p.A721T | C | Missing |
| chr1:97305372 | rs146529561 | p.A729V | G | Missing |
| chr1:97305364 | rs1801160 | p.V732I | C | C/C |
| chr1:97305363 | rs60511679 | p.V732G | A | Missing |
| chr1:97305279 | rs112766203 | p.T760I | G | Missing |
| chr1:97234991 | rs56005131 | p.T768K | G | Missing |
| chr1:97234958 | rs199634007 | p.T779N | G | Missing |
| chr1:97193209 | rs200687447 | p.E828K | C | Missing |
| chr1:97193109 | rs60139309 | p.K861R | T | Missing |
| chr1:97098632 | rs201035051 | p.K875Q | T | Missing |
| chr1:97079077 | rs202144771 | p.L993F | G | Missing |
| chr1:97740400 | rs150385342 | p.A105T | C | Missing |
| chr1:97079076 | rs139459586 | p.L993R | A | Missing |
| chr1:97079071 | rs1801268 | p.V995F | C | Missing |
| chr1:97079005 | rs140114515 | p.V1017I | C | C/C |
| chr1:97078993 | rs148799944 | p.V1021L | C | C/C |
| chr1:97078987 | rs114096998 | p.P1023T | G | G/G |
| chr1:97883368 | rs150036960 | p.L16V | G | Missing |
| chr1:97883353 | rs72549310 | p.R21X | G | Missing |
| chr1:97883352 | rs80081766 | p.R21Q | C | Missing |
| chr1:97721650 | rs141462178 | p.M115V | T | Missing |
| chr1:97721542 | rs200562975 | p.N151D | T | Missing |
| chr1:97699535 | rs2297595 | p.M166V | T | Missing |
| chr1:97593322 | rs183385770 | p.D342N | C | Missing |
| chr1:97079121 | rs72547601 | p.H978R | T | Missing |
| chr1:97450068 | rs17376848 | p.F632F | A | A/A |
| chr1:97098616 | rs55674432 | p.G880V | C | Missing |
| chr1:97098599 | rs147545709 | p.R886C | G | G/G |
| chr1:97098598 | rs1801267 | p.R886H | C | C/C |
| chr1:97699430 | rs72549308 | p.S201R | T | Missing |
| chr1:97573919 | rs140602333 | p.R394W | G | G/G |
| chr1:97082391 | rs67376798 | p.D949V | T | Missing |
| chr1:97082365 | rs141044036 | p.K958E | T | Missing |
| chr1:97079139 | rs145529148 | p.Q972R | T | Missing |
| chr1:97079133 | rs72547602 | p.D974V | T | Missing |

### **NUDT15: \*1/\*1**

| Position | Variant | Effect on Protein | Definition of \*1 | Variant Call |
| --- | --- | --- | --- | --- |
| chr13:48037826 | rs777311140 | p.C28fs | del | del/del |
| chr13:48037784 | rs746071566 | p.del17\_18GV; p.V18\_V19insGV | GAGTCG(3) | GAGTCG(3)/GAGTCG(3) |
| chr13:48040982 | rs1457579126 | p.N74fs | A | A/A |
| chr13:48041104 | rs761191455 | p.E115fs | G | Missing |
| chr13:48037847 | rs766023281 | p.R34T | G | G/G |
| chr13:48037748 | rs769369441 | p.M1T | T | Missing |
| chr13:48037749 |  | p.M1I | G | Missing |
| chr13:48037798 | rs186364861 | p.V18I | G | Missing |
| chr13:48037849 |  | p.K35E | A | Missing |
| chr13:48037885 | rs1950545307 | p.G47R | G | Missing |
| chr13:48037902 | rs149436418 | p.F52L | C | Missing |
| chr13:48041113 | rs1368252918 | p.E118X | G | Missing |
| chr13:48045690 | rs768324690 | p.P129R | C | Missing |
| chr13:48045719 | rs116855232 | p.R139C | C | Missing |
| chr13:48045720 | rs147390019 | p.R139H | G | Missing |
| chr13:48045771 | rs139551410 | p.L156Q | T | Missing |
| chr13:48037834 | rs1202487323 | p.L30V | C | Missing |

### **SLCO1B1: \*1/\*37**

Please notice that SLCO1B1\*48, SLCO1B1\*49 are not considered in the current version, which could potentially have an impact on the results.

| Position | Variant | Effect on Protein | Definition of \*1 | Definition of \*37 | Variant Call |
| --- | --- | --- | --- | --- | --- |
| chr12:21172734 | rs139257324 | p.R57W | C | C | Missing |
| chr12:21172776 | rs373327528 | p.G71R | G | G | G/G |
| chr12:21172782 | rs56101265 | p.F73L | T | T | Missing |
| chr12:21174595 | rs56061388 | p.V82A | T | T | Missing |
| chr12:21176804 | rs2306283 | p.N130D | A | G | A/G |
| chr12:21176868 | rs2306282 | p.N151S | A | A | A/A |
| chr12:21176871 |  | p.R152L | G | G | Missing |
| chr12:21176879 | rs11045819 | p.P155T | C | C | Missing |
| chr12:21176883 | rs72559745 | p.E156G | A | A | Missing |
| chr12:21176898 | rs77271279 | splicing defect | G | G | Missing |
| chr12:21178612 | rs141467543 | p.Y173C | A | A | Missing |
| chr12:21178615 | rs4149056 | p.V174A | T | T | Missing |
| chr12:21178957 | rs79135870 | p.I222V | A | A | A/A |
| chr12:21196951 | rs11045852 | p.I245V | A | A | Missing |
| chr12:21196975 | rs183501729 | p.R253X | C | C | Missing |
| chr12:21196976 | rs11045853 | p.R253Q | G | G | G/G |
| chr12:21200544 | rs72559747 | p.P336R | C | C | Missing |
| chr12:21200595 | rs55901008 | p.I353T | T | T | Missing |
| chr12:21202553 | rs1228465562 | p.F400V | T | T | T/T |
| chr12:21202555 | rs59113707 | p.F400L | C | C | C/C |
| chr12:21202649 | rs56387224 | p.N432D | A | A | Missing |
| chr12:21202664 | rs142965323 | p.G437R | G | G | G/G |
| chr12:21205921 | rs72559748 | p.D462G | A | A | Missing |
| chr12:21205999 | rs59502379 | p.G488A | G | G | Missing |
| chr12:21206031 | rs74064213 | p.I499V | A | A | Missing |
| chr12:21222355 | rs71581941 | p.R580X | C | C | Missing |
| chr12:21239042 | rs34671512 | p.L643F | A | A | Missing |
| chr12:21239077 | rs56199088 | p.D655G | A | A | Missing |
| chr12:21239113 | rs55737008 | p.E667G | A | A | A/A |
| chr12:21239145 | rs200995543 | p.H678Y | C | C | C/C |
| chr12:21239158 | rs140790673 | p.S682F | C | C | Missing |

### **TPMT: \*1/\*1**

| Position | Variant | Effect on Protein | Definition of \*1 | Variant Call |
| --- | --- | --- | --- | --- |
| chr6:18149127 | rs9333569 | p.M1V | T | Missing |
| chr6:18149126 | rs267607275 | p.M1T | A | A/A |
| chr6:18149045 | rs72552742 | p.E28V | T | Missing |
| chr6:18149032 | rs759836180 | p.K32KfsX58 | del | Missing |
| chr6:18149022 | rs750424422 | p.G36S | C | C/C |
| chr6:18149004 |  | p.Q42E | G | Missing |
| chr6:18147910 | rs72552740 | p.L49S | A | Missing |
| chr6:18147856 |  | p.F67S | A | Missing |
| chr6:18147851 | rs200591577 | p.L69V | G | Missing |
| chr6:18147845 | rs777686348 | p.G71R | C | Missing |
| chr6:18147838 | rs281874771 | p.A73V | G | Missing |
| chr6:18143724 | rs1800462 | p.A80P | C | Missing |
| chr6:18143718 | rs111901354 | p.R82W | G | Missing |
| chr6:18143700 | rs753545734 | p.G88S | C | C/C |
| chr6:18143643 |  | p.Y107D | A | Missing |
| chr6:18143622 | rs115106679 | p.E114K | C | Missing |
| chr6:18143613 |  | p.G117R | C | Missing |
| chr6:18143606 | rs151149760 | p.K119T | T | Missing |
| chr6:18143597 |  | p.K122T | T | Missing |
| chr6:18139710 | rs200220210 | p.S125L | G | Missing |
| chr6:18139689 | rs72552738 | p.C132Y | C | C/C |
| chr6:18139027 | rs72552737 | p.G144R | C | C/C |
| chr6:18138997 | rs1800460 | p.A154T | C | Missing |
| chr6:18138970 | rs112339338 | p.R163C | G | G/G |
| chr6:18133890 | rs9333570 |  | C | Missing |
| chr6:18133887 | rs201695576 | p.Y166C | T | Missing |
| chr6:18133884 | rs74423290 | p.A167G | G | Missing |
| chr6:18133870 | rs772832951 | p.S172P | A | Missing |
| chr6:18133847 | rs6921269 | p.Q179H | C | Missing |
| chr6:18133845 | rs75543815 | p.Y180F | T | Missing |
| chr6:18132163 |  | p.V199I | C | Missing |
| chr6:18132147 | rs79901429 | p.I204T | A | Missing |
| chr6:18132136 | rs72556347 | p.F208L | A | Missing |
| chr6:18130781 | rs1800584 |  | C | Missing |
| chr6:18130772 | rs377085266 | p.C212R | A | Missing |
| chr6:18130762 | rs56161402 | p.R215H | C | Missing |
| chr6:18130758 | rs398122996 | p.C216X | A | Missing |
| chr6:18130729 | rs139392616 | p.R226Q | C | Missing |
| chr6:18130725 | rs72552736 | p.H227Q | A | Missing |
| chr6:18130694 | rs150900439 | p.K238E | T | Missing |
| chr6:18130687 | rs1142345 | p.Y240C; p.Y240S | T | Missing |
| chr6:18143728 | rs1256618794 | p.W78C | C | Missing |
| chr6:18138969 | rs144041067 | p.R163H; p.R163P | C | C/C |

### **UGT1A1: \*28/\*80**

| Position | Variant | Effect on Protein | Definition of \*28 | Definition of \*80 | Variant Call |
| --- | --- | --- | --- | --- | --- |
| chr2:233759924 | rs887829 | 5' Flanking | C | T | C/T |
| chr2:233760235 | rs3064744 | 5' Flanking | TA(8) | TA(7) | TA(7)/CAT |
| chr2:233760498 | rs4148323 | p.G71R | G | G | G/G |
| chr2:233760973 | rs35350960 | p.P229Q | C | C | Missing |

### **Single-variant allele**

Single-variant alleles constitute diplotypes that do not involve the judgment of multiple variants and the corresponding genes generally have not yet been standardized by a nomenclature committee, such as rs9923231 for VKORC1.

| Gene | Variant | Variant Call |
| --- | --- | --- |
| - | rs12777823 | Missing |
| ABCG2 | rs2231142 | T/T |
| ACE | rs1799752 | A/A |
| ADD1 | rs4961 | G/G |
| ADRB2 | rs1042713 | A/A |
| ALDH2 | rs671 | G/A |
| APOE | rs7412 | Missing |
| ATIC | rs4673993 | T/C |
| CACNA1S | rs1800559 | Missing |
| CACNA1S | rs772226819 | G/G |
| CES1 | rs71647871 | Missing |
| CFTR | rs113993958 | Missing |
| CFTR | rs113993960 | Missing |
| CFTR | rs115545701 | Missing |
| CFTR | rs11971167 | Missing |
| CFTR | rs121908752 | Missing |
| CFTR | rs121908753 | G/G |
| CFTR | rs121908755 | Missing |
| CFTR | rs121908757 | Missing |
| CFTR | rs121909005 | T/T |
| CFTR | rs121909013 | Missing |
| CFTR | rs121909020 | Missing |
| CFTR | rs121909041 | Missing |
| CFTR | rs141033578 | Missing |
| CFTR | rs150212784 | T/T |
| CFTR | rs186045772 | T/T |
| CFTR | rs193922525 | G/G |
| CFTR | rs199826652 | Missing |
| CFTR | rs200321110 | Missing |
| CFTR | rs202179988 | Missing |
| CFTR | rs267606723 | Missing |
| CFTR | rs368505753 | Missing |
| CFTR | rs397508256 | Missing |
| CFTR | rs397508288 | Missing |
| CFTR | rs397508387 | Missing |
| CFTR | rs397508442 | Missing |
| CFTR | rs397508513 | Missing |
| CFTR | rs397508537 | Missing |
| CFTR | rs397508759 | Missing |
| CFTR | rs397508761 | Missing |
| CFTR | rs74503330 | Missing |
| CFTR | rs74551128 | Missing |
| CFTR | rs75039782 | C/C |
| CFTR | rs75527207 | Missing |
| CFTR | rs75541969 | Missing |
| CFTR | rs76151804 | A/A |
| CFTR | rs77834169 | Missing |
| CFTR | rs77932196 | Missing |
| CFTR | rs78655421 | Missing |
| CFTR | rs78769542 | Missing |
| CFTR | rs80224560 | Missing |
| CFTR | rs80282562 | G/G |
| CHRNA5 | rs16969968 | Missing |
| CYP2B6 | rs28399499 | Missing |
| CYP2B6 | rs3745274 | Missing |
| CYP3A4 | rs4646437 | Missing |
| CYP4F2 | rs2108622 | T/T |
| DPYD | rs115232898 | Missing |
| DPYD | rs148994843 | Missing |
| DPYD | rs17376848 | A/A |
| DPYD | rs1801158 | Missing |
| DPYD | rs1801159 | Missing |
| DPYD | rs1801160 | C/C |
| DPYD | rs1801265 | Missing |
| DPYD | rs1801266 | Missing |
| DPYD | rs1801268 | Missing |
| DPYD | rs2297595 | Missing |
| DPYD | rs3918290 | Missing |
| DPYD | rs55886062 | Missing |
| DPYD | rs56005131 | Missing |
| DPYD | rs56038477 | Missing |
| DPYD | rs59086055 | Missing |
| DPYD | rs67376798 | Missing |
| DPYD | rs72549303 | Missing |
| DPYD | rs72549306 | Missing |
| DPYD | rs72549309 | Missing |
| DPYD | rs75017182 | Missing |
| DPYD | rs78060119 | Missing |
| EGFR | rs121434568 | Missing |
| EGFR | rs121434569 | Missing |
| F5 | rs6025 | Missing |
| FCGR3A | rs396991 | Missing |
| HLA-A | \*15:02 | Missing |
| HLA-A | \*31:01 | Missing |
| HLA-A | \*31:01:02 | Zero copy |
| HLA-A | \*33:03 | Missing |
| HLA-B | \*13:01:01 | Zero copy |
| HLA-B | \*15:02 | Missing |
| HLA-B | \*15:02:01 | Zero copy |
| HLA-B | \*15:11 | Missing |
| HLA-B | \*15:11:01 | Zero copy |
| HLA-B | \*31:01 | Missing |
| HLA-B | \*38:02:01 | Zero copy |
| HLA-B | \*40:01:01 | Zero copy |
| HLA-B | \*57:01 | Missing |
| HLA-B | \*57:01:01 | Zero copy |
| HLA-B | \*58:01 | Missing |
| HLA-B | \*59:01:01:01 | Zero copy |
| HLA-C | \*01:02:01 | Zero copy |
| HLA-C | \*03:02 | Missing |
| HLA-C | \*04:01:01:01 | Zero copy |
| HLA-C | \*06:02:01:01 | Zero copy |
| HLA-C | \*07:27:01 | Missing |
| HLA-C | \*08:01 | Missing |
| HLA-DPB1 | \*03:01:01 | Missing |
| HLA-DRB1 | \*01:01:01 | Zero copy |
| IFNL3 | rs11881222 | A/A |
| IFNL3 | rs12979860 | Missing |
| IFNL3 | rs8099917 | Missing |
| IFNL4 | rs11322783 | Missing |
| IFNL4 | rs12979860 | Missing |
| ITPA | rs1127354 | Missing |
| ITPA | rs7270101 | Missing |
| KIF6 | rs20455 | A/G |
| MT-ND1 | rs267606617 | Missing |
| MT-RNR1 | rs267606617 | Missing |
| MT-RNR1 | rs267606618 | Missing |
| MT-RNR1 | rs267606619 | Missing |
| MTHFR | rs1801133 | Missing |
| NUDT15 | rs116855232 | Missing |
| RARG | rs2229774 | Missing |
| RYR1 | rs112563513 | G/G |
| RYR1 | rs118192116 | C/C |
| RYR1 | rs118192122 | Missing |
| RYR1 | rs118192124 | Missing |
| RYR1 | rs118192161 | Missing |
| RYR1 | rs118192162 | Missing |
| RYR1 | rs118192163 | Missing |
| RYR1 | rs118192167 | Missing |
| RYR1 | rs118192168 | Missing |
| RYR1 | rs118192170 | Missing |
| RYR1 | rs118192172 | Missing |
| RYR1 | rs118192175 | Missing |
| RYR1 | rs118192176 | Missing |
| RYR1 | rs118192177 | Missing |
| RYR1 | rs118192178 | Missing |
| RYR1 | rs121918592 | Missing |
| RYR1 | rs121918593 | Missing |
| RYR1 | rs121918594 | G/G |
| RYR1 | rs121918595 | Missing |
| RYR1 | rs121918596 | Missing |
| RYR1 | rs1801086 | Missing |
| RYR1 | rs193922747 | Missing |
| RYR1 | rs193922748 | Missing |
| RYR1 | rs193922753 | Missing |
| RYR1 | rs193922762 | Missing |
| RYR1 | rs193922764 | Missing |
| RYR1 | rs193922768 | Missing |
| RYR1 | rs193922770 | Missing |
| RYR1 | rs193922772 | Missing |
| RYR1 | rs193922802 | Missing |
| RYR1 | rs193922803 | Missing |
| RYR1 | rs193922807 | Missing |
| RYR1 | rs193922809 | Missing |
| RYR1 | rs193922816 | Missing |
| RYR1 | rs193922818 | G/G |
| RYR1 | rs193922832 | Missing |
| RYR1 | rs193922843 | Missing |
| RYR1 | rs193922876 | Missing |
| RYR1 | rs193922878 | Missing |
| RYR1 | rs28933396 | Missing |
| RYR1 | rs28933397 | Missing |
| RYR1 | rs63749869 | G/G |
| SCN1A | rs3812718 | C/T |
| SLC19A1 | rs1051266 | C/C |
| SLC28A3 | rs7853758 | Missing |
| SLCO1B1 | rs4149056 | Missing |
| TNF | rs1800629 | G/A |
| UGT1A1 | rs10929302 | G/A |
| VKORC1 | rs2359612 | A/A |
| VKORC1 | rs2884737 | A/A |
| VKORC1 | rs61742245 | Missing |
| VKORC1 | rs7294 | Missing |
| VKORC1 | rs8050894 | G/G |
| VKORC1 | rs9923231 | T/T |
| VKORC1 | rs9934438 | A/A |
| XPNPEP2 | rs3788853 | C/A |
| XRCC1 | rs25487 | C/C |

## **Phenotype Prediction**

For the clinically available drugs, PAnno integrates the effects of multiple diplotypes for each drug in terms of toxicity, dosage, efficacy, and metabolism. The predicted phenotypes are based on PharmGKB's high-confidence clinical annotations (evidence levels 1A, 1B, 2A, 2B) and are indicated as decreased, normal, and increased.

Drugs not further annotated due to "Avoid use": N/A.  
Drugs not included in clinical annotations used by PAnno: thioguanine, fosphenytoin.

| Drug | Toxicity | Dosage | Efficacy | Metabolism |
| --- | --- | --- | --- | --- |
| amitriptyline | ◎ Normal | - | - | ◎ Normal |
| atorvastatin | - | - | - | ◎ Normal |
| azathioprine | ◎ Normal | ◎ Normal | - | - |
| capecitabine | ◎ Normal | - | - | - |
| celecoxib | - | - | - | ◎ Normal |
| clomipramine | - | - | - | ◎ Normal |
| clopidogrel | ◎ Normal | - | ◎ Normal | ◎ Normal |
| codeine | ◎ Normal | - | - | ◎ Normal |
| desflurane | ⤋ Decreased | - | - | - |
| desipramine | - | - | - | ◎ Normal |
| dexlansoprazole | - | - | - | ◎ Normal |
| doxepin | - | - | - | ◎ Normal |
| enflurane | ⤋ Decreased | - | - | - |
| fluorouracil | ◎ Normal | - | - | - |
| flurbiprofen | - | - | - | ◎ Normal |
| fluvastatin | ◎ Normal | - | - | ◎ Normal |
| halothane | ⤋ Decreased | - | - | - |
| hydrocodone | - | - | - | ◎ Normal |
| ibuprofen | - | - | - | ◎ Normal |
| imipramine | - | - | - | ◎ Normal |
| isoflurane | ⤋ Decreased | - | - | - |
| lansoprazole | - | - | ◎ Normal | ◎ Normal |
| lornoxicam | - | - | - | ◎ Normal |
| meloxicam | - | - | - | ◎ Normal |
| mercaptopurine | ◎ Normal | ◎ Normal | - | - |
| methoxyflurane | ⤋ Decreased | - | - | - |
| nortriptyline | - | - | - | ◎ Normal |
| omeprazole | - | - | ◎ Normal | ◎ Normal |
| ondansetron | - | - | ◎ Normal | - |
| pantoprazole | - | - | ◎ Normal | ◎ Normal |
| peginterferon alfa-2a | - | - | ⤊ Increased | - |
| peginterferon alfa-2b | - | - | ⤊ Increased | - |
| phenytoin | ◎ Normal | - | - | ◎ Normal |
| piroxicam | - | - | - | ◎ Normal |
| pitavastatin | - | - | - | ◎ Normal |
| pravastatin | - | - | ⤊ Increased | - |
| ribavirin | - | - | ⤊ Increased | - |
| rosuvastatin | - | - | ⤊ Increased | ⤋ Decreased |
| sevoflurane | ⤋ Decreased | - | - | - |
| succinylcholine | ⤋ Decreased | - | - | - |
| tacrolimus | - | ⤋ Decreased | - | ⤋ Decreased |
| tamoxifen | - | - | ◎ Normal | ◎ Normal |
| tenoxicam | - | - | - | ◎ Normal |
| tramadol | ◎ Normal | - | - | ◎ Normal |
| trimipramine | - | - | - | ◎ Normal |
| voriconazole | - | - | - | ◎ Normal |
| warfarin | ⤊ Increased | ◎ Normal | ⤋ Decreased | - |

## **Clinical Annotation**

This section lists the clinical annotations on which the phenotype predictions are based.

| Drug | Category | Gene | Variant | Diplotype | Level | Phenotype | PharmGKB ID |  |
| --- | --- | --- | --- | --- | --- | --- | --- | --- |
| amitriptyline | Toxicity |  |  |  |  |  |  |  |
|  |  | CYP2D6 |  | \*1/\*2 | 1A | ◎ Normal | 1451259580 |  |
 Metabolism || CYP2C19 |  | \*1/\*1 | 1A | ◎ Normal | 1183617714 |
| CYP2D6 |  | \*1/\*2 | 1A | ◎ Normal | 1183617651 |
| atorvastatin | Metabolism |
| SLCO1B1 |  | \*1/\*37 | 1A | ◎ Normal | 1043880630 |
| azathioprine | Toxicity |
| TPMT |  | \*1/\*1 | 1A | ◎ Normal | 1451237240 |
 Dosage || TPMT |  | \*1/\*1 | 1A | ◎ Normal | 1451237326 |
| capecitabine | Toxicity |
| DPYD | rs17376848 | A/A | 1A | ◎ Normal | 1451287440 |
| DPYD | rs1801160 | C/C | 1A | ◎ Normal | 1451286040 |
| celecoxib | Metabolism |
| CYP2C9 |  | \*1/\*1 | 1A | ◎ Normal | 1451236700 |
| clomipramine | Metabolism |
| CYP2C19 |  | \*1/\*1 | 1A | ◎ Normal | 1183616699 |
| CYP2D6 |  | \*1/\*2 | 1A | ◎ Normal | 1043859155 |
| clopidogrel | Toxicity |
| CYP2C19 |  | \*1/\*1 | 1A | ◎ Normal | 1451282440 |
 Efficacy || CYP2C19 |  | \*1/\*1 | 1A | ◎ Normal | 1451282340 |
| CYP2C19 |  | \*1/\*1 | 1A | ◎ Normal | 1451282440 |
 Metabolism || CYP2C19 |  | \*1/\*1 | 1A | ◎ Normal | 1043858794 |
| codeine | Toxicity |
| CYP2D6 |  | \*1/\*2 | 1A | ◎ Normal | 1451288200 |
 Metabolism || CYP2D6 |  | \*1/\*2 | 1A | ◎ Normal | 1183616718 |
| desflurane | Toxicity |
| CACNA1S | rs772226819 | G/G | 1A | ⤋ Decreased | 1449563861 |
| RYR1 | rs112563513 | G/G | 1A | ⤋ Decreased | 1445400887 |
| RYR1 | rs118192116 | C/C | 1A | ⤋ Decreased | 1447676001 |
| RYR1 | rs121918594 | G/G | 1A | ⤋ Decreased | 1183705827 |
| RYR1 | rs193922818 | G/G | 1A | ⤋ Decreased | 1447673778 |
| RYR1 | rs63749869 | G/G | 1A | ⤋ Decreased | 1445400924 |
| desipramine | Metabolism |
| CYP2D6 |  | \*1/\*2 | 1A | ◎ Normal | 1183622099 |
| dexlansoprazole | Metabolism |
| CYP2C19 |  | \*1/\*1 | 1A | ◎ Normal | 1451137080 |
| doxepin | Metabolism |
| CYP2C19 |  | \*1/\*1 | 1A | ◎ Normal | 1183617854 |
| enflurane | Toxicity |
| CACNA1S | rs772226819 | G/G | 1A | ⤋ Decreased | 1451264060 |
| RYR1 | rs112563513 | G/G | 1A | ⤋ Decreased | 1445400887 |
| RYR1 | rs118192116 | C/C | 1A | ⤋ Decreased | 1447676001 |
| RYR1 | rs121918594 | G/G | 1A | ⤋ Decreased | 1183705827 |
| RYR1 | rs193922818 | G/G | 1A | ⤋ Decreased | 1447673778 |
| RYR1 | rs63749869 | G/G | 1A | ⤋ Decreased | 1445400924 |
| fluorouracil | Toxicity |
| DPYD | rs17376848 | A/A | 1A | ◎ Normal | 1183679833 |
| DPYD | rs1801160 | C/C | 1A | ◎ Normal | 1451286020 |
 Other || DPYD | rs1801160 | C/C | 1A | ⤊ Increased | 1445401793 |
| flurbiprofen | Metabolism |
| CYP2C9 |  | \*1/\*1 | 1A | ◎ Normal | 1444842106 |
| fluvastatin | Toxicity |
| CYP2C9 |  | \*1/\*1 | 1A | ◎ Normal | 1451678600 |
 Metabolism || CYP2C9 |  | \*1/\*1 | 1A | ◎ Normal | 1451666740 |
| halothane | Toxicity |
| CACNA1S | rs772226819 | G/G | 1A | ⤋ Decreased | 1451264081 |
| RYR1 | rs112563513 | G/G | 1A | ⤋ Decreased | 1445400887 |
| RYR1 | rs118192116 | C/C | 1A | ⤋ Decreased | 1447676001 |
| RYR1 | rs121918594 | G/G | 1A | ⤋ Decreased | 1183705827 |
| RYR1 | rs193922818 | G/G | 1A | ⤋ Decreased | 1447673778 |
| RYR1 | rs63749869 | G/G | 1A | ⤋ Decreased | 1445400924 |
| hydrocodone | Metabolism |
| CYP2D6 |  | \*1/\*2 | 1A | ◎ Normal | 1451141882 |
| ibuprofen | Metabolism |
| CYP2C9 |  | \*1/\*1 | 1A | ◎ Normal | 1451092720 |
| imipramine | Metabolism |
| CYP2C19 |  | \*1/\*1 | 1A | ◎ Normal | 1183617103 |
| isoflurane | Toxicity |
| CACNA1S | rs772226819 | G/G | 1A | ⤋ Decreased | 1451264087 |
| RYR1 | rs112563513 | G/G | 1A | ⤋ Decreased | 1445400887 |
| RYR1 | rs118192116 | C/C | 1A | ⤋ Decreased | 1447676001 |
| RYR1 | rs121918594 | G/G | 1A | ⤋ Decreased | 1183705827 |
| RYR1 | rs193922818 | G/G | 1A | ⤋ Decreased | 1447673778 |
| RYR1 | rs63749869 | G/G | 1A | ⤋ Decreased | 1445400924 |
| lansoprazole | Efficacy |
| CYP2C19 |  | \*1/\*1 | 1A | ◎ Normal | 1183629796 |
 Metabolism || CYP2C19 |  | \*1/\*1 | 1A | ◎ Normal | 1450806874 |
| lornoxicam | Metabolism |
| CYP2C9 |  | \*1/\*1 | 1A | ◎ Normal | 1183703296 |
| meloxicam | Metabolism |
| CYP2C9 |  | \*1/\*1 | 1A | ◎ Normal | 1451092677 |
| mercaptopurine | Toxicity |
| NUDT15 |  | \*1/\*1 | 1A | ◎ Normal | 1451448820 |
| TPMT |  | \*1/\*1 | 1A | ◎ Normal | 1184648909 |
 Dosage || NUDT15 |  | \*1/\*1 | 1A | ◎ Normal | 1448635217 |
| TPMT |  | \*1/\*1 | 1A | ◎ Normal | 1451237200 |
| methoxyflurane | Toxicity |
| CACNA1S | rs772226819 | G/G | 1A | ⤋ Decreased | 1451264120 |
| RYR1 | rs112563513 | G/G | 1A | ⤋ Decreased | 1445400887 |
| RYR1 | rs118192116 | C/C | 1A | ⤋ Decreased | 1447676001 |
| RYR1 | rs121918594 | G/G | 1A | ⤋ Decreased | 1183705827 |
| RYR1 | rs193922818 | G/G | 1A | ⤋ Decreased | 1447673778 |
| RYR1 | rs63749869 | G/G | 1A | ⤋ Decreased | 1445400924 |
| nortriptyline | Metabolism |
| CYP2D6 |  | \*1/\*2 | 1A | ◎ Normal | 1183618725 |
| omeprazole | Efficacy |
| CYP2C19 |  | \*1/\*1 | 1A | ◎ Normal | 1183630036 |
 Metabolism || CYP2C19 |  | \*1/\*1 | 1A | ◎ Normal | 1183630065 |
| ondansetron | Efficacy |
| CYP2D6 |  | \*1/\*2 | 1A | ◎ Normal | 982014094 |
| pantoprazole | Efficacy |
| CYP2C19 |  | \*1/\*1 | 1A | ◎ Normal | 1183624499 |
 Metabolism || CYP2C19 |  | \*1/\*1 | 1A | ◎ Normal | 1183624491 |
| peginterferon alfa-2a | Efficacy |
| IFNL3 | rs11881222 | A/A | 2A | ⤊ Increased | 1448102439 |
| peginterferon alfa-2b | Efficacy |
| IFNL3 | rs11881222 | A/A | 2A | ⤊ Increased | 1448102439 |
| phenytoin | Toxicity |
| CYP2C9 |  | \*1/\*1 | 1A | ◎ Normal | 981238501 |
 Metabolism || CYP2C9 |  | \*1/\*1 | 1A | ◎ Normal | 982047500 |
| piroxicam | Metabolism |
| CYP2C9 |  | \*1/\*1 | 1A | ◎ Normal | 1451092541 |
| pitavastatin | Metabolism |
| SLCO1B1 |  | \*1/\*37 | 1A | ◎ Normal | 1450814813 |
| pravastatin | Efficacy |
| KIF6 | rs20455 | A/G | 2B | ⤊ Increased | 655384621 |
| ribavirin | Efficacy |
| IFNL3 | rs11881222 | A/A | 2A | ⤊ Increased | 1448102439 |
| rosuvastatin | Efficacy |
| ABCG2 | rs2231142 | T/T | 2A | ⤊ Increased | 1154221922 |
 Metabolism || ABCG2 | rs2231142 | T/T | 1A | ⤋ Decreased | 1451666660 |
| SLCO1B1 |  | \*1/\*37 | 1A | ◎ Normal | 1043880701 |
| sevoflurane | Toxicity |
| CACNA1S | rs772226819 | G/G | 1A | ⤋ Decreased | 1451264140 |
| RYR1 | rs112563513 | G/G | 1A | ⤋ Decreased | 1445400887 |
| RYR1 | rs118192116 | C/C | 1A | ⤋ Decreased | 1447676001 |
| RYR1 | rs121918594 | G/G | 1A | ⤋ Decreased | 1183705827 |
| RYR1 | rs193922818 | G/G | 1A | ⤋ Decreased | 1447673778 |
| RYR1 | rs63749869 | G/G | 1A | ⤋ Decreased | 1445400924 |
| succinylcholine | Toxicity |
| CACNA1S | rs772226819 | G/G | 1A | ⤋ Decreased | 1451264128 |
| RYR1 | rs112563513 | G/G | 1A | ⤋ Decreased | 1445400887 |
| RYR1 | rs118192116 | C/C | 1A | ⤋ Decreased | 1447676001 |
| RYR1 | rs121918594 | G/G | 1A | ⤋ Decreased | 1183705827 |
| RYR1 | rs193922818 | G/G | 1A | ⤋ Decreased | 1447673778 |
| RYR1 | rs63749869 | G/G | 1A | ⤋ Decreased | 1445400924 |
| tacrolimus | Dosage |
| CYP3A5 |  | \*1/\*3 | 1A | ⤋ Decreased | 981203719 |
| CYP3A5 |  | \*1/\*3 | 2A | ⤋ Decreased | 1451243216 |
| CYP3A5 |  | \*1/\*3 | 2A | ⤋ Decreased | 1451241700 |
 Metabolism || CYP3A5 |  | \*1/\*3 | 1A | ⤋ Decreased | 1451214480 |
| CYP3A4 |  | \*1/\*36 | 1B | ⤊ Increased | 1183689931 |
| CYP3A5 |  | \*1/\*3 | 1B | ⤋ Decreased | 1451241780 |
| CYP3A5 |  | \*1/\*3 | 1B | ⤋ Decreased | 1184999911 |
| tamoxifen | Efficacy |
| CYP2D6 |  | \*1/\*2 | 1A | ◎ Normal | 1451285240 |
 Metabolism || CYP2D6 |  | \*1/\*2 | 1A | ◎ Normal | 982029857 |
| tenoxicam | Metabolism |
| CYP2C9 |  | \*1/\*1 | 1A | ◎ Normal | 1451092460 |
| tramadol | Toxicity |
| CYP2D6 |  | \*1/\*2 | 1A | ◎ Normal | 1451155125 |
 Metabolism || CYP2D6 |  | \*1/\*2 | 1A | ◎ Normal | 1451155020 |
| trimipramine | Metabolism |
| CYP2C19 |  | \*1/\*1 | 1A | ◎ Normal | 1183621987 |
| voriconazole | Metabolism |
| CYP2C19 |  | \*1/\*1 | 1A | ◎ Normal | 1183689217 |
| warfarin | Toxicity |
| CYP2C9 |  | \*1/\*1 | 1A | ◎ Normal | 1447672658 |
| CYP2C9 |  | \*1/\*1 | 1A | ◎ Normal | 1447672988 |
| VKORC1 | rs9923231 | T/T | 1B | ⤊ Increased | 1447673005 |
| VKORC1 | rs9923231 | T/T | 2A | ⤊ Increased | 1449269910 |
 Dosage || CYP2C9 |  | \*1/\*1 | 1A | ◎ Normal | 981238341 |
| CYP4F2 | rs2108622 | T/T | 1A | ⤊ Increased | 655385400 |
| VKORC1 | rs9923231 | T/T | 1A | ⤋ Decreased | 655385012 |
| VKORC1 | rs2359612 | A/A | 1B | ⤋ Decreased | 655385024 |
| VKORC1 | rs8050894 | G/G | 1B | ⤋ Decreased | 655385028 |
| VKORC1 | rs9934438 | A/A | 1B | ⤋ Decreased | 655385392 |
| VKORC1 | rs2884737 | A/A | 2A | ⤊ Increased | 982035703 |
 Efficacy || CYP2C9 |  | \*1/\*1 | 2A | ◎ Normal | 1447672600 |
| VKORC1 | rs9923231 | T/T | 2A | ⤋ Decreased | 1447672998 |
| VKORC1 | rs9923231 | T/T | 2A | ⤋ Decreased | 1447673015 |

## **About**

The report incorporates analyses of peer-reviewed studies and other publicly available information identified by PAnno by State Key Laboratory of Genetic Engineering from the School of Life Sciences and Human Phenome Institute, Fudan University, Shanghai, China. These analyses and information may include associations between a molecular alteration (or lack of alteration) and one or more drugs with potential clinical benefit (or potential lack of clinical benefit), including drug candidates that are being studied in clinical research.  
*Note:* A finding of biomarker alteration does not necessarily indicate pharmacologic effectiveness (or lack thereof) of any drug or treatment regimen; a finding of no biomarker alteration does not necessarily indicate lack of pharmacologic effectiveness (or effectiveness) of any drug or treatment regimen.  
*No Guarantee of Clinical Benefit:* This Report makes no promises or guarantees that a particular drug will be effective in the treatment of disease in any patient. This report also makes no promises or guarantees that a drug with a potential lack of clinical benefit will provide no clinical benefit.  
*Treatment Decisions are Responsibility of Physician:* Drugs referenced in this report may not be suitable for a particular patient. The selection of any, all, or none of the drugs associated with potential clinical benefit (or potential lack of clinical benefit) resides entirely within the discretion of the treating physician. Indeed, the information in this report must be considered in conjunction with all other relevant information regarding a particular patient, before the patient's treating physician recommends a course of treatment. Decisions on patient care and treatment must be based on the independent medical judgment of the treating physician, taking into consideration all applicable information concerning the patient's condition, such as patient and family history, physical examinations, information from other diagnostic tests, and patient preferences, following the standard of care in a given community. A treating physician's decisions should not be based on a single test, such as this test or the information contained in this report.  
When using results obtained from PAnno, you agree to cite PAnno.

**PAnno v0.3.1**
- Written by Yaqing Liu, et al.,
available at GitHub,
PyPI, and Conda.
  
Copyright © 2021-2022 Center for Pharmacogenomics, Fudan University, China. All Rights Reserved.
